# Detecting Clusters of Mutations

**DOI:** 10.1371/journal.pone.0003765

**Published:** 2008-11-19

**Authors:** Tong Zhou, Peter J. Enyeart, Claus O. Wilke

**Affiliations:** 1 Center for Computational Biology and Bioinformatics, Section of Integrative Biology, University of Texas at Austin, Austin, Texas, United States of America; 2 Institute for Cell and Molecular Biology, University of Texas at Austin, Austin, Texas, United States of America; University of California, Berkeley, United States of America

## Abstract

Positive selection for protein function can lead to multiple mutations within a small stretch of DNA, i.e., to a cluster of mutations. Recently, Wagner proposed a method to detect such mutation clusters. His method, however, did not take into account that residues with high solvent accessibility are inherently more variable than residues with low solvent accessibility. Here, we propose a new algorithm to detect clustered evolution. Our algorithm controls for different substitution probabilities at buried and exposed sites in the tertiary protein structure, and uses random permutations to calculate accurate *P* values for inferred clusters. We apply the algorithm to genomes of bacteria, fly, and mammals, and find several clusters of mutations in functionally important regions of proteins. Surprisingly, clustered evolution is a relatively rare phenomenon. Only between 2% and 10% of the genes we analyze contain a statistically significant mutation cluster. We also find that not controlling for solvent accessibility leads to an excess of clusters in terminal and solvent-exposed regions of proteins. Our algorithm provides a novel method to identify functionally relevant divergence between groups of species. Moreover, it could also be useful to detect artifacts in automatically assembled genomes.

## Introduction

Numerous methods have been proposed to identify positive selection in coding sequences [1]–[10]. These methods differ in their underlying assumptions, the data required to complete the analysis, and the type of conclusion that can be drawn. The most popular of these methods have in common that they are based in some form on the comparison of nonsynonymous to synonymous substitution frequencies, usually in the form of the ratio *dN*/*dS*. Their central premise is that synonymous substitutions are neutral and thus provide a baseline substitution rate to compare nonsynonymous substitutions against. Yet evidence is accumulating that synonymous substitutions often are not neutral. In particular, selection for translationally optimal codons operates from bacteria to mammals [11]–[17]. Other selective pressures on synonymous sites can arise from selection acting on mRNA secondary structure [18] or on exonic splicing enhancers [19]. For these and other reasons, *dN*/*dS*-based methods have been increasingly criticized [17], [20]–[22]; in particular, a recent study showed that sites known to be under positive selection for function are often not identified by *dN*/*dS* methods and vice versa [23]. Methods to detect positive selection that do not rely on synonymous substitution rates, such as Fu's *W* [24] or Tajima's *D* [25], are generally based on allele frequencies and thus are sensitive to demographic events, e.g., recent population bottlenecks [26]–[28].

Wagner has recently proposed a new method to detect positive selection that uses neither synonymous substitution rates nor allele frequencies [10]. Wagner suggested that strong positive selection will lead to multiple substitutions in close proximity, that is, it will lead to a clustering of nonsynonymous mutations in sequence space. He developed a statistical method to identify such clusters of mutations, and identified several cases of strong clustering of mutations in a comparison of human and chimpanzee genes.

Wagner's method constitutes an innovative and novel approach to a longstanding problem. Unfortunately, it suffers from three limitations. First, the *P* value Wagner assigns to a mutation cluster generally underestimates the probability that the cluster would arise by chance if the null hypothesis were true. Second, by design, Wagner's method can detect at most one variation cluster per gene. Third, and most importantly, Wagner's method does not control for inhomogeneous substitution rates caused by protein structure. The solvent accessibility of a site influences its evolutionary conservation, with more exposed residues generally being less conserved [29]–[34], and a method that does not consider this difference in baseline selective constraints in its null distribution will tend to find spurious mutation clusters in solvent-exposed regions of proteins.

In this study, we propose a novel method to detect mutation clusters that alleviates all three drawbacks. We use this method to locate mutation clusters in three groups of species: bacteria (*Escherichia coli* vs. *Salmonella enterica*), fly (*Drosophila melanogaster* vs. *Drosophila obscura*), and mammals (primates vs. rodents). We analyze the properties of the clusters we find and discuss how some of these clusters may affect protein function.

## Results

### A novel algorithm to detect mutation clusters

We begin by briefly reviewing Wagner's approach [10]. Wagner based his method on the probability *p*(*d_i_*_,*k*_) that, under the null hypothesis that all sites are equally likely to be mutated, *k* mutations arise within *d_i_*_,*k*_ or fewer residues, starting at the position of mutation *i*. The probability *p*(*d_i_*_,*k*_) can be calculated from the gamma distribution. For a given gene, Wagner calculated *p*(*d_i_*_,*k*_) for all possible contiguous sets of mutations in that gene, found the set with the minimum *p*(*d_i_*_,*k*_), and referred to this set as the gene's mutation cluster. He used the *p*(*d_i_*_,*k*_) value of this cluster, that is, *P_P_* = min*_i_*_,*k*_ *p*(*d_i_*_,*k*_), as the cluster's *P* value.

Wagner's approach has two (arguably minor) statistical problems. First, because of the minimization procedure, *P_P_* is not the probability that the associated cluster would arise if the null hypothesis were true. The probability *p*(*d_i_*_,*k*_) measures the likelihood that, under the null hypothesis, a randomly chosen contiguous set of *k* mutations falls within at most *d_i_*_,*k*_ residues. Consequently, *P_P_* underestimates the probability that the most-clustered set of mutations (i.e., the cluster corresponding to min*_i_*_,*k*_ *p*(*d_i_*_,*k*_)) falls within at most *d_i_*_,*k*_ residues by chance alone. We can make this reasoning more intuitive by considering the general situation of a set of multiple events that occur according to some probability distribution. The most extreme of these events has a higher probability of being extreme than each event has individually. Second, by focusing on the cluster with the minimum *p*(*d_i_*_,*k*_), Wagner can never detect more than one cluster per gene, even if a second, highly significant cluster is present.

It would be straightforward to fix these two statistical problems with a minor modification to Wagner's approach. But we are here primarily interested in a third, more fundamental limitation. We believe that the null hypothesis of a single, homogeneous mutation probability throughout the protein does not reflect biological reality and will lead to spurious mutation clusters. It is well known that amino-acid substitution rates correlate with solvent accessibility [29]–[33], [35]. Substitutions at buried sites are more likely to be disruptive than substitutions at exposed sites, and are therefore more strongly selected against. If we don't control for this effect when searching for mutation clusters, we are likely to identify clusters in highly variable and relatively unimportant loop regions. It is unlikely that such clusters represent positive selection; they simply represent regions of weak selective constraint.

We now describe a method to detect mutation clusters that controls for solvent accessibility and that does not suffer from the two statistical problems outlined above. Instead of building our algorithm on the probability that, under the null hypothesis, *k* mutations arise within *n* or fewer residues, we consider instead the probability that *k* or more mutations fall within exactly *n* residues. This probability is binomial. (See Methods for details. Note that we use *n* instead of Wagner's *d* throughout the remainder of this paper.) By keeping the number (and location) of the residues fixed, we can easily generalize our algorithm to situations where different sites have different mutation probabilities. In the present work, we distinguish only between buried and exposed sites, but more complicated models would be feasible.

**Figure 1:**
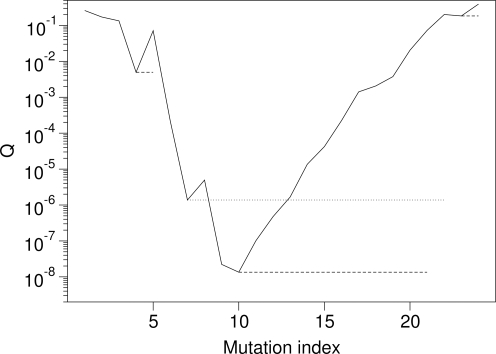
*Q*-landscape of the *E. coli* gene *frdA* (fumarate reductase flavoprotein subunit). *Q* has four local minima. Thus we have four potential clusters, starting at mutation numbers 4, 7, 10, and 23. The horizontal lines show the range of each potential cluster. The second cluster (dotted line) overlaps with the third cluster, which has lower *Q*. Therefore, we exclude the second cluster and obtain three potential mutation clusters (dashed lines). After correction for multiple testing, only one significant cluster remains, the one starting at position 10.

Our algorithm assumes that we are given two pieces of information for each gene to be analyzed: the location of all amino-acid mutations in the gene, and the solvent accessibility (measured as either buried or exposed) of each residue in the translated and folded protein. We then calculate the fraction of mutations for buried and exposed sites, *f_b_* and *f_e_*, and use these values to parameterize our binomial model. Thus we calculate the probability *q*(*k*; *n_e_*, *n_b_*, *f_e_*, *f_b_*) to observe at least *k* mutations within a given stretch of *n* residues composed of *n_b_* buried and *n_e_* exposed residues (Eq. 3 in Methods). We calculate *q*(*k*; *n_e_*, *n_b_*, *f_e_*, *f_b_*) for all possible contiguous sets of mutations (i.e., possible clusters) in the gene, and for each mutation, record the minimum-*q*(*k*; *n_e_*, *n_b_*, *f_e_*, *f_b_*) value of all possible clusters starting with this mutation. We refer to the minimum-*q*(*k*; *n_e_*, *n_b_*, *f_e_*, *f_b_*) value as *Q*, and to the total set of *Q* values as *Q*-landscape (Fig. 1). Local minima in the *Q*-landscape correspond to potential mutation clusters. We then discard all potential clusters that overlap with any other potential cluster with lower *Q* (Fig. 1).

We now have a set of potential clusters for the gene, and the next step is to calculate a *P* value for each cluster. For a given cluster for which we want to calculate *P*, we use the *Q* value defined above as test statistic, and denote it as *Q_s_*. We then randomly reshuffle the mutations in the gene, repeat our analysis of finding non-overlapping clusters at minima in the *Q*-landscape, and record the frequency with which *Q_s_*<*Q*. This frequency is the cluster's *P* value.

Because we are finding many potential clusters (we may find multiple clusters per gene, and we are analyzing hundreds of genes), we use the false-discovery-rate correction [36] to correct for multiple testing. We refer to the corrected *P* value as *P_M_*, and to the uncorrected *P* value as *P_U_*. Throughout this work, we consider potential clusters with *P_M_*<0.05 as significant.

### Mutation clusters in bacteria, fly, and mammals

In principle, we can apply our method to any pair of orthologous sequences, such as a human sequence and the corresponding ortholog in macaque. But when we carried out genome-wide scans for clusters of mutations between pairs of species, we found numerous clusters that, upon closer inspection, appeared to be artifacts in one of the species. In particular, we found numerous clusters in the macaque genome that seemed to stem from errors in the assembly of the draft genome rather than representing true sequence differences (data not shown). Therefore, we decided to compare pairs of species and considered only those mutations that were conserved within each pair but differed among pairs.

**Figure 2:**
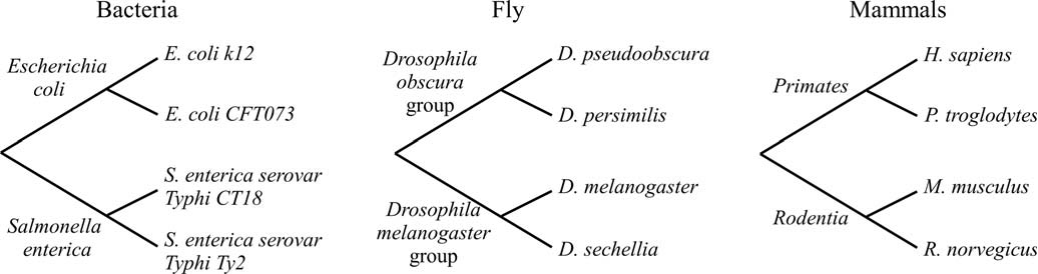
Species considered in this work. For each set of four species, we only considered mutations that were conserved within the upper and lower branch but differed between these two branches, and searched for clustered occurrences of these mutations. Branch lengths are not to scale.

We carried out scans for mutation clusters in bacteria (two species of *E. coli* compared to two species of *S. enterica*), fly (two species of the group *D. obscura* compared to two species of the group *D. melanogaster*), and mammals (two species of primates compared to two species of rodents). See Fig. 2 for details. To obtain the solvent accessibility data required for our analysis, we mapped all sequences to homologous sequences with known structure in the PDB, and discarded genes for which we could not find any reliable structure information. The final data sets contained 356, 99, and 246 genes for bacteria, fly, and mammals, respectively.

Controlling for differences in evolutionary rate at buried and exposed sites as described above, we found a total of 1255, 246, and 868 potential mutation clusters in bacteria, fly, and mammals. Of these, in bacteria there were 31 significant clusters after correction for multiple testing. There were 181 clusters for which the uncorrected *P_U_* was less than 5%. In fly, there were 6 significant clusters (48 with *P_U_*<0.05). In mammals, there were 5 significant clusters (87 clusters with *P_U_*<0.05).

### Statistical properties of mutation clusters

We next analyzed whether the mutation clusters differed in some aspect from the protein regions that did not display clustered mutations. First, we considered solvent accessibility. We calculated the fraction of mutations at buried sites within and outside of mutation clusters, and carried out a paired *t*-test to determine whether clustered mutations were more or less buried than non-clustered mutations. We jointly considered all mutation clusters for all species groups, as long as there was at least one mutation in the gene outside the cluster, and found a mean difference in the fraction of buried sites in clustered and non-clustered mutations of 0.061 (*P* = 0.077, *n* = 41). Thus, after controlling for solvent accessibility, mutation clusters are roughly equally likely to appear in buried or in exposed regions of the protein.

Second, we tested whether mutation clusters were associated predominantly with specific secondary structure motifs. We computed the fraction of sites with secondary structure of the types helix, sheet, turn, and coil, both inside and outside of mutation clusters, and found no significant differences (paired *t*-test *P* = 0.855 for helix, *P* = 0.392 for sheet, *P* = 0.882 for turn, and *P* = 0.454 for coil, *n* = 42). Therefore, secondary structure composition does not seem to affect the location of mutation clusters.

Finally, we considered physicochemical distance for clustered and non-clustered mutations. Some authors have proposed that positive selection leads to physicochemically radical amino acid replacements [37], [38] (but see [39]–[41]). Here, we considered five amino acid properties that have been found to correlate with rates of amino acid replacement [42], [43]: composition of the side chain, polarity, and molecular volume [44], as well as hydropathy [45] and isoelectric point [46]. For each of these properties, we calculated for each gene the mean distance for mutations within a cluster and mutations outside of the cluster, and then tested for a non-zero mean distance using a paired *t*-test. We found one marginally significant result: mutations within clusters tend to have a more radical molecular-volume change than mutations outside of clusters (*P* = 0.024, *n* = 41), but the magnitude of the effect was small. The mean difference in the absolute volume change for mutations inside and outside of clusters was 3.78. The molecular-volume scale ranges from 3 (glycine) to 170 (tryptophan), with the majority of amino acids falling between 30 and 130 [44]. For the other four properties, differences were not significant (side chain composition, *P* = 0.816; polarity, *P* = 0.157; hydropathy, *P* = 0.134; isoelectric point, *P* = 0.167).

### The effect of solvent accessibility on cluster location

Buried residues experience more purifying selection than exposed residues [29]–[33]. Therefore, an algorithm that doesn't control for solvent accessibility should find mutation clusters predominantly in exposed areas of the protein.

**Figure 3:**
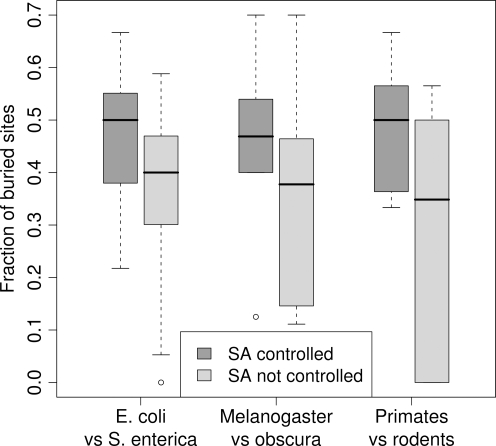
Fraction of buried sites in significant clusters obtained by either controlling or not controlling for solvent accessibility (SA). If solvent accessibility is not controlled for, many of the resulting clusters are located in exposed regions of the protein.

To determine the effect of solvent accessibility on the mutation clusters, we repeated our analysis but ignored protein structure, by artificially assigning to all residues the “buried” status in our cluster detection program. (We could have chosen the “exposed” status with identical results. What matters is that all sites have the same status.) In this case, we found 47 significant clusters in bacteria, 12 in fly, and 6 in mammals. We then calculated the fraction of buried sites within each significant cluster, and compared this fraction for clusters determined with and without controlling for solvent accessibility. We found that clusters determined without controlling for solvent accessibility tend to have fewer buried sites, i.e., are more exposed (two-sample *t*-test on pooled data from all three species groups, *P*<0.001, see also Fig. 3).

**Figure 4:**
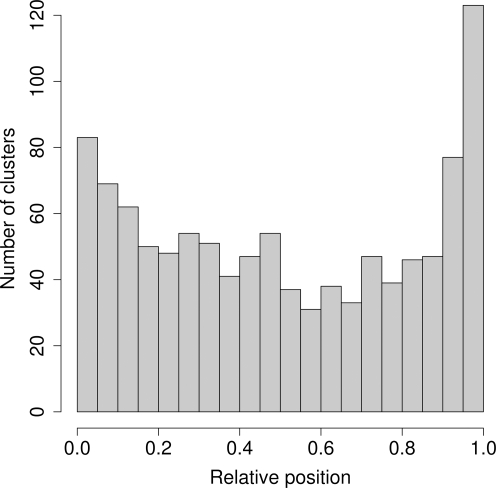
Distribution of cluster positions, for *E. coli* clusters found without controlling for solvent accessibility. The relative cluster position was calculated by dividing the cluster's central coordinate by the total sequence length. The cluster positions are not uniformly distributed, and clusters are most frequent in terminal regions of proteins.

For bacteria, we also used our algorithm to calculate mutation clusters for all ORFs, regardless of whether we had protein structures for them or not, again artificially treating all residues as buried. We found 1070 significant clusters out of 13642 potential clusters. We then determined where within each coding sequence the mutation clusters were located, and found that the distribution of cluster locations along the coding sequence was not uniform (χ^2^-test, *P*<10^−10^). Clusters appeared more frequently on the termini (within 10% of total sequence length), as shown in Fig. 4. This result agrees with our hypothesis that solvent exposure can lead to spurious mutation clusters. Terminal regions of proteins (i.e., the N- and C-termini) are predominantly located on the protein surface and are exposed to the solvent [47]–[49]. By contrast, when controlling for solvent accessibility, we found only 2 out of 20 significant clusters within 10% (in terms of total sequence length) of the C-terminus, and none out of 12 significant clusters within 10% of the N-terminus. For this analysis, we considered only those proteins for which the terminal region of interest was not truncated in the PDB structure. Therefore, we had only 20 clusters whose location we could determine relative to the C-terminus, and 12 relative to the N-terminus.

### Example mutation clusters

Supplementary Tables S1, S2, and S3 list the significant mutation clusters in the three species groups. The mutation clusters span on average 9.2%, 7.3%, and 3.8% (numbers are for bacteria, fly, and mammals) of the coding regions in which they appear. Supplementary Figures S1.1–S1.39 show how each cluster maps onto the corresponding protein structure. The figures also show each cluster in the context of a multi-species sequence alignment. Supplementary Text S1 provides an overview over all supplementary materials.

**Figure 5:**
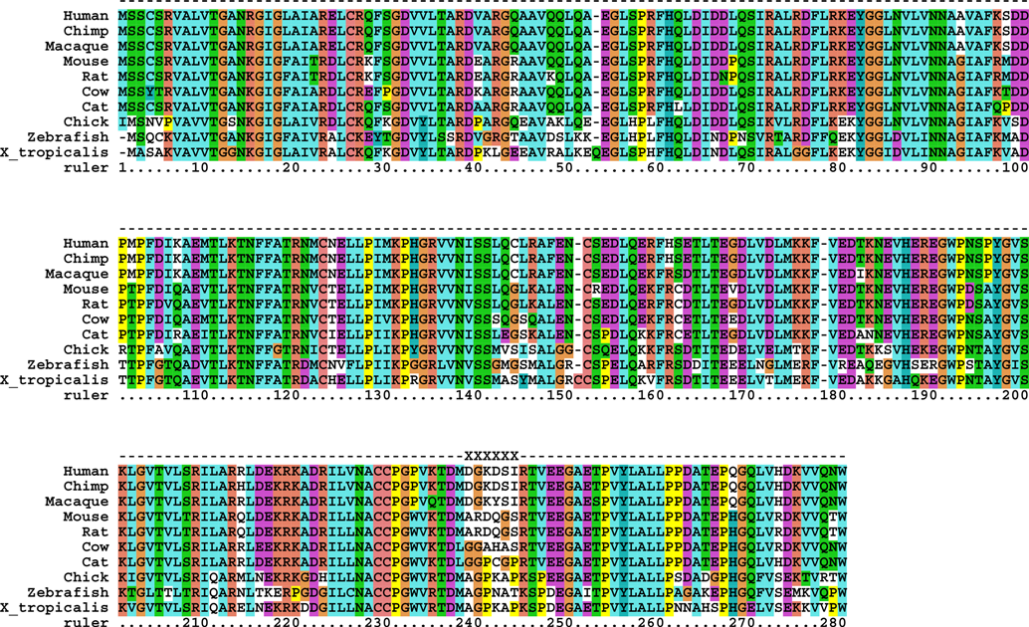
Multiple sequence alignment of the human protein CBR3 and its orthologs in chimpanzee, macaque, mouse, rat, cow, cat, chicken, zebrafish and *Xenopus tropicalis*. The mutation cluster spans from position 239 to position 244 and is marked by the symbol X.

We now discuss two examples of mutation clusters. The first example is the human enzyme carbonyl reductase 3 (CBR3, ENSG00000159231), which catalyzes the NADPH-dependent reduction of a variety of xenobiotic ketones and quinones [50], [51]. The mutation cluster in CBR3 runs from position 239 to position 244. It is fully conserved within primates and within rodents, but differs at all amino acid positions between these two groups (Fig. 5). The same region is also highly variable in other species; the sequences of other vertebrates share little similarity with either the primate or the rodent sequence in the cluster region (Fig. 5).

**Figure 6:**
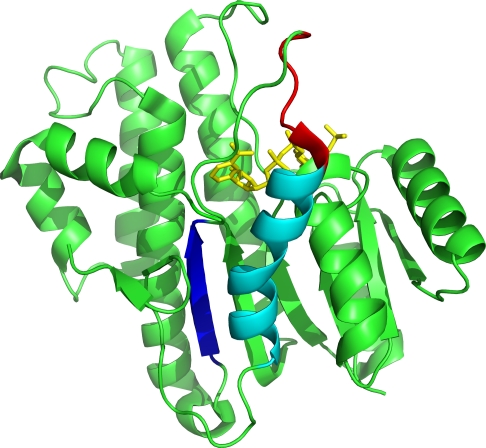
The tertiary structure of CBR3. The mutation cluster is shown in red, *β*-sheet F is shown in blue, and *α*-helix G is shown in cyan, while the remainder of the protein is shown in green. Coenzyme NADPH is shown in yellow.

The tertiary structure of CBR3 is shown in Fig. 6. In this protein, it is known that the peptide region in the C-terminal half constitutes the outer walls of the substrate-binding cleft of the active site, which provides specific interactions that are critical to the selectivity of substrates and to the mechanism of molecular recognition by the enzyme [52]. The mutation cluster detected by our method is located in the substrate entry-loop between *β*-sheet F (*β*F) and *α*-helix G (*α*G), which tightens on the substrate upon its entry into the active site providing additional substrate-specific interactions [52]–[55]. It is also reported that this entry-loop is tighter in human CBR3 in comparison with porcine testicular carbonyl reductase (PTCR), which shares about 70% sequence identity with CBR3 [56]. Conceivably, the mutation cluster influences the docking and/or release of the cofactor during enzymatic catalysis [56].

**Figure 7:**
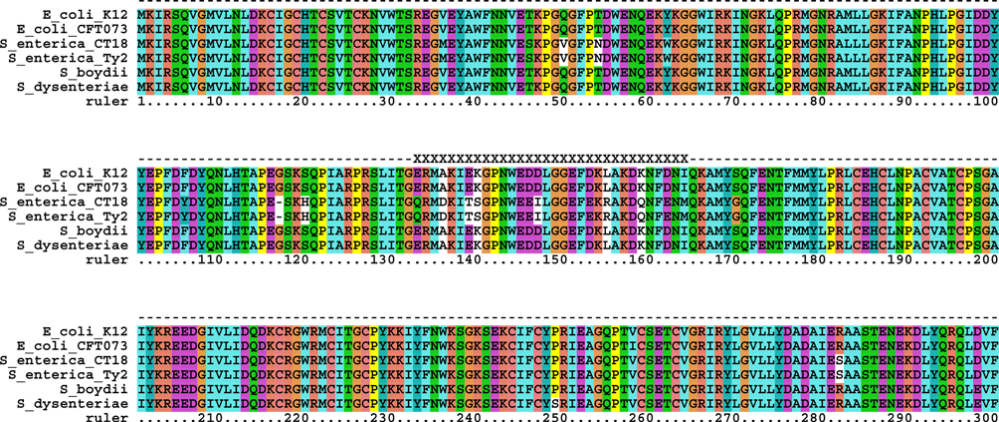
Multiple sequence alignment of the *E. coli* protein NarH and its orthologs in *S. enterica*, *Shigella boydii* and *Shigella dysenteriae*. The mutation cluster spans positions 133 to 164 and is marked by the symbol X.

The second example is the *β* subunit of nitrate reductase A (NarH, b1225) in *E. coli*. The mutation cluster runs from position 133 to position 164, and is completely conserved within both *E. coli* and *S. enterica* (Fig. 7). Among the two groups, the cluster region has 66% sequence similarity, while the entire gene has 93% sequence similarity. *Shigella* sequences in the cluster region are identical to the *E. coli* sequences (Fig. 7), in agreement with the notion that *Shigella* strains are clones of *E. coli* [57].

**Figure 8:**
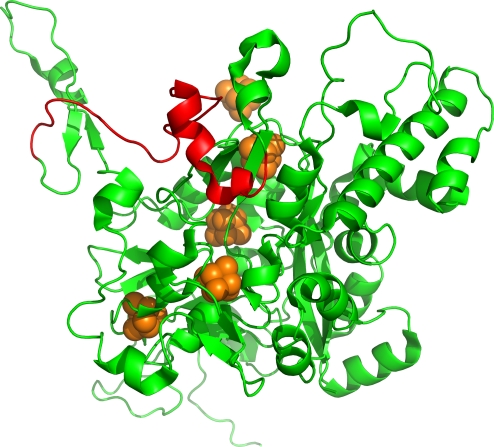
The tertiary structure of NarH. The mutation cluster is shown in red, while the remainder of the protein is shown in green. [Fe-S] clusters are shown in orange.

*E. coli* can use nitrate as an electron acceptor for anaerobic growth [58], [59]. This oxidoreduction is catalyzed by nitrate reductase A (NarGHI), which is a membrane-bound complex of three subunits coded by three genes, NarG, NarH, and NarJ. NarH is an [Fe-S]-cluster-containing electron transfer subunit [59], [60]. The mutation cluster found in NarH is located in the motif which is thought to have an important function in defining subunit-subunit interactions within the overall structure of NarGHI and to provide additional shielding of [Fe-S] clusters from the aqueous milieu [60] (Fig. 8).

Other clusters that were readily determined to be in locations important to the structure and/or function of the protein are as follows. The cluster in the *E. coli* gene *pepN* lies in the substrate-recognition domain of the protein [61]. The cluster in the fly gene *CkIIβ2* partly overlaps with an acidic loop that is important for modulating autophosphorylation and the overall activity of the protein [62]. The cluster in the mammalian gene *NEDD8* is completely conserved in all vertebrates but rodents. Non-rodent vertebrates have a lysine at position four which forms a salt bridge with the glutamic acid at position 12 [63]. In rodents, the lysine is replaced by another glutamic acid, which disrupts the salt bridge and likely alters the protein structure.

### Comparison with dN/dS-based methods

As discussed in the introduction, the most commonly used methods to detect positive selection rely on high *dN*/*dS* values. Therefore, for genes for which we found clusters, we also carried out *dN*/*dS*-based analyses.

First, for the mammalian clusters, we determined whether our mutation clusters coincided with sites predicted to have elevated *dN*/*dS* according to Bayes Empirical Bayes Inference [9], as published in the Human PAML Browser [64] (http://mendel.gene.cwru.edu/adamslab/pbrowser.py). Of the five genes for which we identified mutation clusters, the PAML Browser contained results for only three (Ensembl IDs ENSG00000105220, ENSG00000129559, ENSG00000198951). For neither of these did we find that mutation clusters overlapped with sites predicted to have *dN*/*dS*.

**Figure 9:**
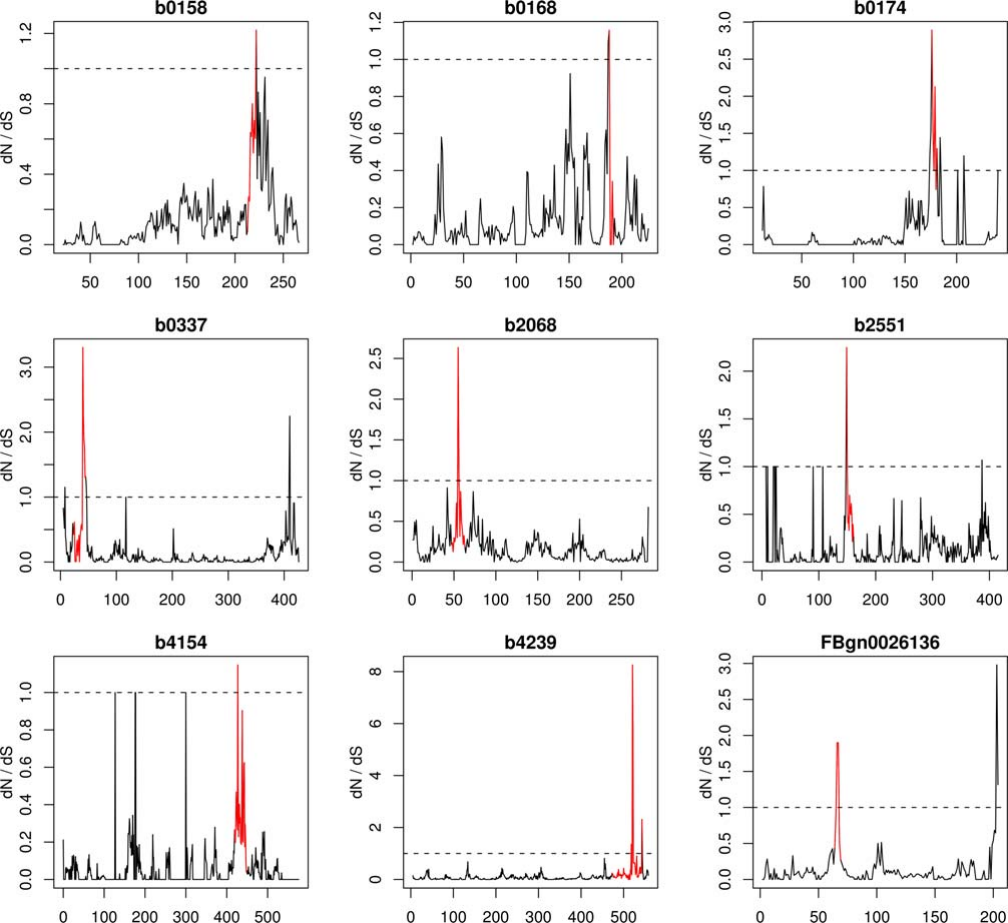
*dN*/*dS* in 3D window versus the residue number at the center of the 3D window. The red coloration indicates residues which we identified as being part of a mutation cluster. The dashed line indicates *dN*/*dS* = 1. We show 3D-window analyses for all cases in which a mutation cluster we identified coincided with a *dN*/*dS*-value above 1. There are eight such cases for bacteria (b0158–b4239), one in fly (FBgn0026136), and none in mammals.

Second, we compared our results to results obtained by a 3D sliding window method [7], [65], [66]. We carried out this analysis using the SWAKK web server [66] (http://oxytricha.princeton.edu/SWAKK/), using a 3D window size of 10Å. Because this method can only work on pairs of sequences, we compared *E. coli* K12 with *S. enterica* CT18 for bacteria, *D. melanogaster* with *D. persimilis* for fly, and human with mouse for mammals. As with Bayes Empirical Bayes Inference, the mammalian clusters did not overlap with regions predicted to have *dN*/*dS*>1. In contrast, eight of the bacterial clusters and one fly cluster coincided with regions with *dN*/*dS*>1 (Fig. 9).

## Discussion

We have presented a new method to discover clustered evolution in protein-coding sequences. Our method takes into account the increased variability of exposed residues relative to buried residues and finds clusters of mutations that are unlikely to have arisen by chance given their composition of buried and exposed residues. The method can find multiple clusters in a single gene, and uses permutation tests to assign accurate *P*-values to each cluster.

We have used this method to search for mutation clusters in bacteria, fly, and mammals. By and large, we have found that mutation clusters are not particularly common. We found a total of 31 clusters in 356 bacterial genes, 6 clusters in 99 fly genes, and 5 clusters in 246 mammalian genes. However, some of the clusters we found were striking. For example, in the *E. coli* fumarate reductase flavoprotein subunit (FrdA, b4154), nearly half of the sequence differences relative to the *S. enterica* ortholog fall into the mutation cluster, which nonetheless spans only 5% of the entire protein. Several of the clusters we have identified seem to be located in or adjacent to active sites or otherwise functionally relevant regions of the protein. We therefore expect that a good fraction of the clusters we found reflect functional divergence between the species groups we compared. Unfortunately, we did not find a single example where the corresponding protein had been experimentally characterized in both species. Therefore, at this point we can only speculate about the meaning of the clusters.

By controlling for solvent accessibility, we avoid detecting spurious clusters that only reflect inherent variability differences along the protein sequence. Yet controlling for solvent accessibility does not preclude the possibility that clusters arise more frequently in either buried or exposed regions. For example, if mutations in exposed regions tended to be distributed uniformly along the protein sequence whereas mutations in buried regions tended to be clustered together, we would find an excess of mutation clusters in buried regions even after controlling for solvent accessibility. We therefore tested whether mutation clusters were particularly likely to appear in either buried or exposed regions, and found no such signal. Neither did we find a propensity of clusters to appear in specific secondary structure elements.

We also tested whether mutations in clusters were more physicochemically radical. We found a weak signal for molecular volume, but no signal for side-chain composition, polarity, hydropathy, or isoelectric point. This result seems to support the notion that adaptive evolution does not coincide with more radical amino-acid replacements [39]–[41]. Because we had a relatively small data set of only 42 significant mutation clusters, we had limited statistical power to detect differences between mutations inside and outside of clusters. Therefore, our results do not imply that clustered mutations are completely indistinguishable from non-clustered mutations. However, they do imply that any difference between these two types of mutations must be minor.

We have found that controlling for solvent accessibility is crucial to avoid detecting clusters that simply reflect highly variable loop regions or other highly variable exposed regions of the protein. If we do not control for solvent accessibility, we find an excess of mutation clusters in highly variable terminal regions of the proteins, and we find clusters that are predominantly comprised of solvent-exposed residues.

One issue we encountered repeatedly when we initiated this work was the emergence of spurious clusters in two-species comparisons. The most extreme case arose in a comparison of human to macaque genes, where we found multiple putative clusters that could be traced to problems with the macaque draft genome sequence. To avoid such problems, we decided to base our analysis on a comparison of pairs of species, and considered as mutated only those sites that were conserved within each pair but differed among pairs. Conversely, our method might be useful for quality control in the automated assembly of newly sequenced species. Any cluster that shows up in a pairwise comparison of a newly sequenced species and a closely related species should be considered suspicious. These clusters could then be double-checked manually for accuracy.

Our method has several limitations. First, we require solved protein structures for every gene we analyze. This requirement severely limits the size of the data sets we can analyze. One possibility to alleviate this limitation would be to use computationally predicted solvent accessibilities for those genes or parts of genes for which no solved protein structure is available. The drawback of this approach is that these computational predictions are typically only 70%–80% accurate [67]–[71], and it is not clear how incorrectly predicted solvent accessibility would affect the clusters found by our algorithm.

Second, and more importantly, our method finds clusters in the protein's primary structure (i.e., its sequence). Mutation clusters in the primary structure will generally map to clusters in the tertiary structure [10], but the converse is not necessarily true. A cluster in tertiary structure can conceivably consist of mutations that are distant in primary structure. Such clusters would be missed by our method. It would, however, be straightforward to modify our method so that it does apply to 3D space. Instead of searching for clusters in consecutive stretches of the amino-acid sequence, we would have to consider spheres with varying radii centered around the mutations in the protein. Eq. 3 would still apply if we interpreted *n_b_* and *n_e_* as the total number of buried and exposed amino acids in the sphere, and *f_b_* and *f_e_* as the fraction of mutations at buried and exposed sites in the protein. All other aspects of our method would transfer to the 3D case without change.

Third, separating all residues into groups of either buried or exposed residues may be too coarse. Sequence conservation varies continuously with solvent accessibility [31], and hence there may still be significant variation in the mutation probabilities within all residues we considered exposed or buried. Moreover, we considered only the solvent accessibility in a protein's tertiary structure. However, residues that are solvent-exposed in tertiary structure but buried in quaternary structure tend to be more conserved than residues that remain always exposed [35], [72], [73]. In principle, all these drawbacks can be alleviated by introducing additional classes of residues, say partially exposed residues, or exposed residues in contact with other proteins. The problem with such an approach is that with any additional residue class that we introduce, it becomes harder to reliably estimate the mutation frequency within that class. One possibility would be to combine our approach with evolutionary trace methods [74]. Evolutionary trace methods aim to identify functional sites in proteins by locating regions of high sequence conservation in large multiple sequence alignments, whereas our approach does the opposite. It finds regions with unusually high sequence variability. It would be possible to use a method similar to the evolutionary trace to calculate a background variability of each site, and then use a method similar to ours to search for clusters of mutations that are particularly unlikely to arise under this background level of variation.

When comparing mutation clusters to results from *dN*/*dS*-based methods, we found that approximately 20% of the clusters we identified coincided with regions with *dN*/*dS*>1, while the remainder did not. What should we have expected for this comparison? One significant difference between mutation clusters and the *dN*/*dS*-based methods is that the latter use an absolute standard, i.e., they search for sites or regions with *dN*/*dS*>1, whereas our method finds regions in which *dN* is elevated compared to the rest of the gene. For example, if a gene has *dN*/*dS* = 0.01 throughout, apart from a small region with *dN*/*dS* = 0.8, and assuming that the difference in *dN*/*dS* is caused by a change in *dN* and not *dS*, the region with *dN*/*dS* = 0.8 would likely be identified as a mutation cluster by our method but would not register in screens for *dN*/*dS*>1. On the other hand, because we identify mutation clusters purely based on nonsynonymous mutations, post-hoc testing for elevated *dN*/*dS* in cluster regions suffers from ascertainment bias. In other words, we expect to find cases with *dN*/*dS*>1 simply because of the way in which we carried out our analysis, and we would obtain this result even in simulated data sets generated with completely homogeneous substitution rates and without any positive selection.

Do the clusters we identify actually represent positive selection, or might they just reflect relaxed selective pressures? We concede that the latter is a realistic possibility. Even though we certainly removed some regions of relaxed selection by considering separately the more and less variable regions in each protein, we have no guarantee that the remaining clusters are not caused by relaxed selection. In fact, positive and relaxed selection can lead to very similar patterns of evolution. For instance, significant divergence in the active site of a protein could indicate adaptation to a new enzymatic function, but it could also indicate loss of function. An example of the latter case would be a protein whose main importance has become structural, as has happened with crystallins [75]. As recent work on the *dN*/*dS* method has shown [23], reliable identification of positive selection by purely statistical methods is extremely difficult. For these reasons, we believe that the main purpose of our method is to identify unusual patterns of sequence divergence. The mechanisms by which these patterns arose will have to be determined separately, most likely by direct biochemical experimentation.

## Materials and Methods

### Genomic and structural data

For bacteria, we obtained orthologs between *E. coli K12*, *E. coli CFT073*, *S. enterica CT18*, and *S. enterica Ty2* from TIGR's Comprehensive Microbial Resource's multi-genome homology comparison tool (http://cmr.tigr.org/). For fly, we obtained orthologs between *D. melanogaster*, *D. sechellia*, *D. persimilis*, and *D. pseudoobscura* from the *Drosophila* 12-genome project AAAWiki at http://rana.lbl.gov/drosophila/. For mammals, we obtained orthologs between *H. sapiens*, *P. troglodytes*, *M. musculus*, and *R. norvegicus* from Biomart through the Ensembl Homology track (http://www.ensembl.org/). For each group of orthologs, we obtained aligned nucleotide sequences based on the alignment of the peptide sequences, which we generated with MUSCLE [76]. We excluded from our data set those ortholog pairs for which less than 80% of either sequence could be aligned to the other sequence. Then we determined from the alignments the number and coordinates of all amino acid changes that had occurred between the species pairs of each group (bacteria, fly, and mammals). In other words, we considered only mutations shared by the species pairs. We excluded from this count all sites at which at least one sequence had an indel (alignment gap). For genes with multiple transcripts, we based our analysis on the longest transcript that could be aligned to a PDB structure (see next paragraph). Moreover, to be conservative, we considered only those sites as mutated for which no transcript in one species pair agreed with any transcript in the other species pair.

We matched sequences to protein structures using the GTOP (Genomes TO Protein structures and functions) database [77]. For a given match in the GTOP database, if the region of similarity was longer than 80% of the protein length and the sequence identity was larger than 40% of the sequence in the Protein Data Bank (PDB), the match was saved for further calculation. This process yielded 777, 795, and 860 matches in *E. coli*, *D. melanogaster*, and *H. sapiens*, respectively.

For each protein with a match, the corresponding 3D structural information was obtained from the PDB. We aligned the orthologs plus the sequence of the corresponding PDB structure with MUSCLE, and then calculated the percent solvent-accessible surface area for each orthologous residue position using the DSSP (Dictionary of Protein Secondary Structure) program [78]. We normalized these results by the reference surface areas of an extended Gly-X-Gly peptide [79]. We considered residues with less than 25% relative solvent accessibility as buried. We also calculated the secondary structure for each aligned residue position using the DSSP program [78]. We simplified our data set by keeping track of only four types of secondary structure elements: helix (DSSP class H), sheet (DSSP class E), turn (DSSP classes S and T), and coil (DSSP classes B, G, I, and ‘.’).

We excluded from our analysis those alignments in which there was at least one site without known solvent accessibility. Our final datasets contained 356, 99, and 246 orthologs for bacteria, fly, and mammals, respectively.

### Computational method for cluster detection

Under neutrality, all sites in a protein of length *l* with *m* amino acid mutations are equally likely to have been mutated. Therefore, the *m* mutations should be evenly distributed over the entire protein. In this case, the probability of getting exactly *k* mutations in *n* successive residues is given by the binomial distribution,
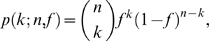
(1)where 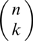 is the binomial coefficient, and we define *f* = *m*/*l*. The probability *q*(*k*; *n*, *f*) that the number of mutations in *n* successive residues is no less than *k* is equal to
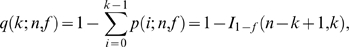
(2)where *I*_1−*f*_ (*n*−*k*+1, *k*) is the regularized incomplete beta function [80].

Now assume that the protein is subdivided into buried and exposed residues, and that the mutation probability differs among these two classes of residues. We denote the number of exposed residues by *l_e_* and the number of buried residues by *l_b_*, with *l_e_*+*l_b_* = *l*. Assume that there are *m_e_* mutations at exposed sites and *m_b_* mutations at buried sites, with *m_e_*+*m_b_* = *m*. Then, for a stretch of *n* residues with *n_e_* exposed residues and *n_b_* buried residues (*n* = *n_e_*+*n_b_*), the probability that these *n* residues contain at least *k* mutations, given that mutations are equally likely at all exposed and all buried sites, becomes
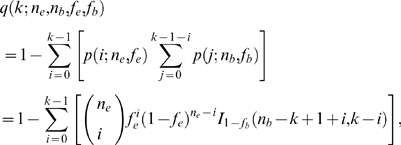
(3)where *f_e_* = *m_e_*/*l_e_* and *f_b_* = *m_b_*/*l_b_*.

Using Eq. 3, we calculate *q*(*k*; *n_e_*, *n_b_*, *f_e_*, *f_b_*) for all possible contiguous sets of mutations. Assume that the m mutations are located at positions *x*_1_, *x*_2_, …, *x_m_* in the gene. We first consider all possible clusters starting with the first mutation at position *x*_1_. The corresponding sets of mutations are {*x*_1_, *x*_2_}, {*x*_1_, *x*_2_, *x*_3_}, …, {*x*_1_, …, *x_m_*}. The set with the minimum *q*(*k*; *n_e_*, *n_b_*, *f_e_*, *f_b_*) is recorded as *C*_1_, and the corresponding *q*(*k*; *n_e_*, *n_b_*, *f_e_*, *f_b_*) as *Q*_1_. We then repeat this procedure for sets starting at position *x*_2_, *x*_3_, and so on. This procedure yields mutation sets *C*_2_, *C*_3_, …, *C_m_*_−1_ with associated minimum-*q*(*k*; *n_e_*, *n_b_*, *f_e_*, *f_b_*) values *Q*_2_, *Q*_3_, …, *Q_m_*_−1_. The *Q*-landscape plots the *Q_i_* values (*i* = 1, 2, …, *m*−1) against the corresponding mutation index *i* (see Fig. 1). Local minima in this landscape represent possible mutation clusters, and we discard all sets of mutations that overlap with other sets having lower *Q* values.

*Q* values are probabilities, but they do not correspond to the probability that a given cluster arises by chance in the context of the other mutations present in the gene. In other words, we cannot equate a cluster's *Q* value with the cluster's *P* value. We calculate *P* values by interpreting *Q* as our test statistic. (In this context, we add the subscript *s* to *Q*.) For a given cluster with test statistic *Q_s_* in a given gene, we carry out at least 10^4^ independent, random reshufflings of the mutations, keeping the number of mutations at buried and exposed sites constant. For each reshuffled set of mutations, we repeat our procedure of identifying non-overlapping sets of mutations starting at local minima in the *Q*-landscape, and record whether *Q_s_*<*Q* for these sets. The total fraction of times that *Q_s_*<*Q* is the cluster's *P* value. We refer to this value as *P_U_*, because it has not been corrected for multiple testing. We then carry out a false-discovery-rate correction [36] on the *P_U_* values for all potential clusters in a given species, and record the corrected values as *P_M_*. Clusters with *P_M_*<0.05 are significant and are unlikely to have arisen by chance.

We implemented this algorithm as a C program called “ClusterExplorer”. The program's source code is available as part of the online supplementary materials for this paper.

## Supporting Information

Text S1 Summary of supplementary materials.
(0.02 MB PDF)

Table S1 Significant mutation clusters in bacteria.
(0.03 MB PDF)

Table S2 Significant mutation clusters in fly.
(0.03 MB PDF)

Table S3 Significant mutation clusters in mammals.
(0.03 MB PDF)

Figures S1 Supporting figures S1.1–S1.39.
(5.48 MB PDF)
