# Supplementary material for: Detecting Clusters of Mutations
Source: PLoS One. 2008 Nov 19;3(11):e3765. doi: 10.1371/journal.pone.0003765 (PMC2582452; doi:10.1371/journal.pone.0003765)
Supplement: Text S1 — Summary of supplementary materials. (0.02 MB PDF) [file pone.0003765.s001.pdf]

# Supplementary text for
# Zhou et al., Detecting clusters of mutations

We found a total of 31, 6, and 5 significant mutation clusters in the bacteria, fly, and mammal groups (see Tables S1, S2, and S3).

For the bacteria group, we aligned the sequences in this group to their corresponding orthologs in *Shigella boydii Sb227*, *Shigella dysenteriae Sd197*, *Photorhabdus luminescens TTO1*, *Yersinia pestis CO92*, *Buchnera aphidicola* (*Baizongia pistaciae*), *Acidithiobacillus ferrooxidans ATCC 23270*, and *Legionella pneumophila Lens*, if available.

For the fly group, we aligned the sequences in *D. melanogaster*, *D. sechellia*, *D. persimilis* and *D. pseudoobscura* to their corresponding orthologs in *D. simulans*, *D. yakuba*, *D. erecta*, *D. ananassae*, *D. willistoni*, *D. mojavensis*, *D. virilis*, and *D. grimshawi*.

For the mammal group, we aligned the sequences in this group to their corresponding orthologs in macaque (*Macaca mulatta*), cow (*Bos taurus*), cat (*Felis catus*), chicken (*Gallus gallus*), zebrafish (*Danio rerio*) and *Xenopus tropicalis*.

In all these multiple sequence alignments, the regions of mutation clusters are marked with the symbol X (Figs. S1.1-S1.39). Note that for long genes, only the part of the sequence alignment containing the cluster is shown.

Figs. S1.1-S1.39 also show the tertiary structures of the corresponding proteins, with the mutation clusters indicated by red coloration.
