# Supplementary material for: Detecting Clusters of Mutations
Source: PLoS One. 2008 Nov 19;3(11):e3765. doi: 10.1371/journal.pone.0003765 (PMC2582452; doi:10.1371/journal.pone.0003765)
Supplement: Table S1 — Significant mutation clusters in bacteria. (0.03 MB PDF) [file pone.0003765.s002.pdf]

Table S1: Significant mutation clusters in bacteria (*E. coli* vs *S. enterica*). All clusters remain significant after correction for multiple testing and have $P_M < 0.05$.

E. coli K12 gene	*E. coli CFT073* gene	*S. enterica CT18* gene	*S. enterica Ty2* gene	PDB ID	Chain	Start	End	f_{bur}	Q_s	P_U
b0052	c_0065	STY0106	t0094	1ps6	A	2	95	0.53	8.25×10^{-6}	1.65×10^{-4}
b0118	c_0147	STY0181	t0164	1l5j	A	75	82	0.50	2.31×10^{-6}	1.88×10^{-4}
b0158	c_0194	STY0228	t0207	1n2z	A	213	222	0.50	1.01×10^{-5}	2.15×10^{-4}
b0168	c_0203	STY0238	t0216	2gg2	A	188	192	0.40	1.80×10^{-5}	4.68×10^{-4}
b0174	c_0211	STY0244	t0222	1x06	A	176	181	0.67	4.34×10^{-5}	1.05×10^{-3}
b0337	c_0456	STY3514	t3251	1ra0	A	23	45	0.22	2.03×10^{-7}	3.59×10^{-6}
b0459	c_0577	STY0515	t2388	1ocx	A	34	59	0.35	4.47×10^{-6}	3.26×10^{-5}
b0776	c_0856	STY0828	t2092	1bs0	A	192	224	0.67	3.23×10^{-5}	8.74×10^{-4}
b0932	c_1075	STY1078	t1863	2dq6	A	622	706	0.55	1.22×10^{-7}	1.11×10^{-5}
b1096	c_1366	STY1237	t1722	1i2k	A	31	76	0.50	3.14×10^{-5}	5.15×10^{-4}
b1100	c_1372	STY1241	t1718	1yix	A	72	100	0.59	3.69×10^{-5}	8.43×10^{-4}
b1225	c_1686	STY1289	t1672	1q16	B	133	164	0.38	1.32×10^{-5}	6.82×10^{-4}
b1264	c_1730	STY1328	t1635	1i1q	A	132	251	0.51	9.38×10^{-6}	3.76×10^{-4}
b1732	c_2131	STY1793	t1198	1qf7	A	518	750	0.55	8.44×10^{-7}	4.63×10^{-5}
b1804	c_2208	STY1947	t1060	1yt3	A	312	325	0.36	1.59×10^{-5}	3.79×10^{-4}
b1960	c_2379	STY2199	t0887	1cw0	A	30	58	0.48	1.07×10^{-6}	1.94×10^{-6}
b2068	c_2595	STY2337	t0748	1mpg	A	47	63	0.59	8.57×10^{-7}	9.89×10^{-6}
b2500	c_3018	STY2741	t0357	1jkx	A	52	77	0.38	6.54×10^{-5}	1.03×10^{-3}
b2551	c_3073	STY2802	t0301	1eqb	B	149	161	0.46	4.95×10^{-7}	2.07×10^{-5}
b2745	c_3312	STY3053	t2829	1si7	A	138	154	0.35	3.05×10^{-5}	1.05×10^{-3}
b2822	c_3416	STY3134	t2904	1w36	C	279	285	0.57	6.09×10^{-6}	3.45×10^{-4}
b2822	c_3416	STY3134	t2904	1w36	C	1098	1112	0.33	1.56×10^{-5}	9.18×10^{-4}
b2838	c_3435	STY3157	t2923	1knw	A	2	9	0.25	1.39×10^{-7}	5.72×10^{-6}
b3417	c_4194	STY4282	t3992	1ahp	A	4	57	0.46	2.47×10^{-5}	1.20×10^{-3}
b3634	c_4458	STY4069	t3793	1qjc	A	80	85	0.50	1.13×10^{-005}	1.77×10^{-4}
b3656	c_4489	STY4044	t3770	2f2h	A	90	113	0.58	1.20×10^{-7}	9.14×10^{-6}
b3656	c_4489	STY4044	t3770	2f2h	A	742	748	0.43	1.68×10^{-5}	7.38×10^{-4}
b4039	c_5009	STY4429	t4139	1tt8	A	53	65	0.38	5.65×10^{-5}	7.84×10^{-4}
b4154	c_5242	STY4703	t4395	1kf6	A	419	449	0.29	1.35×10^{-8}	9.56×10^{-7}
b4239	c_5338	STY4793	t4488	1uok	-	473	543	0.54	4.67×10^{-6}	1.61×10^{-4}
b4378	c_5461	STY4915	t4607	1zzm	A	54	121	0.56	3.47×10^{-5}	6.39×10^{-4}

f_{bur}: fraction of buried sites. We determined the start and end coordinates of mutation clusters according to the PDB code and its corresponding sequence.
