# Supplementary material for: Detecting Clusters of Mutations
Source: PLoS One. 2008 Nov 19;3(11):e3765. doi: 10.1371/journal.pone.0003765 (PMC2582452; doi:10.1371/journal.pone.0003765)
Supplement: Table S2 — Significant mutation clusters in fly. (0.03 MB PDF) [file pone.0003765.s003.pdf]

Table S2: Significant mutation clusters in fly (*D. melanogaster* vs *D. obscura*). All clusters remain significant after correction for multiple testing and have $P_M < 0.05$.

D. melanogaster gene	*D. sechellia* gene	*D. persimilis* gene	*D. pseudoobscura* gene	PDB ID	Chain	Start	End	f_{bur}	Q_s	P_U
FBgn0004003	dsec_GLEANR_4690	dper_GLEANR_19431	dpse_GLEANR_11995	1ovn	B	187	196	0.70	4.26×10^{-5}	6.73×10^{-4}
FBgn0010590	dsec_GLEANR_2919	dper_GLEANR_10269	dpse_GLEANR_13784	1iru	H	143	158	0.44	9.44×10^{-6}	1.56×10^{-4}
FBgn0011740	dsec_GLEANR_6829	dper_GLEANR_14284	dpse_GLEANR_4516	1qx1	A	446	460	0.40	8.47×10^{-7}	3.90×10^{-5}
FBgn0011740	dsec_GLEANR_6829	dper_GLEANR_14284	dpse_GLEANR_4516	1qx1	A	738	743	0.50	2.11×10^{-5}	9.89×10^{-4}
FBgn0023175	dsec_GLEANR_739	dper_GLEANR_10298	dpse_GLEANR_13815	1iru	G	179	241	0.54	2.96×10^{-5}	7.09×10^{-4}
FBgn0026136	dsec_GLEANR_4754	dper_GLEANR_19424	dpse_GLEANR_15760	1jwh	D	64	71	0.13	2.56×10^{-5}	3.31×10^{-4}

f_{bur}: fraction of buried sites. The genes in *D. melanogaster* are represented by Flybase gene ID. The genes in *D. sechellia*, *D. persimilis* gene and *D. pseudoobscura* are represented by GLEANR gene ID. We determined the start and end coordinates of mutation clusters according to the PDB code and its corresponding sequence.
