# Supplementary material for: Detecting Clusters of Mutations
Source: PLoS One. 2008 Nov 19;3(11):e3765. doi: 10.1371/journal.pone.0003765 (PMC2582452; doi:10.1371/journal.pone.0003765)
Supplement: Table S3 — Significant mutation clusters in mammals. (0.03 MB PDF) [file pone.0003765.s004.pdf]

Table S3: Significant mutation clusters in mammals (primates vs rodents). All clusters remain significant after correction for multiple testing and have $P_M < 0.05$.

Human gene	Chimpanzee gene	Mouse gene	Rat gene	PDB ID	Chain	Start	End	f_{bur}	Q_s	P_U
ENSG00000105220	ENSPTRG00000010816	ENSMUSG00000036427	ENSRNOG00000023150	2cxn	A	14	35	0.36	1.94×10^{-8}	9.44×10^{-7}
ENSG00000129559	ENSPTRG00000006206	ENSMUSG00000010376	ENSRNOG00000019895	1bt0	A	2	5	0.50	1.01×10^{-5}	8.21×10^{-5}
ENSG00000159231	ENSPTRG00000032586	ENSMUSG00000022947	ENSRNOG00000001701	2hrb	A	236	241	0.33	9.31×10^{-6}	2.21×10^{-4}
ENSG00000167419	ENSPTRG00000009448	ENSMUSG00000009356	ENSRNOG00000008422	2gjm	A	208	216	0.67	6.36×10^{-6}	2.62×10^{-4}
ENSG00000198951	ENSPTRG00000014446	ENSMUSG00000022453	ENSRNOG00000008064	1ktb	A	335	357	0.57	1.06×10^{-7}	8.00×10^{-6}

f_{bur}: fraction of buried sites. Each gene is represented by the Ensembl gene ID. We determined the start and end coordinates of mutation clusters according to the PDB code and its corresponding sequence.
