# Supplementary material for: Detecting Clusters of Mutations
Source: PLoS One. 2008 Nov 19;3(11):e3765. doi: 10.1371/journal.pone.0003765 (PMC2582452; doi:10.1371/journal.pone.0003765)

Figure S1.1: Sequence alignment and tertiary structure for gene *pdxA*. The mutation cluster is highlighted in red.

E. coli K12 entry: b0052; PDB ID: 1ps6; chain ID: A

```
                  ---------XXXXXXXXXXXXXXXXXXXXXXXXXXXXXXXXXXXXXXXXXXXXXXXXXXXXXXXXXXXXXXXXXXXXXXXXXXXXXXXXXXX
       E_coli_K12 ---------MVKTQRVVITPGEPAGIGPDLVVQLAQREWPVELVVCADATLLTNRAAMLGLPLTLRPYSPNSPAQPQTAGTLTLLPVALRAPVTAGQLAV
    E_coli_CFT073 ---------MVKTQRVVITPGEPAGIGPDLIVQLAQREWPVELVVCADATLLTDRAAMLGLPLTLRPYSPNSPAQPQTAGTLTLLPVALRESVTVGQLAV
S_enterica_CT18 ---------MSSAQRVVITPGEPAGIGPDLVVQLAQRAWPIELVVCADGALLTERAAMLGLPLSLLPYSPDVPAAPQPAGTLTLLPVSLRAPAIPGQLTV
  S_enterica_Ty2 ---------MSSAQRVVITPGEPAGIGPDLVVQLAQRAWPIELVVCADGALLTERAAMLGLPLSLLPYSPDVPAAPQPAGTLTLLPVSLRAPAIPGQLTV
        S_boydii ---------MVKTQRVVITPGEPAGIGPDLVVQLAQREWPVELVVCADATLLTDRAAMLGLPLTLRPYSPNSPAQPQTAGTLTLLPVALRESVTAGQLAI
   S_dysenteriae ---------MVKTQRVVITPGEPAGIGPDLVVQLAQREWPVELVVCADATLLTDRAAMLGLPLTLRPYSPNSPAQPQTAGILTLLPVALRESVTAGQLAV
   P_luminescens MATMHNNKPIIINKPIVITPGEPAGVGPDLTVALAQQSWPMQLVACADPNLLLDRARQLNLPLQLQEYSADKTPEPQAAGTLTILPVPLHVPAIAGELNQ
        Y_pestis --------MHNHNNRLVITPGEPAGVGPDLAITLAQQDWPVELVVCADPALLLARASQLNLPLQLREYQADQPAIAQQAGSLTILPVKTAVNVVPGKLDV
  A_ferrooxidans ---------MITEPRLLLTVGEPAGIGPDICLQLAFHALPSGVLLIGDLHCLRSRALTLGLSLRLEPWLEGNPWPALERGVLHVLDVPLAQPCRPGRLDM
   L_pneumophila -----------MNPLLISSGEPAGIGPDLCLALAETDLP--VVILGDLSLLEARASELNLSIKFIEYSSHQSFE-KKAGYLTVWPVPCAEPVISGELNP
           ruler 1.......10........20........30........40........50........60........70........80........90.......100

                  XXXX------------------------------------------------------------------------------------------------
       E_coli_K12 ENGHYVVETLARACDGCLNGEFAALITGPVHKGVINDAGIPFTGHTEFFEERSQAKKVVMMLATEELRVALATTHLPLRDIADAITPALLHEVIAILHHD
    E_coli_CFT073 ENGHYVVETLARACDGCLNGEFAALITGPVHKGVINDAGIPFTGHTEFFEERSQAKKVVMMLATEELRVALATTHLPLRDIADAITPALLHEVIAILHHD
S_enterica_CT18 ENGPYVVETLARACDGCLNGEFAALITGPVHKGVINDAGIPFTGHTEFFEERSQAKKVVMMLATEELRVALATTHLPLRAIADAITPALLHEVIAILHHD
  S_enterica_Ty2 ENGPYVVETLARACDGCLNGEFAALITGPVHKGVINDAGIPFTGHTEFFEERSQAKKVVMMLATEELRVALATTHLPLRAIADAITPALLHEVIAILHHD
        S_boydii ENGHYVVETLARACDGCLNGEFAALITGPVHKGVINDAGIPFTGHTEFFEERSQAKKVVMMLATEELRVALATTHLPLRDIADAITPALLHEVIAILHHD
   S_dysenteriae ENGHYVVETLARACDGCLNGEFAALITGPVHKGVINDAGIPFTGHTEFFEERSRAKKVVMMLATEELRVALATTHLPLRDIADAITPALLHEVIAILHHD
   P_luminescens ENGRYVTETLAQACDGCLNGEFSALVTGPVHKGNINDAGVPFIGHTEFFADRSGCQRVVMMLATEELRVALATTHLPIVDVPKSITFDSLREVITILNHD
        Y_pestis GNSHYVVETLAKACDGAISGEFAALVTGPVQKSIINDAGIPFIGHTEFFADRSHCQRVVMMLATEELRVALATTHLPLLAVPGAITQASLHEVITILDND
  A_ferrooxidans ANAPAVLATLDKAMHLLRAGAADALVTAPVHKGIINDAGIPFTGHTEYLAAACGSPKVVMLLAGRGLRVALATTHLPLAQVAAAVTQEGLEGTLRILHRA
   L_pneumophila RNAAYVMELLTLGASLCSKGEFSALVTAPVHKANINAAGITFTGHTEFFADFFEVETVVMMLACSQMKVALVTTHLPLRMVPDTISSLLIVKVIQQLHHS
           ruler .......110.......120.......130.......140.......150.......160.......170.......180.......190.......200

                  ----------------------------------------------------------------------------------------------------
       E_coli_K12 LRTKFGIAEPRILVCGLNPHAGEGGHMGTEEIDTIIPVLNELRAQGMKLNGPLPADTLFQPKYLDNADAVLAMYHDQGLPVLKYQGFGRGVNITLGLPFI
    E_coli_CFT073 LRTKFGIAEPRILVCGLNPHAGEGGHMGTEEIDTIIPVLNELREQGMKLNGPLPADTLFQPKYLDNADAVLAMYHDQGLPVLKYQGFGRGVNITLGLPFI
S_enterica_CT18 LRTKFGIAEPRILVCGLNPHAGEGGHMGTEEIDTIIPVLDELRAQGMKLNGPLPADTLFQPKYLDNADAVLAMYHDQGLPVLKYQGFGRGVNITLGLPFI
  S_enterica_Ty2 LRTKFGIAEPRILVCGLNPHAGEGGHMGTEEIDTIIPVLDELRAQGMKLNGPLPADTLFQPKYLDNADAVLAMYHDQGLPVLKYQGFGRGVNITLGLPFI
        S_boydii LRTKFGIAEPRILVCGLNPHAGEGGHMGTEEIDTIIPVLDELRAQGMILNGPLPADTLFQPKYLDNADAVLAMYHDQGLPVLKYQGFGRGVNITLGLPFI
   S_dysenteriae LRTKFGIAEPRILVCGLNPHAGEGGHMGTEEIGTIIPVLDELRAQGMKLSGPLPADTLFQPKYLDNADAVLAMYHDQGLPVLKYQGFGRGVNITLGLPFI
   P_luminescens LKTKFGLAKPRIYVCGLNPHAGEGGHMGREEIDVIIPALASLRAEGIVLEGPLPADTLFQPKYLQHADAVLSMYHDQGLPVLKYQGFGRAVNITLGLPFI
        Y_pestis LKTKFGITQPQIYVCGLNPHAGEGGHMGHEEIDTIIPALNTLRQQGINLIGPLPADTLFQPKYLQHADAVLAMYHDQGLPVLKYQGFGRAVNITLGLPFI
  A_ferrooxidans LREDFALSEPRILVAGLNPHAGEGGHLGHEEQDVIAPVIAALQGESLRISGPWPADTLFTPRLLEDADAVLAMYHDQGLPVLKYHAFGEAVNITLGLPIV
   L_pneumophila LKHDFGIQSPKINVAGLNPHAGESGYLGREEIEIITPALNTLKNQGIDVQGPLPADTMFITNHINHCDAYVAMYHDQGLPVLKYAGFNEAVNITLGLPII
           ruler .......210.......220.......230.......240.......250.......260.......270.......280.......290.......300
```

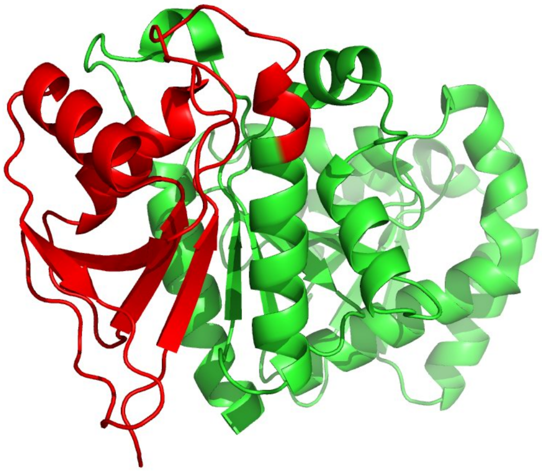

Figure S1.2: Sequence alignment and tertiary structure for gene *acnB*. The mutation cluster is highlighted in red.

E. coli K12 entry: b0118; PDB ID: 1l5j; chain ID: A

```
                 ----------------------------------------------------------------------------------------------------
       E_coli_K12 ---------------------------------------------------VLEEYRKHVAERAAEGIAPKPLDANQMAALVELLKNPPAGEEEFLLDLL
    E_coli_CFT073 MLLFCKQINTSSKSCYSARCGTGHLPYKLLSHRSVKRIACRTMTMRARRTVVLEEYRKHVAERAAEGIAPKPLDANQMAALVELLKNPPAGEEEFLLDLL
  S_enterica_CT18 ---------------------------------------------------VLEEYRKHVAERAAQGIVPKPLDATQMAALVELLKTPPVGEEEFLLDLL
   S_enterica_Ty2 ---------------------------------------------------VLEEYRKHVAERAAQGIVPKPLDATQMAALVELLKTPPVGEEEFLLDLL
         S_boydii ---------------------------------------------------VLEEYRKHVAERAAEGIAPKPLDANQMAALVELLKNPPAGEEEFLLDLL
    S_dysenteriae ---------------------------------------------------VLEEYRKHVAERAAEGIAPKPLDANQMAALVELLKNPPAGEEEFLLDLL
    P_luminescens ---------------------------------------------------VLEEYRKHVAERAAQGVVPKPLDATQAAALVELLKSPPKGEEDFLLDLL
         Y_pestis ---------------------------------------------------VLEEYRKHVAERAAEGIVPKPLDASQMAALVESLKNPPAGEEEFLLDLL
            ruler 1.......10........20........30........40........50........60........70........80........90.......100

                 -------------------------XXXXXXXX-------------------------------------------------------------------
       E_coli_K12 TNRVPPGVDEAAYVKAGFLAAIAKGEAKSPLLTPEKAIELLGTMQGGYNIHPLIDALDDAKLAPIAAKALSHTLLMFDNFYDVEEKAKAGNEYAKQVMQS
    E_coli_CFT073 TNRVPPGVDEAAYVKAGFLAAVAKGEAKSPLLTPEKAIELLGTMQGGYNIHPLIDALDDAKLAPIAAKALSHTLLMFDNFYDVEEKAKAGNEYAKQVMQS
  S_enterica_CT18 INRVPPGVDEAAYVKAGFLAAVAKGDTTSPLVSPEKAIELLGTMQGGYNIHPLIDALDDAKLAPIAAKALSHTLLMFDNFYDVEEKAKAGNEYAKQVMQS
   S_enterica_Ty2 INRVPPGVDEAAYVKAGFLAAVAKGDTTSPLVSPEKAIELLGTMQGGYNIHPLIDALDDAKLAPIAAKALSHTLLMFDNFYDVEEKAKAGNEYAKQVMQS
         S_boydii TNRVPPGVDEAAYVKAGFLAAIAKGEAKSPLLTPEKAIELLGTMQGGYNIHPLIDALDDAKLAPIAAKALSHTLLMFDNFYDVEEKAKAGNEYAKQVMQS
    S_dysenteriae TNRVPPGVDEAAYVKAGFLAAIAKGEAKSPLLTPEKAIELLGTMQGGYNIHPLIDALDDAKLAPIAAKALSHTLLMFDNFYDVEEKAKAGNEYAKQVMQS
    P_luminescens INRIPPGVDEAAYVKAGFLAAIVKDETISPLITPEKAVELLGTMQGGYNIHPLIDALDNEKLAPIAAKALSHTLLMFDNFYDVEEKAKAGNPQAKQIMQS
         Y_pestis TNRVPPGVDEAAYVKAGFLAAIAKGEAHSPLITAEKAIELLGTMQGGYNIHPLIDALDNEQLAPIAAKALSHTLLMFDNFYDVEDKAKAGNPYAKQIIQS
            ruler .......110.......120.......130.......140.......150.......160.......170.......180.......190.......200

                 ----------------------------------------------------------------------------------------------------
       E_coli_K12 WADAEWFLNRPALAEKLTVTVFKVTGETNTDDLSPAPDAWSRPDIPLHALAMLKNAREGIEPDQPGVVGPIKQIEALQQKGFPLAYVGDVVGTGSSRKSA
    E_coli_CFT073 WADAEWFLNRPALAEKLTVTVFKVTGETNTDDLSPAPDAWSRPDIPLHALAMLKNAREGIEPDQPGVVGPIKQIEALQQKGFPLAYVGDVVGTGSSRKSA
  S_enterica_CT18 WADAEWFLSRPPLAEKITVTVFKVTGETNTDDLSPAPDAWSRPDIPLHAQAMLKNAREGIEPDQPGVVGPIKQIEALQKKGYPLAYVGDVVGTGSSRKSA
   S_enterica_Ty2 WADAEWFLSRPPLAEKITVTVFKVTGETNTDDLSPAPDAWSRPDIPLHAQAMLKNAREGIEPDQPGVVGPIKQIEALQKKGYPLAYVGDVVGTGSSRKSA
         S_boydii WADAEWFLNRPALAEKLTVTVFKVTGETNTDDLSPAPDAWSRPDIPLHALAMLKNAREGIEPDQPGVVGPIKQIEALQQKGFPLAYVGDVVGTGSSRKSA
    S_dysenteriae WADAEWFLNRPALAEKLTVTVFKVTGETNTDDLSPAPDAWSRPDIPLHALAMLKNAREGIEPDQPGVVGPIKQIEALQQKGFPLAYVGDVVGTGSSRKSA
    P_luminescens WADAQWYLERPELAEKITVTVFKVTGETNTDDLSPAQDAWSRPDIPLHALAMLKNAREGIVPDQAGVIGPIKQIEELNKKGYPLAYVGDVVGTGSSRKSA
         Y_pestis WADAEWYLSRPALAEKLTVTVFKVTGETNTDDLSPAPDAWSRPDIPLHALAMLKNAREGIHPDQPGSVGPIKQIEELNKKGFPLAYVGDVVGTGSSRKSA
            ruler .......210.......220.......230.......240.......250.......260.......270.......280.......290.......300
```

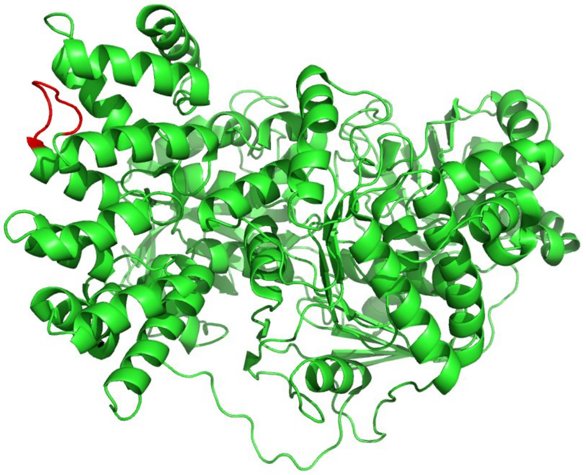

Figure S1.3: Sequence alignment and tertiary structure for gene *btuF*. The mutation cluster is highlighted in red.

E. coli K12 entry: b0158; PDB ID: 1n2z; chain ID: A

```
                ----------------------------------------------------------------------------------------------------
      E_coli_K12 -----MAKSLFRALVALSFLAPLWLNAAP-RVITLSPANTELAFAAGITP--VGVSSYSDYPPQAQKIEQVSTWQGMNLERIVALKPDLVIAWRGGNAER
   E_coli_CFT073 -----MAKSLFRALVALSFLAPLWLNAAP-RVITLSPANTELAFAAGITP--VGVSSYSDYPLQAQKIEQVSTWQGMNLERIVALKPDLVIAWRGGNAER
S_enterica_CT18 -----MAKQMFRALGALLLTLPVWLYAAP-RVITLSPANTELAFAAEITP--VGVSSYSDYPPEAQKIEQVSTWQGMNLERIVALKPDLVVAWRGGNAER
 S_enterica_Ty2 -----MAKQMFRALGALLLTLPVWLYAAP-RVITLSPANTELAFAAEITP--VGVSSYSDYPPEAQKIEQVSTWQGMNLERIVALKPDLVVAWRGGNAER
        S_boydii -----MAKSLFRALVALSFLAPLWLNAAP-RVITLSPANTELAFAAGITP--VGVSSYSDYPPQAQKIEQVSTWQGMNLERIVALKPDLVIAWRGGNAER
   S_dysenteriae -----MAKSLFRALVALSFLAPLWLNAAP-RVITLSPANTELAFAAGITP--VGVSSYSDYPPQAQKIEQVSTWQGMNLERIVALKPDLVIAWRGGNAER
   P_luminescens MKWIKSTGSIGLSLLLFLSSFSHSLYAAPLRVISLSPSTTELAYAAGLGDNLIAASAYSDYPPQARKLEQVANWQGINLERIITLKPELILAWRGGNPQR
        Y_pestis MMPLGLFPLPRAAAVLLISLLTLPAQAAE-RVISLSPSTTELAYAAGLGDKLVAVSAYSDYPESAKKLEHVASWQGINVERILALKPDLILAWRGGNPQR
           ruler 1.......10........20........30........40........50........60........70........80........90.......100

                ----------------------------------------------------------------------------------------------------
      E_coli_K12 QVDQLASLGIKVMWVDATSIEQIANALRQLAPWSPQPDKAEQAAQSLLDQYAQLKAQYADKPKKRVFLQFGINPPFTSGKESIQNQVLEVCGGENIFKDS
   E_coli_CFT073 QVDQLASLGIKVMWVDATSIEQIANALRQLAPWSPQPDKAEQAAQSLLDQYAQLKAQYADKPKKRVFLQFGINPPFTSGKESIQNQVLEVCGGENIFKDS
S_enterica_CT18 QVNQLTSLGIKVMWVDAVTIEQIADALRQLAAWSPQPEKAQQAAQTLLKEYAALKVEYAGKAKKRVFLQFGMNPLFTSGKGSIQHQVLTTCGGENVFADS
 S_enterica_Ty2 QVNQLTSLGIKVMWVDAVTIEQIADALRQLAAWSPQPEKAQQAAQTLLKEYAALKVEYAGKAKKRVFLQFGMNPLFTSGKGSIQHQVLTTCGGENVFADS
        S_boydii QVDQLASPGIKVMWVDATSIEQIANALRQLAPWSPQPDKAEQAAQSLLDQYAQLKAQYADKPKKRVFLQFGINPPFTSGKESIQNQVLEVCGGENIFKDS
   S_dysenteriae QVDQLASLGIKVMWVDATSIEQIANALRQLAPWSPQPDKAEQAAQSLLDQYAQLKAQYADKSKKRVFLQFGINPPFTSGKESIQNQVLEVCGGENIFKDS
   P_luminescens PLEQLAAFGIKIFYSDPTTTEQIAQDLERLAEYSPHPEQAKKSATELRQRFANLQQQYATTTPKPAFLQFGTYPLFTTSGQTLQSEVLSICGGRNIFANS
        Y_pestis PLDQLAALGIPIFYSDPTHIDQIASDLDKLAQYSPHPEQAHQAAEQFRQHVNTLRDRYARSQPKRTFLQFGTQPLFTSSGHTLQSEVVSLCGGENIFADS
           ruler .......110.......120.......130.......140.......150.......160.......170.......180.......190.......200

                --------------------XXXXXXXXXX--------------------------------------------------
      E_coli_K12 RVPWPQVSREQVLARSPQAIVITGGPDQIPKIKQYWGEQLKIPVIPLTSDWFERASPRIILAAQQLCNALSQVD-------
   E_coli_CFT073 RVPWPQVSREQVLARSPQAIVITGGPDQIPKIKQYWGEQLKIPVIPLTSDWFERASPRIILAAQQLCNALSQVD-------
S_enterica_CT18 RVPWPQVSREQVLARHPQAIIVAGKAGEILKIEQYWGNLLKIPVIPLNSDWFERASPRIILAAKQLCNALSQVN-------
 S_enterica_Ty2 RVPWPQVSREQVLARHPQAIIVAGKAGEILKIEQYWGNLLKIPVIPLNSDWFERASPRIILAAKQLCNALSQVN-------
        S_boydii RVPWPQVSREQVLARSPQAIVITGGPDQIPKIKQYWGEQLKIPVIPLTSDWFDVQAH--VLSSLHNSSVMRFHR-------
   S_dysenteriae RVPWPQVSREQVLARSPQAIVITGGPDQIPKIKQYWGEQLKIPVIPLTSDWFERASPRIILAAQQLCNALSQVD-------
   P_luminescens PVPWPQVSREQVLIRKPEIIVISGGQEQVKLIENFWHPQLRAKVITLHEDWFHRAGPRIILAAEQLCQQLNDNGS------
        Y_pestis RVPWPQVSREQVMTRKPQVIVVSGTQSQVDNVSAFWLPQLVVPVIALNEDWFNRASPRILLAAQQLCQQMASIPTPVAESH
           ruler .......210.......220.......230.......240.......250.......260.......270.......280.
```

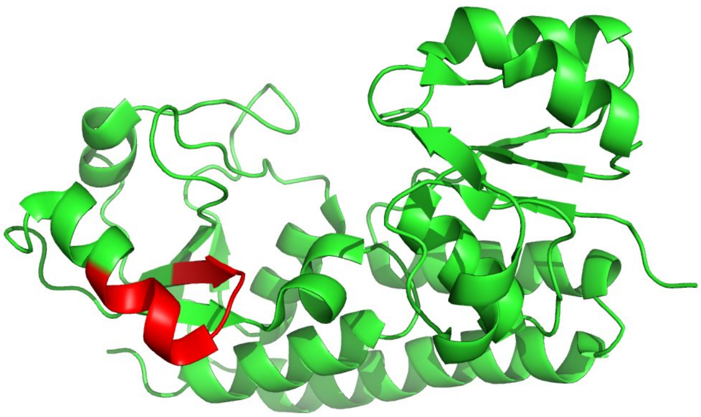

Figure S1.4: Sequence alignment and tertiary structure for gene *map*. The mutation cluster is highlighted in red.

E. coli K12 entry: b0168; PDB ID: 2gg2; chain ID: A

```
                ----------------------------------------------------------------------------------------------------
     E_coli_K12 --------MAISIKTPEDIEKMRVAGRLAAEVLEMIEPYVKPGVSTGELDRICNDYIVNEQHAVSACLGYH------GYPKSVCISINEVVCHGIPDDAK
  E_coli_CFT073 --------MAISIKTPEDIEKMRVAGRLAAEVLEMIEPYVKPGVSTGELDRICNDYIVNEQHAVSACLGYH------GYPKSVCISINEVVCHGIPDDAK
S_enterica_CT18 --------MAISIKTSEDIEKMRVAGRLAAEVLEMIEPYIKPGVTTGELDRICNDYIVNEQHAISACLGYH------GYPKSVCISINEVVCHGIPDDAK
 S_enterica_Ty2 --------MAISIKTSEDIEKMRVAGRLAAEVLEMIEPYIKPGVTTGELDRICNDYIVNEQHAISACLGYH------GYPKSVCISINEVVCHGIPDDAK
       S_boydii --------MAISIKTPEDIEKMRVAGRLAAEVLEMIEPYVKPGVSTGELDRICNDYIVNEQHAVSACLGYH------GYPKSVCISINEVVCHGIPDDAK
 S_dysenteriae --------MAISIKTPEDIEKMRVAGRLAAEVLEMIEPYVKPGVSTGELDRICNDYIVNEQHAVSACLGYH------GYPKSVCISINEVVCHGIPDDAK
  P_luminescens --------MAISIKTSEDIKKMRVAGRLAAEVLEIIEPYVKPGVTTGELDRICHDHITNKQQAVSACLGYH------GFPKSVCISVNDVVCHGIPSDDK
       Y_pestis --------MAISIKTPEDIQKMRVAGRLAAEVLEIIEPYVKPGVTTGELDRICHDHITNHQQAISACLGYH------GFPKSVCISVNEVICHGIPSDEK
   B_aphidicola -------MTCIFIKNINEINKMRLVGKLVADVLDMIKEYIVPGITTEELNNICHNYITYKQHAKPACLGYQ------GFPKSICTSINDIVCHGIPNKNS
  A_ferrooxidans MLQRSKTSPQGSIKTAEEVEKMRVAGRLTAMVLKMIGPHVKAGVTTDELDRICHDYIVNDLQGIPAPLHYQPSPEYPPFPKSVCISLNHVICHGIPGE-R
   L_pneumophila --------MGVTIKTPDEIEKMRVAGRLAAEVLEMIGPYVQVGITTDELNTICHDYIVNVQKAIPAPLNYN------GFPKSICTSINHVVCHGIPGK-K
          ruler 1.......10........20........30........40........50........60........70........80........90.......100

                ----------------------------------------------------------------------------------------------------
     E_coli_K12 LLKDGDIVNIDVTVIKDGFHGDTSKMFIVGKPTIMGERLCRITQESLYLALRMVKPGINLREIGAAIQKFVEAEGFSVVREYCGHGIGRGFHEEPQVLHY
  E_coli_CFT073 LLKDGDIVNIDVTVIKDGFHGDTSKMFIVGKPTIMGERLCRITQESLYLALRMVKPGINLREIGAAIQKFVEAEGFSVVREYCGHGIGRGFHEEPQVLHY
S_enterica_CT18 HLKDGDIVNIDVTVIKDEFHGDTSKMFIVGKPTILGERLCRVTQESLYLGIKMVKPGIRLRTIGAAIQKYAEGEGFSVVREYCGHGIGRGFHEEPQVLHY
 S_enterica_Ty2 HLKDGDIVNIDVTVIKDEFHGDTSKMFIVGKPTILGERLCRVTQESLYLGIKMVKPGIRLRTIGAAIQKYAEGEGFSVVREYCGHGIGRGFHEEPQVLHY
       S_boydii LLKDGDIVNIDVTVIKDGFHGDTSKMFIVGKPTIMGERLCRITQESLYLALRMVKPGINLREIGAAIQKFVEAEGFSVVREYCGHGIGRGFHEEPQVLHY
 S_dysenteriae LLKDGDIVNIDVTVIKDGFHGDTSKMFIVGKPTIMGERLCRITQESLYLALRMVKPGINLREIGAAIQKFVEAEGFSVVREYCGHGIGRGFHEEPQVLHY
  P_luminescens TLKDGDIVNIDVTVIKEGFHGDTSKMFIVGKPTIQGERLCRITQESLYLALKMVKPGIRLRTLGRAIQQFVEANDFSVVREYCGHGIGAGFHEEPQVLHY
       Y_pestis VLKEGDIVNIDVTVIKDGFHGDTSKMFIVGKPTILGERLCRVTQESLYLAIKMVKPGIRLRTLGKAIQKFVEAENFSVVREYCGHGIGEGFHEEPQVLHY
   B_aphidicola ILKNGDIINIDVAILKDKYYSDASKMFFVGKPTELGKKLCYVAKKSLYLALYTIRPGINLQKLGKVIQNYVKKQNFSIVKEYCGHGIGRSFHEPPQILHH
  A_ferrooxidans VIRDGDMLNIDVTVIKDGYHGDSSKMFTVGEVPLRSRRVMEVAHEAMVRGIQQVRPGATLGDIGHAIQSYAEAQHCSIVREFCGHGIGRKFHDEPQVLHY
   L_pneumophila TLKDGDIINIDVTVIKNEYHGDTSKMFIVGTPSVKAKHVVQIAHECLYIGIDMVKPGVQLGDIGHAIQQHAEKNRCSVVRDYCGHGIGRVFHEDPQVLHY
          ruler .......110.......120.......130.......140.......150.......160.......170.......180.......190.......200

                -X-XXXX--------------------------------------------------------------------------
     E_coli_K12 DS-RETNVVLKPGMTFTIEPMVNAGKKEIRTMKDGWTVKTKDRSLSAQYEHTIVVTDNGCEILTLRKDDT--IPAIISHDE
  E_coli_CFT073 DS-RETNVVLKPGMTFTIEPMVNAGKKEIRTMKDGWTVKTKDRSLSAQYEHTIVVTDNGCEILTLRKDDT--IPAIISHDE
S_enterica_CT18 DA-DDGGVVLQPGMTFTIEPMLNAGDYRIRTMKDGWTVKTKDRSLSAQYEHTIVVTENGCEILTLRKDDT--IPAIITHDE
 S_enterica_Ty2 DA-DDGGVVLQPGMTFTIEPMLNAGDYRIRTMKDGWTVKTKDRSLSAQYEHTIVVTENGCEILTLRKDDT--IPAIITHDE
       S_boydii DS-RETNVVLKPGMTFTIEPMVNAGKKEIRTMKDGWTVKTKDRSLSAQYEHTIVVTDNGCEILTLRKDDT--IPAIISHDE
 S_dysenteriae DS-RETNVVLKPGMTFTIEPMVNAGKKEIRTMKDGWTVKTKDRSLSAQYEHTIVVTDNGCEILTLRKDDT--IPAIISHDE
  P_luminescens DA-DDSGVVLQKGMAFTIEPMVNTGDYHIRTMKDGWTVKTKDRSLSAQYEHTIVVTDNGCEIMTLRKEEEPALSSVLVNE-
       Y_pestis DA-DDGGVVLQAGMAFTIEPMVNAGDYRIRTMKDGWTVKTKDRNLSAQYEHTIVVTDNGCEIMTLRKDDT--IPNIITHE-
   B_aphidicola NYYKSNTTILQSGMTFTVEPMINSGSCEVQCTNDGWTVKTKDKSLSAQYEHTILVNEEGCEILTLQKGEQ--ISRILKNLT
  A_ferrooxidans GN-PGEGIELVPGMTFTIEPMVNAGKRYIKALPDGWTIVTKDHSASAQWEHTILVTDDGYEILTQLPGDP--V--------
   L_pneumophila GV-PGTGMRLEPGMTFTIEPMVNIGKHHTRLLPDHWTVVTKDHSLSAQWEHTLLVTDTGVEILTLRNEER----------
          ruler .......210.......220.......230.......240.......250.......260.......270.......280.
```

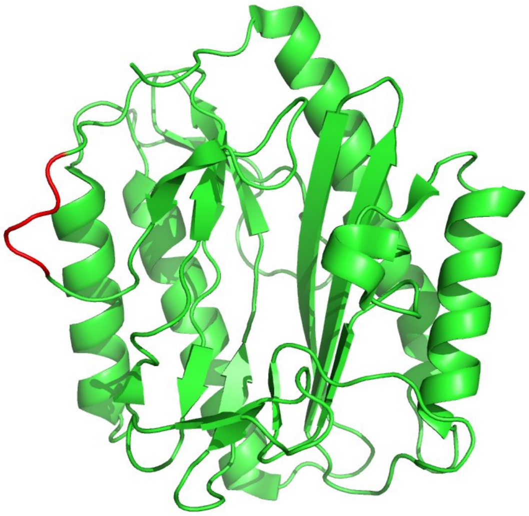

Figure S1.5: Sequence alignment and tertiary structure for gene *ispU*. The mutation cluster is highlighted in red.

E. coli K12 entry: b0174; PDB ID: 1x06; chain ID: A

```
                 ----------------------------------------------------------------------------------------------------
       E_coli_K12 -------VMLSATQPLSEKLPAHGCRHVAIIMDGNGRWAKKQGKIRAFGHKAGAKSVRRAVSFAANNGIEALTLYAFSSENWNRPAQEVSALMELFVWAL
    E_coli_CFT073 -------VMLSATQPLSEKLPAHGCRHVAIIMDGNGRWAKKQGKIRAFGHKAGAKSVRRAVSFAANNGIEALTLYAFSSENWNRPAQEVSALMELFVWAL
S_enterica_CT18 --------MLSATQPVSENLPAHGCRHVAIIMDGNGRWAKKQGKIRAFGHKAGAKSVRRAVSFAANNGIDALTLYAFSSENWNRPAQEVSALMELFVWAL
 S_enterica_Ty2 --------MLSATQPVSENLPAHGCRHVAIIMDGNGRWAKKQGKIRAFGHKAGAKSVRRAVSFAANNGIDALTLYAFSSENWNRPAQEVSALMELFVWAL
         S_boydii -------VMLSATQPLSEKLPAHGCRHVAIIMDGNGRWAKKQGKIRAFGHKAGAKSVRRAVSFAANNGIEALTLYAFSSENWNRPAQEVSALMELFVWAL
    S_dysenteriae -------VMLSATQPLSEKLPAHGCRHVAIIMDGNGRWAKKQGKIRAFGHKAGAKSVRRAVSFAANNGIEALTLYAFSSENWNRPAQEVSALMELFVWAL
    P_luminescens ----VILSSDHHQNDLSSLLPK----HVAIIMDGNGRWAKKRGKLRAFGHRAGIKAVRSAVSFSAKHNIESLTLYAFSSENWNRPEQEVSSLMELFIFAL
        Y_pestis -----MSPVKEDRANLSPRSPR----HVAIIMDGNGRWAKNKGKLRVFGHKAGVKSVRRAVSFAAKHNLDALTLYAFSSENWNRPDQEVTALMELFVRAL
     B_aphidicola MSYSKNNMLFKNVLNFKKNIPS----HVAIIMDGNGKWARKRGKSRFFGHFSGFYAARRAISFALFHKLKILTLYAFSSDNWNRSPREIKVLMELFFYAL
    A_ferrooxidans -------------------MPR----HIAVIMDGNGRWAYRRHLPRVAGHRRGAEVVREMVQSCADLGIPYLTLFAFSTENWRRPAIEVRLLMNLFRLLL
     L_pneumophila --------------LNHKLPQ----HIAVVMDGNGRWAESRGLPRVEGHKAGLDSVKKIINCCLEKKISCLSLFAFSSENWSRPVTEVNFLMELFLEAL
            ruler 1.......10........20........30........40........50........60........70........80........90.......100
```

```
                 -----------------------------------------------------------------------------------XXXXXX-----------
       E_coli_K12 DSEVKSLHRHNVRLRIIGDTSRFNSRLQERIRKSEALTAGNTGLTLNIAANYGGRWDIVQGVRQLAEKVQQGNLQPDQIDEEMLNQHVCMHELAPVDLVI
    E_coli_CFT073 DSEVKSLHRHNVRLRIIGDTSRFNSRLQERIRKSEALTAGNTGLTLNIAANYGGRWDIVQGVRQLAEKVQQGNLQPDQIDEEMLNQHVCMHELAPVDLVI
S_enterica_CT18 DSEVKSLHRHNVRLRIIGDISRFNSRLQERIRKSEALTAHNTGLTLNIAANYGGRWDIVQGVRQLAELVQAGVLRPDQIDEERLGQQICMHELAPVDLVI
 S_enterica_Ty2 DSEVKSLHRHNVRLRIIGDISRFNSRLQERIRKSEALTAHNTGLTLNIAANYGGRWDIVQGVRQLAELVQAGVLRPDQIDEERLGQQICMHELAPVDLVI
         S_boydii DSEVKSLHRHNVRLRIIGDTSRFNSRLQECIRKSEALTAGNTGLTLNIAANYGGRWDIVQGVRQLAEKVQQGNLQPDQIDEEMLNQHVCMHELAPVDLVI
    S_dysenteriae DSEVKSLHRHNVRLRIIGDTSRFNSRLQERIRKSEALTAGNTGLTLNIAANYGGRWDIVQGVRQLAEKVQQGNLQPDQIDEEMLNQHVCMHELAPVDLVI
    P_luminescens DSEIKSLHKHNIRLSVIGDIGRFSERLQDRIHRSVKLTANNTGLQLNIAANYGGRWDIVQSVQKIAQQIKDNSLEQQDITEELVNNYMNLSQQPQVDLVI
        Y_pestis DSEVKSLHKHNVRLSIIGDISRFSGRLQERIRRSEKLTANNDGLKLNIAANYGGRWDIIQGVRHLAEQVQKGELQPTDISEESLNSYICLHEQSQVDLVI
     B_aphidicola SNETNNLNKYNIRLKVIGNKEKFNTVLKNKIRVVEKETLKNTGLLLNIAANYSGRWEILEAIKKIVVAIKCKNLSLNAITESTVSDFLLINEKIPVDLVI
    A_ferrooxidans RREARKLHENGVRLRIIGDRSALDPEICQLIDEAEALTRNNQRLQLNLAVNYGGRWDIAQAARAAMAAVQAGDLALEDFSAAHIARHLCLADIPEPDLLI
     L_pneumophila RKEIDDLNQHGIRLKFTGDREPLSQVLQKQMSDAEALTKNNQQLILNVVVNYGGKWDIVTAARKLIRTVLDGKLAYDEINEAVFAQFLDTKGMPEPDLFI
            ruler .......110.......120.......130.......140.......150.......160.......170.......180.......190.......200
```

```
                 ------------------------------------------------------------------
       E_coli_K12 RTGGEHRISNFLLWQIAYAELYFTDVLWPDFDEQDFEGALNAFANRERRFGGT----EPGDETA--
    E_coli_CFT073 RTGGEHRISNFLLWQIAYAELYFTDVLWPDFDEQDFEGALNAFANRERRFGGT----EPGDETA--
S_enterica_CT18 RTGGEHRISNFLLWQIAYAELYFTDVLWPDFDEQDFEGALHAFANRERRFGGT----EPGDDKA--
 S_enterica_Ty2 RTGGEHRISNFLLWQIAYAELYFTDVLWPDFDEQDFEGALHAFANRERRFGGT----EPGDDKA--
         S_boydii RTGGEHRISNFLLWQIAYAELYFIDVLWPDFDEQDFEGALNAFANRERRFGGT----EPGDETA--
    S_dysenteriae RTGGEHRISNFLLWQIAYAELYFTDVLWPDFDEQDFEGALNAFANRERRFGGT----EPGDETA--
    P_luminescens RTGGEHRISNFLLWQIAYAEFYFTDILWPDFDENVFEGAINAFAKRERRFGGT----IPDDADVGS
        Y_pestis RTGGEHRISNFLLWQIAYAELYFTDVLWPDFDENVFEGALNAFAQRERRFGGT----TPIDATAS-
     B_aphidicola RTGGECRLSNFLVWQISYSELYFTNTLWPDFDRKEFKKAIDEFSNRERRFGR-----------VSH
    A_ferrooxidans RTGGEERISNFLLWQLAYTEFYFSDTLWPDFNRAALEHALHSFAHRQRRFGRTGDQVLEGDCSVSD
     L_pneumophila RTSGELRISNFFLWQLAYTELYFTDVHWPDFNEHEFELALISFSKRARRFGQI---------SQSE
            ruler .......210.......220.......230.......240.......250.......260......
```

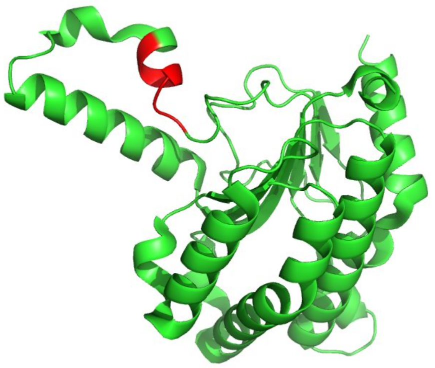

Figure S1.6: Sequence alignment and tertiary structure for gene *codA*. The mutation cluster is highlighted in red.

E. coli K12 entry: b0337; PDB ID: 1ra0; chain ID: A

```
                --------------------------------XXXXXXXXXXXXXXXXXXXXXXX---------------------------------------------
       E_coli_K12 ---------VSNNALQTIINARLPGEEGLWQIHLQDGKISAIDAQSGVMPITENSLDAEQGLVIPPFVEPHIHLDTTQTAGQPNWNQSGTLFEGIERWAE
   E_coli_CFT073 ----MWRLTVSNNALQTIINARLPGKEGLWQIHLHDGKISAIDTQSGVMPVTENSLDAEQGLVLPPFVEPHIHLDTTQTAGQPNWNQSGTLFEGIERWAE
 S_enterica_CT18 ---------MQNNNI-TIRQTRLQGHEGLWQITIENGRFSRIEPQEATSLPQGEVLDAEGGLAIPPFVEPHIHLDTTQTAGEPSWNQSGTLFEGIERWAE
  S_enterica_Ty2 ---------MQNNNI-TIRQTRLQGHEGLWQITIENGRFSRIEPQEATSLPQGEVLDAEGGLAIPPFVEPHIHLDTTQTAGEPSWNQSGTLFEGIERWAE
        Y_pestis VEALMAGQSVPNYSLTTVNNVRRIGLAGLWQITIAEGKISLIQPQPEQSLTGQGVLDAQGGLALPPFIEPHIHLDTTQTAGQPSWNQSGTLFEGIERWAE
           ruler 1.......10........20........30........40........50........60........70........80........90.......100

                 ----------------------------------------------------------------------------------------------------
       E_coli_K12 RKALLTHDDVKQRAWQTLKWQIANGIQHVRTHVDVSDATLTALKAMLEVKQEVAPWIDLQIVAFPQEGILSYPNGEALLEEALRLGADVVGAIPHFEFTR
   E_coli_CFT073 RKALLTHDDVKQRAWQTLKWQIANGIQHVRTHVDVSDATLTALKAMLEVKQEVAPWIDLQIVAFPQEGILSYPNGEALLEEALRLGADVVGAIPHFEFTR
 S_enterica_CT18 RKAMLTHEDVKARAMQTLKWQMANGIQYVRTHVDVSDPTLTALKAMLEVKQEVAPWVDLQIVAFPQEGILSYPNGEALLEEAVRLGADVIGAIPHFEFTR
  S_enterica_Ty2 RKAMLTHEDVKARAMQTLKWQMANGIQYVRTHVDVSDPTLTALKAMLEVKQEVAPWVDLQIVAFPQEGILSYPNGEALLEEAVRLGADVIGAIPHFEFTR
        Y_pestis RKARLTREDVKQRAWQTLKWQIANGIQHVRTHVDVSDPHLTALSAMLEVKEEVSPWVDMQIVAFPQEGILSYPDGAALLEEALRLGADAVGAIPHFEFTR
           ruler .......110.......120.......130.......140.......150.......160.......170.......180.......190.......200

                 ----------------------------------------------------------------------------------------------------
       E_coli_K12 EYGVESLHKTFALAQKYDRLIDVHCDEIDDEQSRFVETVAALAHHEGMGARVTASHTTAMHSYNGAYTSRLFRLLKMSGINFVANPLVNIHLQGRFDTYP
   E_coli_CFT073 EYGVESLHKTFALAQKYDRLIDVHCDEIDDEQSRFVETVAALAHREGMGARVTASHTTAMHSYNGAYTSRLFRLLKMSGINFVANPLVNIHLQGRFDTYP
 S_enterica_CT18 EYGVESLHKTFALAQKYDRLIDVHCDEIDDEQSRFVETVAALAHRDGMGARVTASHTTAMHSYNGAYASRLFRLLKMSGINFVANPLVNIHLQGRFDTYP
  S_enterica_Ty2 EYGVESLHKTFALAQKYDRLIDVHCDEIDDEQSRFVETVAALAHRDGMGARVTASHTTAMHSYNGAYASRLFRLLKMSGINFVANPLVNIHLQGRFDTYP
        Y_pestis EYGVESLHIAFALAQKYQRLVDVHCDETDDEQSRFIETVAALALRENMGARVTASHTTAMHSYNGAYTSRLFRLLKLSGINFVANPLVNIHLQGRFDTYP
           ruler .......210.......220.......230.......240.......250.......260.......270.......280.......290.......300
```

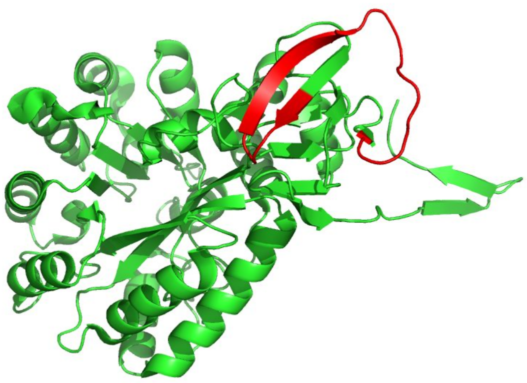

Figure S1.7: Sequence alignment and tertiary structure for gene *maa*. The mutation cluster is highlighted in red.

E. coli K12 entry: b0459; PDB ID: 1ocx; chain ID: A

```
                 ---------------------------------XXXXXXXXXXXXXXXXXXXXXXXXXXX----------------------------------------
       E_coli_K12 MSTEKEKMIAGELYRSADETLSRDRLRARQLIHRYNHSLAEEHTLRQQILADLFGQVTEAYIEPTFRCDYGYNIFLGNNFFANFDCVMLDVCPIRIGDNC
    E_coli_CFT073 MSTEKEKMIAGELYHSADEALSRDRLRARRLIHRYNHSLAEEHTLRQQILADLFGQVTEAYIEPTFRCDYGYNIFLGNNFFANFDCVMLDVCPIRIGDNC
S_enterica_CT18 MSDEKQKMIAGALYCPTDETLCQDRLRARQLIHQYNYTTPDEINKRQAILRDLLGRCEDAYIEPSFRCDYGYNIFLGHSFYANFDCVMLDVCPIHIGDNC
 S_enterica_Ty2 MSDEKQKMIAGALYCPTDETLCQDRLRARQLIHQYNYTTPDEINKRQAILRDLLGRCEDAYIEPSFRCDYGYNIFLGHSFYANFDCVMLDVCPIHIGDNC
        S_boydii MSTEKEKMIAGELYRSADETLSRDRLRARQLIHRYNHSLAEEHTLRQQILADLFGQVTEAYIEPTFRCDYGYNIFLGNNFFANFDCVMLDVCPIRIGDNC
    S_dysenteriae MSTEKEKMIAGELYRSADETLSRDRLRARQLIHRYNHSLAEEHTLRQQILADLFGQVTEAYIEPTFRCDYGYNIFLGNNFFANFDCVMLDVCPIRIGDNC
            ruler 1.......10........20........30........40........50........60........70........80........90.......100

                 -----------------------------------------------------------------------------------
       E_coli_K12 MLAPGVHIYTATHPIDPVARNSGAELGKPVTIGNNVWIGGRAVINPGVTIGDNVVVASGAVVTKDVPDNVVVGGNPARIIKKL
    E_coli_CFT073 MLAPGVHIYTATHPIDPVARNSGAELGKPVTIGNNVWIGGRAVINPGVTIGDNVVVASGAVVTKDVPDNVVVGGNPARIIKKL
S_enterica_CT18 MLAPGVHIYTATHPLDAVERNSGRELGKPVTIGNNVWIGGRAVVNPGVTIGDNVVVASGAVVTKNVPPDVVVGGNPARIIKKL
 S_enterica_Ty2 MLAPGVHIYTATHPLDAVERNSGRELGKPVTIGNNVWIGGRAVVNPGVTIGDNVVVASGAVVTKNVPPDVVVGGNPARIIKKL
        S_boydii MLAPGVHIYTATHPIDPVARNSGAELGKPVTIGNNVWIGGRAVINPGVTIGDNVVVASGAVVTKGVPDNVVVGGNPARIIKKL
    S_dysenteriae MLAPGVHIYTATHPIDPVARNSGAELGKPVTIGNNVWIGGRAVINPGVTIGDNVVVASGAVVTKDVPDNVVVGGNPARIIKKL
            ruler .......110.......120.......130.......140.......150.......160.......170.......180...
```

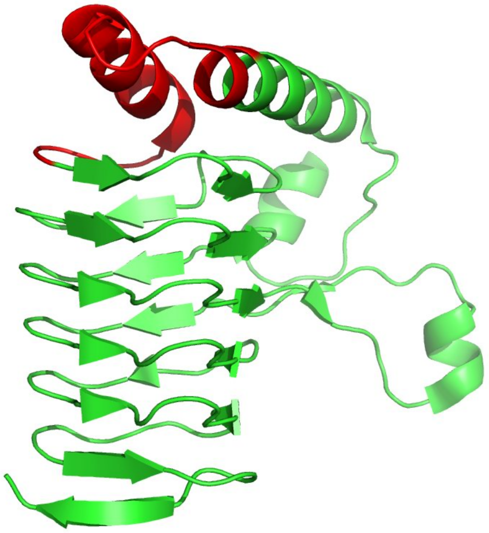

Figure S1.8: Sequence alignment and tertiary structure for gene *1bs0*. The mutation cluster is highlighted in red.

E. coli K12 entry: b0776; PDB ID: 1bs0; chain ID: A

```
                ----------------------------------------------------------------------------------------------------
     E_coli_K12 --MSWQEKINAALDARRAADALRRRYPVAQGAGR----WLVADDRQYLNFSSNDYLGLSHHPQIIRAWQQGAEQFGIGSGGSGHVSGYSVVHQALEEELA
  E_coli_CFT073 --MIWQEKIDAALDARRVADALRRRYPVAQGAGR----WLVADDCQYLNFSSNDYLGLSHHPQIIRAWQQGADQFGVGSGGSGHVSGYSVAHQVLEEELA
S_enterica_CT18 --MSWQQRVDDALTARRATDTLRRRYVVSQGAGR----WLVANGRQYLNFSSNDYLGLSQHPQIIRAWQQAATRFGVGSGGSGHISGYSVAHQALEEELA
 S_enterica_Ty2 --MSWQQRVDDALTARRATDTLRRRYVVSQGAGR----WLVANGRQYLNFSSNDYLGLSQHPQIIRAWQQAATRFGVGSGGSGHISGYSVAHQALEEELA
       S_boydii --MSWQEKINAALDARRAADALRRRYPVAQGAGR----WLVADDRQYLNFSSNDYLGLSHHPQIIRAWQQGAEQFGVGSGGSGHVSGYSVAHQALEEELA
  S_dysenteriae --MSWQEKINAALDARRAADALRRRYPVAQGAGR----WLVADDRQYLNFSSNDYLGLSHHPQIIRAWKQGAEQFGVGSGGSGHVSGYSVAHQALEEELA
  P_luminescens --MDWSSYLSRQLSERRSTSLWRQRQVNQKSDGR----FLYAADGKYLNFSSNDYLGLSANPAIISAWQRGAEQYGVGSGGSGHITGYTKAHQLLEQQLA
       Y_pestis --MSWQDKIAQGLQRRRDAAAYRTRQVNEGANGR----WLQSGERQYLNFSSNDYLGLSQNDEVIAAWQQGARRYGVGSGGSGHVTGYSQPHARLEQQLA
   B_aphidicola --MNWNKRINHKLNMHIFNKKFRVKVAVQKNNNR----IINVNGMQYINFSSNDYLGLRNNARIVQAWKTAATRYGIGSTGSSLITGYSTIHQSLEEKLA
 A_ferrooxidans MHSEREESWRAELSALRAHDLWRELQVLQPAPERGPPTFVGTRGEPLLSFASNDYLGLSAESALRDAAIAEIQQSGVGAGAAPLLGGERPAHAVLANALA
  L_pneumophila --MTLNIKINEYTAQLQEQGLLRTRQ------------ILETDKIDYIRFDSNDYLSLTEDRRIAEAYQKGYELYPCGSGASMVLSGYHSSHQTLERAFA
          ruler 1.......10........20........30........40........50........60........70........80........90.......100

                -------------------------------------------------------------------------------------------------XXX
     E_coli_K12 EWLGYSRALLFISGFAANQAVIAAMMAKEDRIAADRLSHASLLEAASLSPSQLRRFAHNDVTHLARLLASPCPGQQMVVTEGVFSMDGDSAPLAEIQQVT
  E_coli_CFT073 EWLGYSRALLFISGFAANQAVIAAMMAKEDRIVADRLSHASLLEAASLSPSPLRRFAHNDVTHLARLLASPCPGQQLVVTEGVFSMDGDSAPLEEIQQVT
S_enterica_CT18 QWLGYPRALLFISGFAANQAVITALMKKNDRIVADRLSHASLLEAANLSPAQLRRFIHNDTQHLSRLLQSPCVGQQLVVTEGVYSMDGDSAPLAEIQHIA
 S_enterica_Ty2 QWLGYPRALLFISGFAANQAVITALMKKNDRIVADRLSHASLLEAANLSPAQLRRFIHNDTQHLSRLLQSPCVGQQLVVTEGVYSMDGDSAPLAEIQHIA
       S_boydii EWLGYSRALLFISGFAANQAVIAAMMAKEDRIVADRLSHASLLEAASLSPSQLRRFVHNDVTHLARLLASPCPGQQLVVTEGVFSMDGDSAPLAEIQQVT
  S_dysenteriae EWLGYSRALLFISGFAANQAVIAAMMAKEDRIVADRLSHASLLEAASLSPSQLRRFVHNDVTHLARLLASPCPGQQLVVTEGVFSMDGDSAPLGEIQQVT
  P_luminescens DWLGYPKALLFISGYSANQGVIAALMTEQDRIIADRLSHASLMEAAMHSPAQLRRFLHNQPESLANLLATPCVGKTLVVTEGVFSMDGDCAPLADIHQQT
       Y_pestis DWLGYPRALLFISGYAANQAVLTALTDADDRILADKLSHASLLEAAAHSPAQLRRFQHNQPEALQNLLIKPCQGQTLVVTEGVFSMDGDSAPLAALQQQT
   B_aphidicola KWLDYPKAILFISGYTANTAIISTLIQKNDRIFMDKLSHSSILEPSYNSSGKCYRFIHNNPSSLMNKFYSSSGKNPLIITEGIFSMDGDIAPLSIISSFS
 A_ferrooxidans RWLGVEAALLFGSGYLANLGVISTLVGRGDRVYADRLNHASLVDGVRLSGARLHRYRHGDMTHLAQWLERGGRGQAWIITDGVFSMDGDIAPLPELATLA
  L_pneumophila EMLRVDDCLLFSSGYCANLAVTALLGRLGTHCFIDKSVHASIYDGLALSRVTYSRYIHNDMNALPALL-KSHLGDSVLITEGIFSMSGQIAPLSTISMLC
          ruler .......110.......120.......130.......140.......150.......160.......170.......180.......190.......200

                XXXXXXXXXXXXXXXXXXXXXXXXXX--XXXX--------------------------------------------------------------------
     E_coli_K12 QQHNGWLMVDDAHGTGVIGEQGRGSC--WLQKVKP-ELLVVTFGKGFGVSGAAVLCSSTVADYLLQFARHLIYSTSMPPAQAQALRASLAVIRSDEGDAR
  E_coli_CFT073 QQHDGWLMVDDAHGTGVIGEQGRGSC--WLQKVKP-ELLVVTFGKGFGVSGAAVLCSNTVADYLLQFARHLIYSTSMPPAQAQALRASLAVIRSDEGDAR
S_enterica_CT18 RRHHAWLLVDDAHGIGVTGDEGRGTC--WQRGVKP-ELLVVTFGKGFGVSGAAVLCSESVADYLLQFARHLVYSTSMPPAQAQALSASLAVIRSDEGRER
 S_enterica_Ty2 RRHHAWLLVDDAHGIGVTGDEGRGTC--WQRGVKP-ELLVVTFGKGFGVSGAAVLCSESVADYLLQFARHLVYSTSMPPAQAQALSASLAVIRSDEGRER
       S_boydii QQHNGWLMVDDAHGTGVIGEQGRGSC--WLQKVKP-ELLVVTFGKGFGVSGAAVLCSSTVADYLLQFARHLIYNTSMPPAQAQALRASLAVIRRDEGDAR
  S_dysenteriae QQHNGWLMVDDAHGTGVIGEQGRGSC--WLQKVKP-ELLVVTFGKGFGVSGAAVLCSSTVADYLLQFARHLIYSTSMPPAQAQALRASLAVIRSDEGDAR
  P_luminescens RISGNWLMVDDAHGIGVCGEQGRGSC--WQQKVKP-EILIVTFGKAFGLSGAAVLCDEQTAEYLIQYARHLIYSTSMPPAQAQALSEAVRQIQS--GDEL
       Y_pestis SAAGGWLLVDDAHGIGVHGEGGRGSC--WLQGVQP-ELLVVTFGKAFGLSGAAVLCQEPVAEYLLQYARHLIYSTAMPPAQACALQAALRQVQQ--GDAL
   B_aphidicola KKIKGLLMVDDAHGIGVSGYNGKGSC--EQHRVKP-DILTITFGKAFGISGAAVLCSNNIAEYLWQFSKHLMFSTAMPIAQAYAIRQALYCIQH--ADKL
 A_ferrooxidans QQYGAGIILDEAHAFGVLGTEGQGTAAHWNMDIHGVDVIMGTLGKAFGVYGAFVAGSQDVVDLLRNRARSFIYHTALPSALAAAALVALDLLRH--GDAR
  L_pneumophila KENGSELFVDEAHAFGVMGPQGMGSVPGHGLTQNEVPLRVIPFGKAFASQGAVVVGRKDWIHALLQTARSFVYSTAISPALCYGLLKTLEIIAA--ADSR
          ruler .......210.......220.......230.......240.......250.......260.......270.......280.......290.......300
```

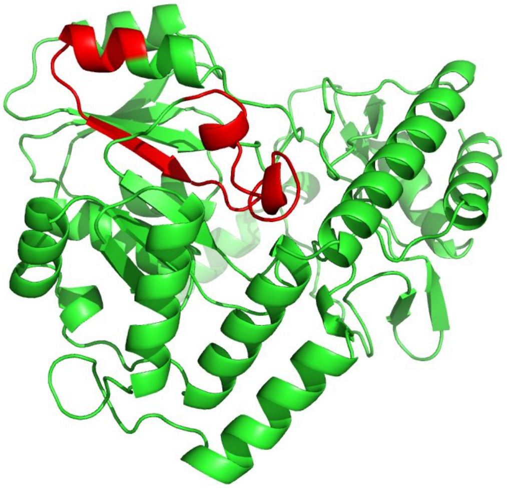

Figure S1.9: Sequence alignment and tertiary structure for gene *pepN*. The mutation cluster is highlighted in red.

E. coli K12 entry: b0932; PDB ID: 2dq6; chain ID: A

```
                  ----------------------------------------------------------------------------------------------------
       E_coli_K12 KVIPLQKGGHPVNSVLNVTQAEQTFVFDNVYFQPVPALLCEFSAPVKLEYKWSDQQLTFLMRHARNDFSRWDAAQSLLATYIKLNVARHQQGQPLSLPVH
    E_coli_CFT073 KVIPLQKGGHPVNSVLNVTQAEQTFVFDNVYFQPVPALLCEFSAPVKLEYKWSDQQLTFLMRHARNDFFRWDAAQSLLATYIKLNVARHQQGQPLSLPVH
 S_enterica_CT18 NVIPLQKGGHPVNAVLNVTQAEQTFTFDNVYFQPVPALLCEFSAPVKLEYKWSDQQLTFLMRHARNDFSRWDAAQSLLATYIKLNVARHQQGQPLSLPVH
  S_enterica_Ty2 NVIPLQKGGHPVNAVLNVTQAEQTFTFDNVYFQPVPALLCEFSAPVKLEYKWSDQQLTFLMRHARNDFSRWDAAQSLLATYIKLNVARHQQGQPLSLPVH
         S_boydii KVISLQKGGHPVNSVLNVTQAEQTFVFDNVYFQPVPALLCEFSAPVKLEYKWSDQQLTFLMRHARNDFSRWDAAQSLLATYIKLNVARHQQGQPLSLPVH
    S_dysenteriae KVIPLQKGGHPVNSVLNVTQAEQTFVFDNVYFQPVPALLCEFSAPVKLEYKWSDQQLTFLMRHARNDFSRWDAAQSLLATYIKLNGARHQQGQPLSLPVH
    P_luminescens HVVPLRYDGQPVHNVLNVTNAEQTFVFDDVPSLPVPSLLREFSAPVKLDYTFSDQQLAFLMKHARNAFSRWDAAQSLMANYIKLNIIRYQQKQPIELPMH
         Y_pestis NVIPLQHNGLPVHHVLNVTEAEQTFTFDNVAQKPIPSLLREFSAPVKLDYPYSDQQLTFLMQHARNEFSRWDAAQSLLATYIKLNVAKYQQQQPLSLPAH
  A_ferrooxidans QRVPLEVVGGASETVLLLEQSEQSWNFANLPGPVIPSLLRGFSAPVRLQDSLDDDAHGFLARHDDDPFNRWESMQDLAVKALLAAVA-DSSVAP--LPIT
    L_pneumophila EMIPIE------NELLELKEKEQRFIFSGLNEKPVVSLLREFSAPVKIYDDLDQDDLLFLLRYETDGYAKWDAAQRLVLNCLNENLKLPASEWQ--VSKT
            ruler .......510.......520.......530.......540.......550.......560.......570.......580.......590.......600

                  ---------------------------XXXXXXXXXXXXXXXXXXXXXXXXXXXXXXXXXXXXXXXXXXX-XXXXXXXXXXXXXXXXXXXXXXXXXXXX-
       E_coli_K12 VADAFRAVLLDEKIDPALAAEILTLPSVNEMAELFDIIDPIAIAEVREALTRTLATELADELLAIYNANY-QSEYRVEHEDIAKRTLRNACLRFLAFGE-
    E_coli_CFT073 VADAFRAVLLDEKIDPALAAEILTLPSVNEMAELFDIIDPIAIAEVREALTRTLATELADELLAIYNANY-QSEYRVEHEDIAKRTLRNACLRFLAFGE-
 S_enterica_CT18 VADAFRAVLLDEKIDPALAAEILTLPSANEIAELFEVIDPIAIAQVREALTRTLAAELADEFLAIYNANH-LDEYRVDHGDIGKRTLRNACLRFLTFGE-
  S_enterica_Ty2 VADAFRAVLLDEKIDPALAAEILTLPSANEIAELFEVIDPIAIAQVREALTRTLAAELADEFLAIYNANH-LDEYRVDHGDIGKRTLRNACLRFLTFGE-
         S_boydii VADAFRAVLLDEKIDPALAAEILTLPSVNEMAELFDIIDPIAIAEVREALTRTLATELADELLAIYNANY-QSEYRVEHEDIAKRTLRNACLRFLAFGE-
    S_dysenteriae VADAFRAVLLDEKIDPALAAEILTLPSVNEMAELFDIIDPIAIAEVREALTRTLATELADELLAIYNANY-QSEYRVEHEDIAKRTLRNACLRFLAFGE-
    P_luminescens VVDAFRAVLLDKEIDPALAAHILALPSESEIAELFTIIDPKAIHEVLDTITRTLAQEMADEFAVVYNAIN-IGAYRVAHQDIAQRSLRNICLYYLAFGH-
         Y_pestis VADAFRAILLDEHLDPALAAQILTLPSENEMAELFTTIDPQAISTVHEAITRCLAQELSDELLAVYVANM-TPVYRIEHGDIAKRALRNTCLNYLAFGD-
  A_ferrooxidans LRNAVAATLADRQVDPAFCAELLTLPGEDYIGEQMPVVAVEAIHRARDGMMRAIGRHFAKEWTGLYHDL--AAAYQRDGLSIGRRRLRNLALAYLIAGEG
    L_pneumophila LVAAFQHVLLDDSLDMDLRAELLTPPGFEEVAATMKWVDVSKVESVRDYFRQQLGLGLYTEASDLYKELWEVEDHRMHGQAYGRRKLRNVCLWLMMKAK-
            ruler .......610.......620.......630.......640.......650.......660.......670.......680.......690.......700

                  --XXXXXXXXXXXXXX------------------------------------------------------------------------------------
       E_coli_K12 --THLADVLVSKQFHEANNMTDALAALSAAV-AAQLPCRDALMQEYDDKWHQNGLVMDKWFILQATSPAANVLETVRGLLQHRSFTMSNPNRIRSLIGAF
    E_coli_CFT073 --THLADVLVSKQFHEANNMTDALAALSAAV-AAQLPCRDALMQEYDDKWHQDGLVMDKWFILQATSPAANVLETVRGLLQHRAFTMSNPNRIRSLIGAF
 S_enterica_CT18 --TELANTLVSKQYRDANNMTDALAALSAAV-AAQLPCRDTLMQEYDDKWHQDGLVMDKWFILQSTSPAENVLETVRSLLKHRSFSMSNPNRIRSLIGAF
  S_enterica_Ty2 --TELANTLVSKQYRDANNMTDALAALSAAV-AAQLPCRDTLMQEYDDKWHQDGLVMDKWFILQSTSPAENVLETVRSLLKHRSFSMSNPNRIRSLIGAF
         S_boydii --THLADVLVSKQFHEANNMTDALAALSAAV-AAQLPCRDALMQEYDDKWHQDGLVMDKWFILQATSPAANVLETVRGLLQHRSFTMSNPNRIRSLIGAF
    S_dysenteriae --THLADVLVSKQYHEANNMTDALAALSAAV-AAQLPCRDALMQEYDDKWHQDGLVMDKWFILQATSPAANVLETVRGLLQHRSFTMSNPNRIRSLIGAF
    P_luminescens --QEQADKVVSNQYHQSDNMTDALAALSGAV-LAELPCCDQLMAEFDGRWHQDGLVMDKWFSLQASSPAVDVLANVRHLLNHRSFSMSNPNRVRALIGAF
         Y_pestis --EEFANKLVSSQYHQADNMTDSLAALAAAV-AAQLPCRDELLAAFDVRWNHDGLVMDKWFALQATSPAANVLVQVRTLLKHPAFSLSNPNRTRSLIGSF
  A_ferrooxidans NSREGAILHARQQYIQADNMTDRLAAFQLLA-QHRHHDAEDVILDFYQRWHEYPLVIDKWFAIQAAAPYPKTLRQVEHLLVHPAFDWRVPNRVRAVLGAF
    L_pneumophila --ESDALELCREQFKDSRTMTDQIASCSLLVNCSRESHRNEAIEGFYKQWSHNELVLDKWFAMQAACELPGTLGHVKKLTQHPAFCIKNPNKVRALVGAF
            ruler .......710.......720.......730.......740.......750.......760.......770.......780.......790.......800
```

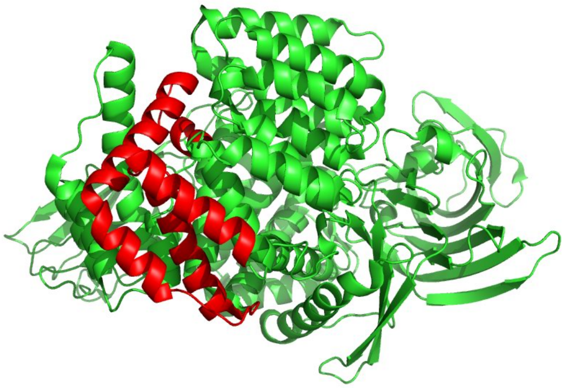

Figure S1.10: Sequence alignment and tertiary structure for gene *pabC*. The mutation cluster is highlighted in red.

E. coli K12 entry: b1096; PDB ID: 1i2k; chain ID: A

```
                  ---------------------------------XXXXXXXXXXXXXXXXXXXXXXXXXXXXXXXXXXXXXXXX---XXXXXX------------------
      E_coli_K12 ---MFLINGHKQESLAVSDRATQFGDGCFTTARVIDGKVSLLSAHIQRLQDACQRLMISCDFWPQLEQEMKTL---AAEQQNGVLKVVISRGSGGRGYST
   E_coli_CFT073 ---MFLINGHKQESLAVSDRATQFGDGCFTTARVIDGKVSLLSAHIQRLQDACQRLMISCDFWPQLEQEMKTL---AAEQQNGVLKVVISRGSGGRGYST
 S_enterica_CT18 ---MFLINGHAQDQLAVSDRATQFGDGSFTTARIVDGNICHLEAHLQRLQVACEKLRIAFSHWSTLRQEMTML---ATGHDSGVLKVIISRGSGGRGYSA
  S_enterica_Ty2 ---MFLINGHAQDQLAVSDRATQFGDGSFTTARIVDGNICHLEAHLQRLQVACEKLRIAFSHWSTLRQEMTML---ATGHDSGVLKVIISRGSGGRGYSA
        S_boydii ---MFLINGHKQESLAVSDRATQFGDGCFTTARVIDGKVSLLSAHIQRLQDACQRLMIFCDFWPQLEQEMKTL---AAEQQNGVLKVVISRGSGGRGYST
   S_dysenteriae ---MFLINGYKQESLAVSDRATQFGDGCFTTARVIDGKVSLLSAHIQRLQDACQRLMISCDFWPQLEQEMKTL---AAEQQNGVLKVVISRGSGGRGYST
   P_luminescens ---MYWINGERCDYLPANDRSVQFGDGCFTTAKIEQGQVALLSLHIQRLQKGVERLYLPQPDWQRLESHIRQI---ASTTEQGVLKVIISRGTGGRGYSA
        Y_pestis ---MFWINGVEQNLISASDRSVQFGDGCFTTARVSDGRVVWLDQHVLRLQRATERLLLPTVNWKILTKEMVEA---ASHTESGVLKVIISRGSGGRGYSG
  A_ferrooxidans MDGDPLVAGACDATL---DRALHYGDGLFETIAVIRGVPLFWEEHLARLERGATILNIPAPHPEVWREDLRTALDAIPAQPRLLLKLTLGRGP-GPGYGS
           ruler 1.......10........20........30........40........50........60........70........80........90.......100

                 ----------------------------------------------------------------------------------------------------
      E_coli_K12 LNSGPATRILSVTAYPAHYDRLRNEGITLALSPVRLGRNPHLAGIKHLNRLEQVLIRSHLEQTNADEALVLDSEGWVTECCAANLFWRKGNVVYTPRLDQ
   E_coli_CFT073 LNSGPATRILSITAYPAHYDRLRNEGMMLALSPVRLGRNPHLAGIKHLNRLEQVLIRSHLEQTNADEALVLDSEGWVTECCAANLFWRKGNVVYTPRLDQ
 S_enterica_CT18 MNCQAATRILSVSAYPAYYSQWRKQGITLTLSPIPLGRNPYLAGLKHLNRLEQVLIRSHLEQTDADEALVLDSEGWVTECCAANLFWRTGDIVFTPRLDQ
  S_enterica_Ty2 MNCQAATRILSVSAYPAYYSQWRKQGITLTLSPIPLGRNPYLAGLKHLNRLEQVLIRSHLEQTDADEALVLDSEGWVTECCAANLFWRTGDIVFTPRLDQ
        S_boydii LNSGPATRILSVTVYPAHYDRLRNEGMTLALSPVRLGRNPHLAGIKHLNRLEQVLIRSHLEQTNADEALVLDSEGWVTECCAANLFWRKGNVVYTPRLDQ
   S_dysenteriae LNSGPATRILSVTAYPAHYDRLRNEGMTLALSPVRLGRNPHLAGIKHLNRLEQVLIRSHLEQTNADEALVLDSEGWVTECCAANLFWRKGNVVYTPRLDQ
   P_luminescens QGFTQPNQILSLSPYPDKYLKQRQKGLSMVLSPVAMGINPYLAGIKHLNRLEQILIKTFIEKSNADEALVLDTDGLLVECCTANIFWRKGKGVYTPDLRG
        Y_pestis TACQHPTRIISLSDYPAHYSTWRDRGISLALSPVPLARNPLLAGIKHLNRLEQVLIRAHLEQTEADEAVVLDTAGQLVECCAANLFWRKGNQVCTPDLSH
  A_ferrooxidans AGAGPPRRYLWLSRWPERNPHYWNPGISAAACEVALLTGAPYLGVKSLNRLNQVMARDALAPEYA-EGVMMDQSGLLREGIMSNLFWVMTGVVHTPELEN
           ruler .......110.......120.......130.......140.......150.......160.......170.......180.......190.......200

                 ----------------------------------------------------------------------------------
      E_coli_K12 AGVNGIMRQFCIRLLAQSSYQLVEVQASLEESLQADEMVICNALMPVMPVCAC------GDVSFSSATLYEYLAPLCERPN-
   E_coli_CFT073 AGVNGIMRQFCIRLLAQSSYQLVEVQASLEEALQADEMVICNALMPVMPVRAC------GDVSFRQQRYMNI----------
 S_enterica_CT18 AGVNGIMRQFCLRQLAQSPFQVLEVQAREEAVRQADEIIICNALMPIIPIRAY------HGTSYSSRTLFQFLAPFCEHPN-
  S_enterica_Ty2 AGVNGIMRQFCLRQLAQSPFQVLEVQAREEAVRQADEIIICNALMPIIPIRAY------HGTSYSSRTLFQFLAPFCEHPN-
        S_boydii AGVNGIMRQFCIRLLAQSSYQLVEVQASLEEALQADEMVICNALMPVMPVRAC------GDVSFSSATLYEYLAPLCERPN-
   S_dysenteriae AGVNGIMRQFCIRLLAQSSYQLVEVQASLEEALQADEMVICNALMPVMPVRAC------GDVSFSSATLYEYLAPLCERPN-
   P_luminescens CGVEGVMRERIIALLSESDYHFSCVSCYPETLADADEVIVCNSLMPVIAVKEIQAHENQPRWQYQSRELYEYLLPECLKL--
        Y_pestis SGVDGIMRQRIIACLAAHNMPVEIVVQPLNVLADADEVIVCNALMPVLPVNKV------GKWVYKSRVLFDLLNLHCCDI--
  A_ferrooxidans GGIAGVQRAAILAWLQAHGVPTRLGHWPLTVLQDADEIFFSNSLIGVWPVRYFMGRELPGHDGPITSVLLAWTREMGLGPQP
           ruler .......210.......220.......230.......240.......250.......260.......270.......280..
```

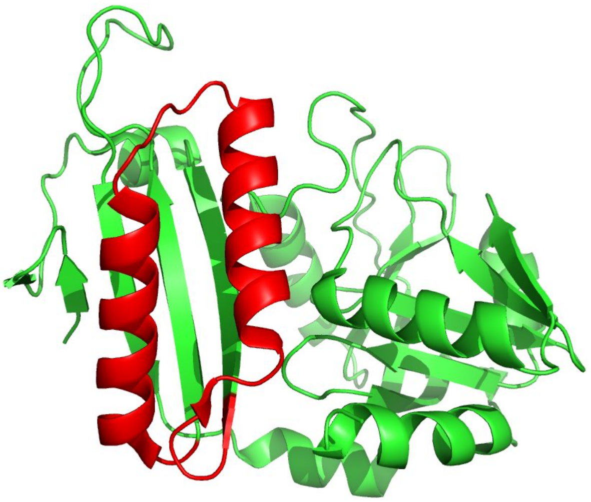

Figure S1.11: Sequence alignment and tertiary structure for gene *ycfH*. The mutation cluster is highlighted in red.

E. coli K12 entry: b1100; PDB ID: 1yix; chain ID: A

```
                --------------------------------------------------------------------------------------------XXXXXXXX
     E_coli_K12 --------------------MFLVDSHCHLDGLDYESLHKDVDDVLAKAAARDVKFCLAVATTLPGYLHMRDLVGERDNVVFSCGVHPLNQ-NDPYDVED
  E_coli_CFT073 LSITCNRALCYRFLIFKRDIMFLVDSHCHLDGLDYESLHKDVDDVLAKAAARDVKFCLAVATTLPGYLHMRDLVGERDNVVFSCGVHPLNQ-NDPYDVED
S_enterica_CT18 --------------------MFLVDSHCHLDGLDYQSLHKDVDDVLAKAAARDVKFCLAVATTLPGYRHMRELVGKRDNVVFSCGVHPLNQ-DEVYDVEE
 S_enterica_Ty2 --------------------MFLVDSHCHLDGLDYQSLHKDVDDVLAKAAARDVKFCLAVATTLPGYRHMRELVGKRDNVVFSCGVHPLNQ-DEVYDVEE
       S_boydii --------------------MFLVDSHCHLDGLDYESLHKDVDDVLAKAAARDVKFCLAVATTLPGYLHMRDLVGERDNVVFSCGVHPLNQ-NDPYDVED
  S_dysenteriae --------------------MFLVDSHCHLDGLDYESLHKDVDDVLAKAAARDVKFCLAVATTLPGYLHMRDLVGERDNVVFSCGVHPLNQ-NDPYDVED
  P_luminescens --------------------MLLVDSHCHLDCLDYENLHKNVDDVVAKAADREVKFMLAVATTLPGFQQMKTLIGERENIVFSCGIHPLNL-DEGYNFDE
       Y_pestis --------------------MLLVDSHCHLDSLDYQTLHSSVDDVLAKANARDVGFILAVATTLPGFTAMTALIGERKNVAFSCGVHPLNL-DGGYDFQQ
   B_aphidicola --------------------MFLIDSHCHLDLLNYNKIHTGIQDVLNKSKKKHVNVILTVSTSIENFCYLLKFIKGNRNVLLSCGIHPHYIPENKNEILK
 A_ferrooxidans ---------------------MLVDSHCHLDFEDFDA---DRSEILARARAAGVGEMLIAAVVEAHWPRVQALAAGHAGVWAAVGVHPNEPAGATPEWER
  L_pneumophila ---------------------VLVDSHCHLNFIDLTHFHHDLAQVINQARENEVEHFLSVCVELSDYPQLERLAKDYPDISISVGVHPNSEMNYPVTAQM
          ruler 1.......10........20........30........40........50........60........70........80........90.......100

                XXXXXXXXXXXXXXXXXXXXX-------------------------------------------------------------------------------
     E_coli_K12 LRRLAAEEGVVALGETGLDYYYTP--ETKVRQQESFIHHIQIGRELNKPVIVHTRDARADTLAILREEKVTDCGGVLHCFTEDRETAGKLLDLGFYISFS
  E_coli_CFT073 LRRLAAEEGVVALGETGLDYYYTP--ETKVRQQESFIHHIQIGRELNKPVIVHTRDARADTLAILREEKVTDCGGVLHCFTEDRETAGKLLDLGFYISFS
S_enterica_CT18 LRLLAAEDDVVAMGETGLDYFYTP--ETKIRQQASFIHHIQIGRELHKPVIVHTRDARADTLAILREEKVTDCGGVLHCFTEDRETAGKLLDLGFYISFS
 S_enterica_Ty2 LRLLAAEDDVVAMGETGLDYFYTP--ETKIRQQASFIHHIQIGRELHKPVIVHTRDARADTLAILREEKVTDCGGVLHCFTEDRETAGKLLDLGFYISFS
       S_boydii LRRLAAEEGVEPLCETGLDYYYTP--ETKVLQRESFIHHIQIARELNKPVIVHTRDARADTLAILREEKVTDCGGVLHCFTEDRETAGKLLDLGFYISFS
  S_dysenteriae LRRLAAEEGVVALGETGLDYYYTP--ETKVRQQESFIHHIQIGRELNKPVIVHTRDARADTLAILREEKVTDCGGVLHCFTEDRETAGKLLDLGFYISFS
  P_luminescens LARLASGNEVVALGETGLDYYYQQ--DNAELQRTSFREHIRIGCKLNKPVIVHTRSARDDTLRVLREEQARECGGVLHCFTEDKDTARELLDLGFYISFS
       Y_pestis LRELAAGTHVVAMGETGLDYFYQQ--DNIPLQQASFREHIRIGRELNKPVIVHTRDAREDTLSILREEQAQDCGGVLHCFTEDKATAATLLDLGFYISFS
   B_aphidicola LKKYSNNEKVVAIGETGLDYYRNN--DNKKLQQILFREHIKTSITLKKPLLIHTRNSINDTINILKEENSKQCIGVLHSFTEDMHSARILLNMGFYISFS
 A_ferrooxidans LLVALAADKVVAVGETGLDYYRSE--GDLSWQRERFAQHIAAAKATGKPLIVHTRAAAADTLAMLRSEDAAAAGGVIHCFTENRDFARTALDMGFYISFS
  L_pneumophila LCELAQNPACIAIGETGLDYYRNPTEEAQSLQRDRFREHIKAALITSKPLIIHTRQAAEDTLLMMAKEGASQIGGVMHCFAEDLDIAQRAIDLNFYISFS
          ruler .......110.......120.......130.......140.......150.......160.......170.......180.......190.......200

                --------------------------------------------------------------------------------------------
     E_coli_K12 GIVTFRNAEQLRDAARYVPLDRLLVETDSPYLAPVPHRGKENQPAMVRDVAEYMAVLKGVAVEELAQVTTDNFARLFHIDASRLQSIR----
  E_coli_CFT073 GIVTFRNAEQLRDAARYVPLDRLLVETDSPYLAPVPHRGKENQPAMVRDVAEYMAVLKGVAVEELAQVTTDNFARLFHIDASRLQSIR----
S_enterica_CT18 GIVTFRNAEQLRDAARYVPLDRLLVETDSPYLAPVPHRGKENQPAMVRDVAEYMAVLKGVAVEELAQITTDNFARLFHIDASRLSSIR----
 S_enterica_Ty2 GIVTFRNAEQLRDAARYVPLDRLLVETDSPYLAPVPHRGKENQPAMVRDVAEYMAVLKGVAVEELAQITTDNFARLFHIDASRLSSIR----
       S_boydii GIVTFRNAEQLRDAARYVPLDRLLVETDSPYLAPVPHRGKENQPAMVRDVAEYMAVLKGVAVEELAQVTTDNFARLFHIDASRLQSIR----
  S_dysenteriae GIVTFRNAEQLRDAARYVPLDRLLVETDSPYLAPVPHRGKENQPAMVRDVAEYMAVLKGVAVEELAQVTTDNFARLFHIDASRLQSIR----
  P_luminescens GIVTFRNAEQIREAARYVPLDRILIETDSPYLAPVPHRGKENQPAYVRDVAEYMAVLKGVSIEALAEVTTQNFCDLFHVDVDKI--------
       Y_pestis GIVTFRNAEQLRDVARYVPLDRLLVETDSPYLAPVPHRGKENQPAYVRDVAEYMAVLKGVSLESLAEATTANFCRLFHLDPSTLSSEKRPLP
   B_aphidicola GIVTFKNSKIVHETAKFVPIDRILIETDSPYLSPVPYRGIENQPAYLYDTMLYIAQLKNMSPECFAIQTTKNFLKLFNLPSYFTNMS-----
 A_ferrooxidans GIVTFRKSVELQAVAKMLPADRLLVETDAPYLAPEPQRGRRNEPAFVAHVAAFLAALRGEAPEAVATQTTANFHALFRHAVAS---------
  L_pneumophila GIVTFKNAVSLQDVARQIPLERILIETDSPYLAPVPHRGKQNHPALVKHVAEAIAQLRGMDFQEIAEITTNNFYNCFNIKRQG---------
          ruler .......210.......220.......230.......240.......250.......260.......270.......280.......290..
```

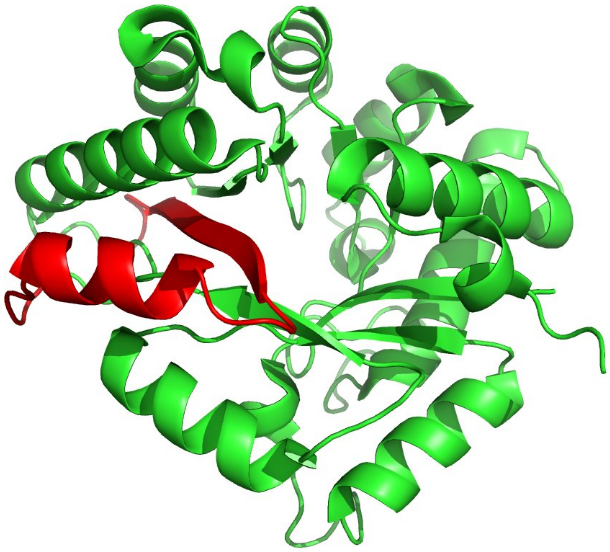

Figure S1.12: Sequence alignment and tertiary structure for gene *narH*. The mutation cluster is highlighted in red.

E. coli K12 entry: b1225; PDB ID: 1q16; chain ID: B

```
                  ----------------------------------------------------------------------------------------------------
       E_coli_K12 MKIRSQVGMVLNLDKCIGCHTCSVTCKNVWTSREGVEYAWFNNVETKPGQGFPTDWENQEKYKGGWIRKINGKLQPRMGNRAMLLGKIFANPHLPGIDDY
    E_coli_CFT073 MKIRSQVGMVLNLDKCIGCHTCSVTCKNVWTSREGVEYAWFNNVETKPGQGFPTDWENQEKYKGGWIRKINGKLQPRMGNRAMLLGKIFANPHLPGIDDY
S_enterica_CT18 MKIRSQVGMVLNLDKCIGCHTCSVTCKNVWTSREGMEYAWFNNVESKPGVGFPNDWENQEKWKGGWIRKINGKLQPRMGNRALLLGKIFANPHLPGIDDY
 S_enterica_Ty2 MKIRSQVGMVLNLDKCIGCHTCSVTCKNVWTSREGMEYAWFNNVESKPGVGFPNDWENQEKWKGGWIRKINGKLQPRMGNRALLLGKIFANPHLPGIDDY
        S_boydii MKIRSQVGMVLNLDKCIGCHTCSVTCKNVWTSREGVEYAWFNNVETKPGQGFPTDWENQEKYKGGWIRKINGKLQPRMGNRAMLLGKIFANPHLPGIDDY
   S_dysenteriae MKIRSQVGMVLNLDKCIGCHTCSVTCKNVWTSREGVEYAWFNNVETKPGQGFPTDWENQEKYKGGWIRKINGKLQPRMGNRAMLLGKIFANPHLPGIDDY
            ruler 1.......10........20........30........40........50........60........70........80........90.......100

                  --------------------------------XXXXXXXXXXXXXXXXXXXXXXXXXXXXXXXX------------------------------------
       E_coli_K12 YEPFDFDYQNLHTAPEGSKSQPIARPRSLITGERMAKIEKGPNWEDDLGGEFDKLAKDKNFDNIQKAMYSQFENTFMMYLPRLCEHCLNPACVATCPSGA
    E_coli_CFT073 YEPFDFDYQNLHTAPEGSKSQPIARPRSLITGERMAKIEKGPNWEDDLGGEFDKLAKDKNFDNIQKAMYSQFENTFMMYLPRLCEHCLNPACVATCPSGA
S_enterica_CT18 YEPFDYDYQNLHTAPE-SKHQPIARPRSLITGQRMDKITSGPNWEEILGGEFEKRAKDQNFENMQKAMYGQFENTFMMYLPRLCEHCLNPACVATCPSGA
 S_enterica_Ty2 YEPFDYDYQNLHTAPE-SKHQPIARPRSLITGQRMDKITSGPNWEEILGGEFEKRAKDQNFENMQKAMYGQFENTFMMYLPRLCEHCLNPACVATCPSGA
        S_boydii YEPFDFDYQNLHTAPEGSKSQPIARPRSLITGERMAKIEKGPNWEDDLGGEFDKLAKDKNFDNIQKAMYSQFENTFMMYLPRLCEHCLNPACVATCPSGA
   S_dysenteriae YEPFDFDYQNLHTAPEGSKSQPIARPRSLITGERMAKIEKGPNWEDDLGGEFDKLAKDKNFDNIQKAMYSQFENTFMMYLPRLCEHCLNPACVATCPSGA
            ruler .......110.......120.......130.......140.......150.......160.......170.......180.......190.......200

                  ----------------------------------------------------------------------------------------------------
       E_coli_K12 IYKREEDGIVLIDQDKCRGWRMCITGCPYKKIYFNWKSGKSEKCIFCYPRIEAGQPTVCSETCVGRIRYLGVLLYDADAIERAASTENEKDLYQRQLDVF
    E_coli_CFT073 IYKREEDGIVLIDQDKCRGWRMCITGCPYKKIYFNWKSGKSEKCIFCYPRIEAGQPTICSETCVGRIRYLGVLLYDADAIERAASTENEKDLYQRQLDVF
S_enterica_CT18 IYKREEDGIVLIDQDKCRGWRMCITGCPYKKIYFNWKSGKSEKCIFCYPRIEAGQPTVCSETCVGRIRYLGVLLYDADAIESAASTENEKDLYQRQLDVF
 S_enterica_Ty2 IYKREEDGIVLIDQDKCRGWRMCITGCPYKKIYFNWKSGKSEKCIFCYPRIEAGQPTVCSETCVGRIRYLGVLLYDADAIESAASTENEKDLYQRQLDVF
        S_boydii IYKREEDGIVLIDQDKCRGWRMCITGCPYKKIYFNWKSGKSEKCIFCYPRIEAGQPTVCSETCVGRIRYLGVLLYDADAIERAASTENEKDLYQRQLEVF
   S_dysenteriae IYKREEDGIVLIDQDKCRGWRMCITGCPYKKIYFNWKSGKSEKCIFCYSRIEAGQPTVCSETCVGRIRYLGVLLYDADAIERAASTENEKDLYQRQLEVF
            ruler .......210.......220.......230.......240.......250.......260.......270.......280.......290.......300
```

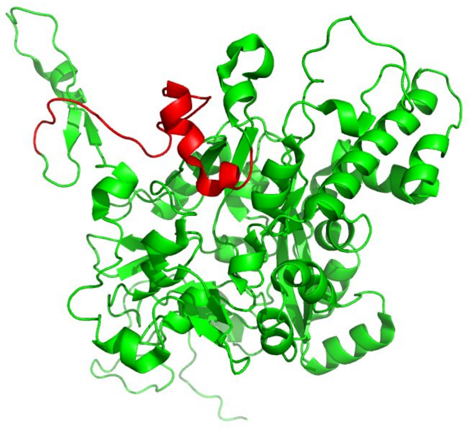

Figure S1.13: Sequence alignment and tertiary structure for gene *trpE*. The mutation cluster is highlighted in red.

E. coli K12 entry: b1264; PDB ID: 1i1q; chain ID: A

```
               ----------------------------------------------------------------------------------------------------
     E_coli_K12 ----------MQTQKPTLELLTCEGAYRDNPTALFHQLCGDRPATLLLESADIDSKDDLKSLLLVDSALRITALGDTVTIQALSGNGEALLALLDNALPA
  E_coli_CFT073 ----------MQTQKPTLELLTCEGAYRDNPTALFHQLCGDRPATLLLESADIDSKDDLKSLLLVDSALRITALSDTVTIQALSGNGEALLTLLDNALPA
S_enterica_CT18 ----------MQTPKPTLELLTCDAAYRENPTALFHQVCGDRPATLLLESADIDSKDDLKSLLLVDSALRITALGDTVTIQALSDNGASLLPLLDTALPA
 S_enterica_Ty2 ----------MQTPKPTLELLTCDAAYRENPTALFHQVCGDRPATLLLESADIDSKDDLKSLLLVDSALRITALGDTVTIQALSDNGASLLPLLDTALPA
       S_boydii ----------MQTQKPTLELLTCEGAYRDNPTALFHQLCGDRPATLLLESADIDSKDDLKSLLLVDSALRITALGDTVTIQALSGNGEALLTLLDNALPA
  S_dysenteriae ----------MQTQKPTLELLTCEGAYRDNPTALFHQLCGDRPATLLLESADIDSKDDLKSLLLVDSALRITALGDTVTIKALSGNGEALLALLDNALPA
  P_luminescens ----------MTDKQPKITVIEQEAAYQPDPTQLFNQLCGERPATLLLESAEINSKKNLKSLLIIDSALRISANGNQVTFDALTENGRNLLPILANKFSS
       Y_pestis ---------MMQTSRPTLQLLTASACYRDDPTALFHQLCGARPATLLLESAEVDNKQNLKSLLVIDSALRITALGQTVTLEALTRNGASLLPLLDASLPT
   B_aphidicola ----------MKKNLISIDVFLTETRYQPNPTAIFNQICKKKSETLLLESAEINKKHHLESMMIIDAALKISFLNQIVIVEALTKNGVNLLSVFKSLLPK
 A_ferrooxidans VIPKATFEALARQGYNRIPLSREVIADLDTPLSAYLKLV-DGPYAYLLESVQGGEKWGRYSIIGLPAQQILRVTQGVVTVHV-----------------D
  L_pneumophila --MVFMLQQYKTLGGVHVEFSQQELDYQQGIDFLLES-LDSQRGAVFASSFEYPGRYTCWDMGFYNPPLVMICQSNTICFEALNQRGEILLSFLYPLLKT
          ruler 1.......10........20........30........40........50........60........70........80........90.......100

               ----------------------------------------------XXXXXXX----XXXXXXXXXXX---XXXXXXXXXXX----XXXXXXXXXX-XXX
     E_coli_K12 ----GVESEQSPNCRVLRFPPVSPLLDEDARLCSLSVFDAFRLLQ-NLLNVPK----EEREAMFFGGL---FSYDLVAGFED----LPQLSAENNC-PDF
  E_coli_CFT073 ----GVENEQSPNCRVLRFPPVSPLLDEDARLCSLSVFDAFRLLQ-NLLNVPK----EEREAMFFGGL---FSYDLVAGFEN----LPQLSAENSC-PDF
S_enterica_CT18 ----GVENEVLPAGRILRFPPVSPLLDEDARLCSLSVFDAFRLLQ-GVVNIPT----QEREAMFFGGL---FAYDLVAGFEA----LPHLEAGNNC-PDY
 S_enterica_Ty2 ----GVENEVLPAGRILRFPPVSPLLDEDARLCSLSVFDAFRLLQ-GVVNIPT----QEREAMFFGGL---FAYDLVAGFEA----LPHLEAGNNC-PDY
       S_boydii ----GVENEQSPNCRVLRFPPVSPLLDEDARLCSLSVFDAFRLLQ-NLLNVPK----EEREAMFFGGL---FSYDLVAGFED----LPQLSAENNC-PDF
  S_dysenteriae ----GVENEQSPNCRVLRFPPVSPLLDEDARLCSLSVFDAFRLLQ-NLLNVPK----EEREAMFFGGL---FSYDLVAGFED----LPQLSAENNC-PDF
  P_luminescens ----DVQSEITHNALQVIFPPINHNIDEDNRLKAVSVFDALRALL-SLAEEPSPYNSSSAEAIFVGGL---FSYDLVAGFEP----LPAVKTVQRC-PDF
       Y_pestis ----EVDIQVRPNGRELTFPLINEVQDEDSRLQSLSVFDALRQLL-TLVNTPL----AEREALFLGGL---FAYDLVAGFEN----LPPLRQDQRC-PDF
   B_aphidicola ----NVIILSDNNPLEIKFPILSMYLDEDQRLRSLSVFDAIRFLIKSVKNLSE----FAPKSMFFGGL---FSYDLITSFEN----LPILNTHQHC-PDF
 A_ferrooxidans ----GVETE--------RATPADP-------LEWISVFRR------RFRVAPL----ENADERFSGGLVGYFGYDIVRSIEPRLARSTPLPDPLGL-PEI
  L_pneumophila TDLWEVISQRTSFC-QLKIKSSDKVFSEEERSHQPSVFSAIRLLL-NF---------FKTEEDSYLGLYGAFGFDLIYQFEQ----LEQCKPRNETQRDM
          ruler .......110.......120.......130.......140.......150.......160.......170.......180.......190.......200

               XXXXXXXXXXXXXXXXXXXXXXXXXXXXXXXXXXXXXXXXXXXXXXXXXXXX-XXXXXXXXXX---XXXXXXXXXXXXXXXX------------------
     E_coli_K12 CFYLAETLMVIDHQKKSTRIQASLFAPNEEEKQRLTARLNELRQQLTEAAPP-LPVVSVPHMR---CECNQSDEEFGGVVRLLQKAIRAGEIFQVVPSRR
  E_coli_CFT073 CFYLAETLMVIDHQKKSTRIQASLFAPNEEEKQRLTARLNELRQQLTEAAPP-LPVVSVPHMR---CECNQSDEEFGGVVRLLQKAIRAGEIFQVVPSRR
S_enterica_CT18 CFYLAETLMVIDHQKKSTRIQASLFTASDLEKQRLNARLAYLSQQLTQPAPP-LPVTPVPDMR---CECNQSDDAFGAVVRQLQKAIRAGEIFQVVPSRR
 S_enterica_Ty2 CFYLAETLMVIDHQKKSTRIQASLFTASDLEKQRLNARLAYLSQQLTQPAPP-LPVTPVPDMR---CECNQSDDAFGAVVRQLQKAIRAGEIFQVVPSRR
       S_boydii CFYLAETLMVIDHQKKNTRIQASLFAPNEEEKQRLTARLNDLRQQLTEAAPP-LPVVSVPHMR---CECNQSDEEFGGVVRLLQKAIRTGEIFQVVPSRR
  S_dysenteriae CFYLAETLMVIDHQKKSTRIQASLFAPNEEEKQRLTARLNELRQQLTEAAPP-LPVVSVPHMR---CECNQSNEEFGGVVRSLQKAIRAGEIFQVVPSRR
  P_luminescens CFYLAETLLVINHQNKNARLQISLFSDSQAEEQRLKQRLKTLVALITEPIEP-VQQISLPTMK---VSYNRDDKSYAEMVAQLQQYIRQGDIFQVVPSRR
       Y_pestis CFYLAETLLVLDHQHRSTRLQASLFTPDSSEYQRLATRLEQLSHQLQQAPHP-IPATSVPEMA---LQCNQSDEEYCNVVSELQVAIREGEIFQVVPSRR
   B_aphidicola CFYLSETLLILDHKNKTSIVQVTSFTNDNFEKKRLKDRLEILKNKLSKNLCP-IKFDTLKNMK---LWCNKTDEEYNKIIVNVKKFILQGEIFQVVPSRK
 A_ferrooxidans QLLVADELLIFDNLRGTLRLLVHADPAQPEAWERGAARLRELQQRLRESVPPSSPPVSTRHVREEDFTASFSREGYMAAVRTIKAYIGAGDVMQVVPSQR
  L_pneumophila VLYLPDEIYIVNHRKEEAFVRRYDFQYQGRSTQSLSREGKY---------SP-YQPIHKPEKN-----CDHEPGEYAEVVKKAKEKFSCGDLFEVVPSQT
          ruler .......210.......220.......230.......240.......250.......260.......270.......280.......290.......300
```

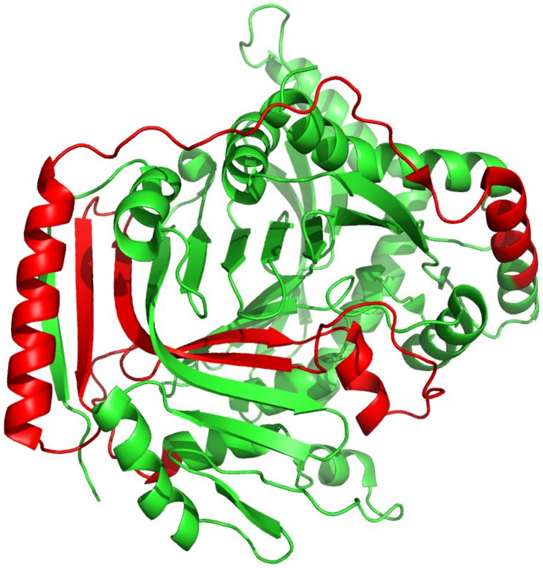

Figure S1.14: Sequence alignment and tertiary structure for gene *katE*. The mutation cluster is highlighted in red.

E. coli K12 entry: b1732; PDB ID: 1qf7; chain ID: A

```
                  ----------------------------------------------------------------------------------------------------
       E_coli_K12 KLIPEELVPVQRVGKMVLNRNPDNFFAENEQAAFHPGHIVPGLDFTNDPLLQGRLFSYTDTQISRLGGPNFHEIPINRPTCPYHNFQRDGMHRMGIDT-N
    E_coli_CFT073 KLIPEELVPVQRVGKMVLNRNPDNFFAENEQVAFHPGHIVPGLDFTNDPLLQGRLFSYTDTQISRLGGPNFHEIPINRPTCPYHNFQRDGMHRMGIDT-N
  S_enterica_CT18 KLIPEELVPVQRVGKMVLNRNPDNFFAENEQAAFHPGHIVPGIDFTNDPLLQGRLFSYTDTQISRLGGPNFHEIPINRPTCPYHNFQRDGMHRMDIDT-N
   S_enterica_Ty2 KLIPEELVPVQRVGKMVLNRNPDNFFAENEQAAFHPGHIVPGIDFTNDPLLQGRLFSYTDTQISRLGGPNFHEIPINRPTCPYHNFQRDGMHRMDIDT-N
    P_luminescens KVWPHGDYPMIDVGYFELNRNPDNYFSDVEQAAFNPANVVPGISFSPDKMLQGRLFSYGDAQRYRLG-VNHHQIPVNTPRCPFHNYHRDGAMRVDGNSGN
        Y_pestis KVWPHGDYPLIDVGYFELNRNPENYFAEVEQSSFNPANVVPGISFSPDRMLQGRLFSYGDAARYRLG-VNHHQIPVNSAKCPFHNYHRDGAMRVDGNSGN
            ruler 360.......370.......380.......390.......400.......410.......420.......430.......440.......450.......

                  ----------------------------------------------------------------XXXXXXXXXXXXXXXXXXXXXXXXXXXXXXXXXXXX
       E_coli_K12 PANYEPNS---INDNWPRETPPGPKRGGFESYQERVEGNKVRERSPSFGEYYSHPRLFWLSQTPFEQRHIVDGFSFELSKVVRPYIRERVVDQLAHIDLT
    E_coli_CFT073 PANYEPNS---INDNWPRETPPGPKRGGFESYQERVEGNKVRERSPSFGEYYSHPRLFWLSQTPFEQHHIVDGFSFELSKVVRPYIRERVVDQLAHIDLT
  S_enterica_CT18 PANYEPNS---INDNWPRETPPAPKRGGFESYQERVDGNEIRERSPSFGEYYSHPRLFWLSQTPIEQQHIIDAFSFELGKVARAYIRERVVDQLAHIDVT
   S_enterica_Ty2 PANYEPNS---INDNWPRETPPAPKRGGFESYQERVDGNEIRERSPSFGEYYSHPRLFWLSQTPIEQQHIIDAFSFELGKVARAYIRERVVDQLAHIDVT
    P_luminescens TVTYEPNNAGLFQEQPEFKHPSLSLEGDADHWNHRQDE-----------DYFSQPRKLFMLINSKEHQRMFDRIASELSQAHED-TQRRQVELFRKVHPE
        Y_pestis GATYEPNSFGLFQEQPDFSEPPLTLEGAADHWNHREDT-----------DYFSQPRALFNLLSEEEHQRMFTRIAGELSQVPEA-IQRRQIALFTQVDPA
            ruler 460.......470.......480.......490.......500.......510.......520.......530.......540.......550.......

                  XXXXXXXXXXXXXXXXXXXXXXXXXXXXXXXXXXXXXXXXXXXXXXXXXXXXXXXXXXXXXXXXXXXXXXXXXXXXXXXXXXXXXXXXXXXXXXXXXXXX
       E_coli_K12 LAQAVAKNLGIELTDDQLNITPPPDVNGLKKDPSLSLYAIPDGDVKGRVVAILLNDEVRSADLLAILKALKAKGVHAKLLYSRMGEVTADDGTVLPIAAT
    E_coli_CFT073 LAQAVAKNLGIELTDDQLNITPPPDVNGLKKDPSLSLYAIPDGDVKGRVVAILLNDEVRSADLLAILKALKAKGVHAKLLYSRMGEVTADDGTVLPIAAT
  S_enterica_CT18 LAQGVAHNLGFALTHEQTQIAPPPDVNGLKKDPALSLYAVPDGDVKGRVVAILLNDKVNAAELLTIFQALKAKGVHVKLLYSRMGEVTADDGSTLTIAAT
   S_enterica_Ty2 LAQGVAHNLGFALTHEQTQIAPPPDVNGLKKDPALSLYAVPDGDVKGRVVAILLNDKVNAAELLTIFQALKAKGVHVKLLYSRMGEVTADDGSTLTIAAT
    P_luminescens YGAGVENAL-------------------------------------------------------------------------------------------
        Y_pestis YGAGVKKALGLN----------------------------------------------------------------------------------------
            ruler 560.......570.......580.......590.......600.......610.......620.......630.......640.......650.......

                  XXXXXXXXXXXXXXXXXXXXXXXXXXXXXXXXXXXXXXXXXXXXXXXXXXXXXXXXXXXXXXXXXXXXXXXXXXXXXXXXXXXXXXXXXXXXXXXXX---
       E_coli_K12 FAGAPSLTVDAVIVPCGNIADIADNGDANYYLMEAYKHLKPIALAGDARKFKATIKIADQGEEGIVEADSADGSFMDELLTLMAAHRVWSRIPKIDKIPA
    E_coli_CFT073 FAGAPSLTVDAVIVPCGNIADIANNGDANYYLMEAYKHLKPIALAGDARKFKATIKVADQGEEGIAEADSADGSFMDELLTLMTAHRVWSRIPKIDKIPA
  S_enterica_CT18 FAGAPSLTVDAVIVPCGNIADIESCGDARYYLLEAYKHLKPIALAGDARRFKALLNIDSQGEEGLVEADNVDHHFMDTLLTLMAAHRVWSRAGKINAIPA
   S_enterica_Ty2 FAGAPSLTVDAVIVPCGNIADIESCGDARYYLLEAYKHLKPIALAGDARRFKALLNIDSQGEEGLVEADNVDHHFMDTLLTLMAAHRVWSRAGKINAIPA
    P_luminescens -----------------------------------------------------------------------------------------------KKIKG
        Y_pestis ----------------------------------------------------------------------------------------------------
            ruler 660.......670.......680.......690.......700.......710.......720.......730.......740.......750.......
```

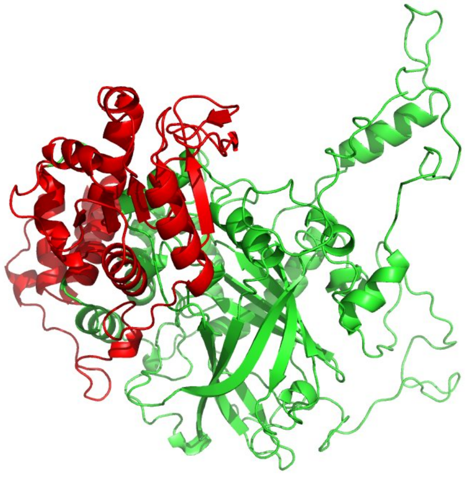

Figure S1.15: Sequence alignment and tertiary structure for gene *rnd*. The mutation cluster is highlighted in red.

E. coli K12 entry: b1804; PDB ID: 1yt3; chain ID: A

```
                ----------------------------------------------------------------------------------------------------
     E_coli_K12 RDPSITKFLHAGSEDLEVFLNVFGELPQPLIDTQILAAFCGRPMSWGFASMVEEYSGVTLDKSESRTDWLARPLTERQCEYAAADVWYLLPITAKLMVET
  E_coli_CFT073 RDPSITKFLHAGSEDLEVFLNVFGELPQPLIDTQILAAFCGRPMSWGFASMVEEYSGVTLDKSESRTDWLARPLTERQCEYAAADVWYLLPITAKLMVET
S_enterica_CT18 RDTGITKFLHAGSEDLEVFLNAFGELPEPLIDTQILAAFCGRPLSWGFASMVEEYTGVALDKSESRTDWLARPLSERQCEYAAADVWYLLPIAKKLMIET
 S_enterica_Ty2 RDTGITKFLHAGSEDLEVFLNAFGELPEPLIDTQILAAFCGRPLSWGFASMVEEYTGVALDKSESRTDWLARPLSERQCEYAAADVWYLLPIAKKLMIET
       S_boydii RDPSITKFLHAGSEDLEVFLNVFGELPQPLIDTQILSAFCGRPMSWGFASMVEEYSGVTLDKSESRTDWLARPLTERQCEYAAADVWYLLPITAKLMVET
  S_dysenteriae RDPSITKFLHAGSEDLEVFLNVFGELPQPLIDTQILAAFCGRPMSWGFASMVEEYSGVTLDKSESRTDWLARPLTERQCEYAAADVWYLLPITAKLMVET
  P_luminescens THPQILKLLHAGSEDLEVFLNAFQCLPEPMIDTQVLAAFIGHPLSCGFAALVAEYIHVELDKSESRTDWLARPLSEKQCEYAAADVYYLLPLADILMTAT
       Y_pestis QDLNVVKYLHAGSEDLEVFLNAFDQMPTPMIDTQVLAAFSGRSLSCGFAMLVAEFEGVELDKSESRTDWIARPLSEKQCDYAAADVFYLLPLAAKLVEAT
          ruler ........80........90.......100.......110.......120.......130.......140.......150.......160.......170

                ----------------------------------------------------------------------------------------------------
     E_coli_K12 EASGWLPAALDECRLMQMRRQEVVAPEDAWRDITNAWQLRTRQLACLQLLADWRLRKARERDLAVNFVVREEHLWSVARYMPGSLGELDSLGLSGSEIRF
  E_coli_CFT073 EASGWLPAALDECRLMQMRRQEVVAPEDAWRDITNAWQLRTRQLACLQLLADWRLRKARERDLAVNFVVREEHLWSVARYMPGSLGELDSLGLSGSEIRF
S_enterica_CT18 EAAGWLPAALDECRLMQQRRQEIQAPEEAWRDITNAWQLRTRQLACLQLLADWRLRKARERDMAVNFVVREENLWAVARYMPGSLGELDSLGLSGSEIRF
 S_enterica_Ty2 EAAGWLPAALDECRLMQQRRQEIQAPEEAWRDITNAWQLRTRQLACLQLLADWRLRKARERDMAVNFVVREENLWAVARYMPGSLGELDSLGLSGSEIRF
       S_boydii EASGWLPAALDECRLMQMRRQEVVAPEDAWRDITNAWQLRTRQLACLQLLADWRLRKARERDLAVNFVVREEHLWSVARYMPGSLGELDSLGLSGSEIRF
  S_dysenteriae EASGWLPAALDECRLMQMRRQEVVAPEDAWRDITNAWQLRTRQLACLQLLADWRLRKARERNLAVNFVVREEHLWSVARYMPGSLGELDSLGLSGSEIRF
  P_luminescens AQAGYMEAATGECRLISRRRKEILEPESAYKQIGNICQLRPQQLACLKKLAAWRLSQARERDLAVNFVIREENLWQVARYMPTSLAELDALGLSGQEIRC
       Y_pestis EAAGRMDAAKDECLLLCRRRSETLDPELAYREITNAWQLRGHQLACLQKLAEWRLRQARERDLAVNFVVREEHLWQVARYQPTSLGELDSLGLSGQEIRY
          ruler .......180.......190.......200.......210.......220.......230.......240.......250.......260.......270

                -----------------------------------------XXXXXXXXXXXXXX---------------------------------------------
     E_coli_K12 HGKTLLALVEKAQTLPEDALPQPMLNLMDMPGYRKAFKAIKSLITDVSETHKISAELLASRRQINQLLNWHWKLKPQNNLPELISGWRGELMAEALHNLL
  E_coli_CFT073 HGKTLLALVEKAQALPEEALPQPMLNLMDMPGYRKAFKAIKSLITDVSETHKISAELLASRRQINQLLNWHWKLKPQNNLPELISGWRGELMAEALHNLL
S_enterica_CT18 HGKTLISLVAKAQALPEEALPEPLLNLMDMPGYRKAFKAIKALVAEVSASHHVSGELLASRRQINQLLNWHWKLKPQNGQPELISGWRAELMAEKLTLLL
 S_enterica_Ty2 HGKTLISLVAKAQALPEEALPEPLLNLMDMPGYRKAFKAIKALVAEVSASHHVSGELLASRRQINQLLNWHWKLKPQNGQPELISGWRAELMAEKLTLLL
       S_boydii HGKTLLTLVEKAQTLPEEALPQPMLNLMDMPGYRKAFKAIKSLITDVSETHKISAELLASRRQINQLLNWHWKLKPQNNLPELISGWRGELMAEALHNLL
  S_dysenteriae HGKTLLALVEKAQTLPEDALPQPMLNLMDMPGYRKAFKAIKSLITDVSETHKISAELLASRRQINQLLNWHWKLKPQNNLPELISGWRGELMAEALHNLL
  P_luminescens HGRRLLAMVAESGNIPESECPPPMTNLIDQPGYRKAFKDIKALIHQVSEQHRFNSELLASRRQINQLLSVHWNLKQLNDKPELLSGWRGELLAEPVAEIL
       Y_pestis HGRTLLALVAEGNAVPEDQLPPPIANLIDQPGYKKVFKDIKALIQSVSERSGLSSELLASRRQINQLLNWHWKLKLSDAQPELLTGWRGDLLSSELLEIL
          ruler .......280.......290.......300.......310.......320.......330.......340.......350.......360.......370
```

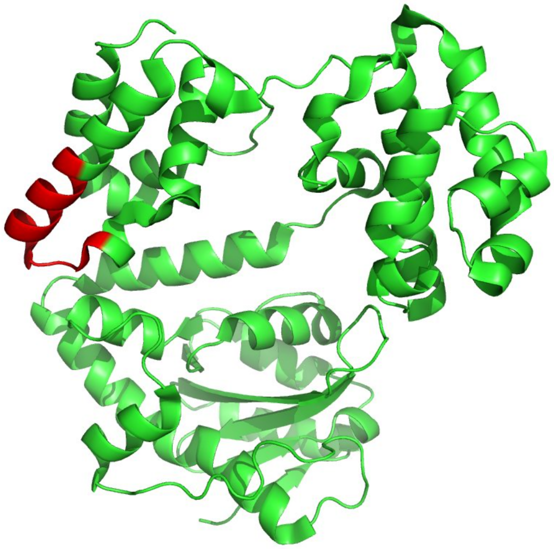

Figure S1.16: Sequence alignment and tertiary structure for gene *vsr*. The mutation cluster is highlighted in red.

E. coli K12 entry: b1960; PDB ID: 1cw0; chain ID: A

```
                 -----------------------------XXXXXXXXXXXXXXXXXXXXXXXXXXXXX------------------------------------------
      E_coli_K12 MADVHDKATRSKNMRAIATRDTAIEKRLASLLTGQGLAFRVQDASLPGRPDFVVDEYRCVIFTHGCFWHHHH-CYLFKVPATRTEFWLEKIGKNVERDRR
   E_coli_CFT073 MVDVHDKATRSKNMRAIATRDTAIEKRLASLLTGQGLAFRVQDASLPGRPDFVVDEYRCVIFTHGCFWHHHH-CYLFKVPATRTEFWLEKIGKNVERDRR
S_enterica_CT18 MADVHDKATRSKNMRAIATRDTAIEKRLAGLLSAQGITFHTQDTTLPGKPDFVVNDYDCVIFTHGCFWHHHH-CYLFKVPATRTAFWLEKIGKNVERDER
 S_enterica_Ty2 MADVHDKATRSKNMRAIATRDTAIEKRLAGLLSAQGITFHTQDTTLPGKPDFVVNDYDCVIFTHGCFWHHHH-CYLFKVPATRTAFWLEKIGKNVERDER
       S_boydii MADVHDKATRSKNMRAIATRDTAIEKRLASLLTGQGLAFRVQDASLPGSPDFVVDEYRCVIFTHGCFWHHHH-CYLFKVPATRTEFWLEKIGKNVERDRR
  S_dysenteriae MADVHDKATRSKNMRAIATRDTAIEKRLASLLTGQGLAFRVQDASLPGRPDFVVDEYLCVIFTHGCFWHHHH-CYLFKVPATRTEFWLEKIGKNVERDRR
  P_luminescens MADVHNPSTRSKNMRAIRNCDTAIEKKLVKILHDLGVTYRVQVRELPGKPDFVIDEYQAVLFVHGCFWHGHN-CHLFKTPATRTEFWLDKIEKNIRRDQF
  L_pneumophila MTDKITPEHRSRNMAAIKNKNTRPELEVRSYLFKNGFRYRLHRKDLPGKPDLTLTKYKTVIFINGCFWHRHTGCKLAYTPKSNFEFWEQKFRKNVANDLK
          ruler 1.......10........20........30........40........50........60........70........80........90.......100
```

```
                 -----------------------------------------------------------
      E_coli_K12 DISRLQELGWRVLIVWECALRGRE-KLTDEALTERLEEWICGEGASAQIDTQGIH-LLA
   E_coli_CFT073 DINRLQALGWRVLIVWECALRGRE-KLTDEALTERLEEWICGEGASAQIDTQGIH-LLA
S_enterica_CT18 DIQRLQALGWRVLIVWECALRGRA-KLSDAALAERLEEWICGGGASAQIDTQGIH-LLA
 S_enterica_Ty2 DIQRLQALGWRVLIVWECALRGRA-KLSDAALAERLEEWICGGGASAQIDTQGIH-LLA
       S_boydii DISRLQELGWRVLIVWECALRGRE-KLTDEALTERLEEWICGEGASAQIDTQGIH-LLA
  S_dysenteriae DISRLQELGWRVLIVWECALRGRE-KLTDEALSERLEEWICGEGASAQIDTQGIH-LLA
  P_luminescens VMSSLSDNSWKILVIWECTLRGRL-KLTDNELSERIEEWLCASETHAEIDTVGIHKILL
  L_pneumophila KLKQLKMLGWKVIIVWECEIKDKNYHWIDNEIKNGLDKNKY------------------
          ruler .......110.......120.......130.......140.......150.........
```

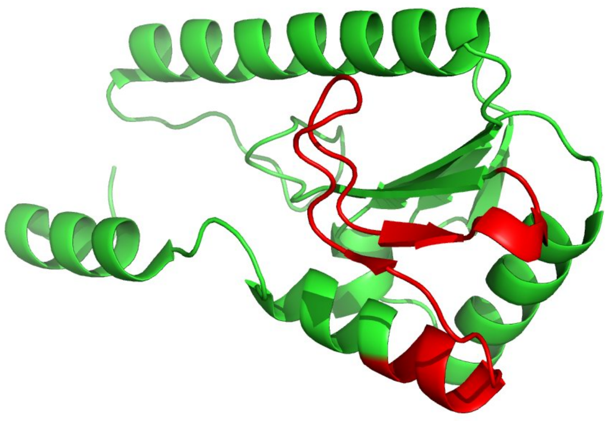

Figure S1.17: Sequence alignment and tertiary structure for gene *alkA*. The mutation cluster is highlighted in red.

E. coli K12 entry: b2068; PDB ID: 1mpg; chain ID: A

```
                ----------------------------------------------XXXXXXXXXXXXXXXXX-------------------------------------
     E_coli_K12 MYTLNWQPPYDWSWMLGFLAARAVSSVETVADSYYARSLAVGEYRGVVTAIPDIARHTLHINLSAGLEPVAAECLAKMSRLFDLQCNPQIVNGALGRLGA
  E_coli_CFT073 MYTLNWQPPYDWSWMLGFLAARAVSGVETVADSYYARSLAVGEYRGVVTAIPDIARHTLHINLSAGLEPVAAECLAKMSRLFDLQCNPQIVNGALGKLGA
S_enterica_CT18 MFTLSWQPPYDWSWMLGFLAARAVDGVETVGEGFYARSLVVGEHRGLVSVRPHLPTHTVQVSVSAGLLPVAPACLAKVSRLFDLDCQPEQVAAVLGPLGE
 S_enterica_Ty2 MFTLSWQPPYDWSWMLGFLAARAVDGVETVGEGFYARSLVVGEHRGLVSVRPHLPTHTVQVSVSAGLLPVAPACLAKVSRLFDLDCQPEQVAAVLGPLGE
       S_boydii MYTLNWQPPYDWSWMLGFLAARAVSGVETVADDYYARSLAVGEYRGVVTAIPDIARHTLHINLSAGLEPVAAECLAKMSRLFDLQCNPQIVNGALGKLGA
  S_dysenteriae MYTLNWQPPYDWSWMLGFLAARAVNGVETVADDYYARSLAVGEYRGVVTAIPDIARHTLHINLSAGLEPVAAECLAKMSRLFDLQCNPQIVNGALGKLGV
          ruler 1.......10........20........30........40........50........60........70........80........90.......100

                ----------------------------------------------------------------------------------------------------
     E_coli_K12 ARPGLRLPGCVDAFEQGVRAILGQLVSVAMAAKLTARVAQLYGERLDDFPEYICFPTPQRLAAADPQALKALGMPLKRAEALIHLANAALEGTLPMTIPG
  E_coli_CFT073 ARPGLRLPGCVDAFEQGVRAILGQLVSVAMAAKLTSRVAQLYGERLDDFPDYVCFPTPQRLAVADLQALKALGMPLKRAEALIHLANAALEGTLPMTIPG
S_enterica_CT18 DRPGLRLPGSVDTFEQGVRAILGQLVSVAMAARLTAKVARRYGEALPDAPDYVCFPGPETLALADPLALKALGMPLRRAEALIHLAQATLAGKLALSAPP
 S_enterica_Ty2 DRPGLRLPGSVDTFEQGVRAILGQLVSVAMAARLTAKVARRYGEALPDAPDYVCFPGPETLALADPLALKALGMPLRRAEALIHLAQATLAGKLALSAPP
       S_boydii ARPGLRLPGCIDAFEQGVRAILGQLVSVAMAAKLTAKVVQLYGERLDDFPEYICFPTPQRLAAADPQALKALGMPLKRAEALIHLANAALEGSLPMTIPG
  S_dysenteriae ARPGLRLPGSVDAFEQGVRAILGQLVSVAMAAKLTAKVAQLYGERLDDFPEYICFPTPQRLAAADPQALKALGMPLKRAEALIHLANAALEGTLPMTIPG
          ruler .......110.......120.......130.......140.......150.......160.......170.......180.......190.......200

                -----------------------------------------------------------------------------------------
     E_coli_K12 DVEQAMKTLQTFPGIGRWTANYFALRGWQAKDVFLPDDYLIKQRFPGMTPAQIRRYAERWKPWRSYALLHIWYTEGWQPDEA-------
  E_coli_CFT073 DVEQAMKTLQTFPGIGRWTANYFALRGWQAKDVFLPDDYLIKQRFPGMTPAQIRRYAERWKPWRSYALLHIWYTEGWQPDEA-------
S_enterica_CT18 DIEQSVKNLQTFPGIGRWTANHFALRGWQAKDIFLPDDYLIKQRFAGMTAAQIRRYAERWKPWRSYALLHIWYTHGWQPSMDSEIAGIQ
 S_enterica_Ty2 DIEQSVKNLQTFPGIGRWTANYFALRGWQAKDIFLPDDYLIKQRFAGMTAAQIRRYAERWKPWRSYALLHIWYTHGWQPSMDSEIAGIQ
       S_boydii DVEQAMKTLQTFPGIGRWTANYFALRGWQAKDVFLPDDYLIKQRFPGMTPAQIRRYAERWKPWRSYALLHIWYTEGWQPDGTDEL----
  S_dysenteriae DVEQAMKTLQTFPGIGRWTANYFALRGWQAKDVFLPDDYLIKQRFPGMTPAQIRRYAERWKPWRSYALLHIWYTEGWQPDEA-------
          ruler .......210.......220.......230.......240.......250.......260.......270.......280.........
```

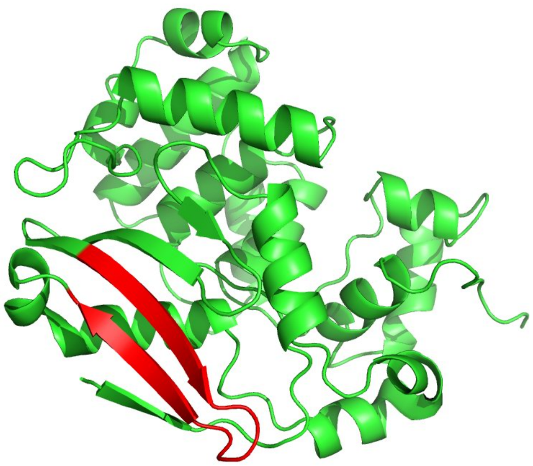

Figure S1.18: Sequence alignment and tertiary structure for gene *purN*. The mutation cluster is highlighted in red.

E. coli K12 entry: b2500; PDB ID: 1jkx; chain ID: A

```
                  ------------------------------------------------------XXXXXXXXXXXXXXXXXXXXXXXXXX--------------------
       E_coli_K12 --MNIVVLISGNGSNLQAIIDACKTNKI-KGTVRAVFSNKADAFGLERARQAGIATHTLIASAFDSREAYDRELIHEIDMYAPDVVVLAGFMRILSPAFV
     E_coli_CFT073 --MNIVVLISGNGSNLQAIIDACKTNKI-KGTVRAVFSNKADAFGLERARQAGIATHTLITSAFDSREAYDRELIHEIDMYAPDVVVLAGFMRILSPAFV
  S_enterica_CT18 --MNIVVLISGNGSNLQAIIDACEAKKI-KGTLRAVFSNKADAFGLERAREAGIPAQALTADRFDSRDAFDRELIRKIDAYAPDVVVLAGFMRILSPMFV
   S_enterica_Ty2 --MNIVVLISGNGSNLQAIIDACEAKKI-KGTLRAVFSNKADAFGLERAREAGIPAQALTADRFDSRDAFDRELIRKIDAYAPDVVVLAGFMRILSPMFV
         S_boydii --MNIVVLISGNGSNLQAIIDACKTNKI-KGTVRAVFSNKADAFGLERARQAGIATHTLIASAFDSREAYDRELIHEIDMYAPDVVVLAGFMRILSPAFV
    S_dysenteriae --MNIVVLISGNGSNLQAIIDACKTNKI-KGTVRAVFSNKADAFGLERARQAGIATHTLITSAFDSREAYDRELIHEIDMYAPDVVVLAGFMRILSPAFV
    P_luminescens -MKNIVVLISGSGSNLQAVIDACQQNRI-NGQVCAVLSNTANAYGLLRAKQADIPTHVISPKNYADRQTYDEALKHTIDQYQPDLLVLAGYMRILTPDFV
         Y_pestis -MKKIVVLISGQGSNLQALIDAQQQGRI-SGKISAVFSNNPAAYGLERAESAGIPHHALDAKAFTDRVSFDLALAQAIDQYQPDLLVLAGYMRILSPEFV
   A_ferrooxidans LTKRLVILVSGRGSNLQSILAACRSGQIPDTQVVAVISNRPAAGALELAVLAGIPALTVDHRDYGARVDFDAALQRRIDDYAPDVVALAGFMRQLTPAFV
     L_pneumophila -MIRLGILGSTRGTNMLALVDAINEGIL-KAKIELVISNKSDAIILERAKSLGLNAQFVNPEGL-NRIDFDKKVSDILINHQIDLIVLIGYMRILSADFV
            ruler 1.......10........20........30........40........50........60........70........80........90.......100

                  ----------------------------------------------------------------------------------------------------
       E_coli_K12 SHYAGRLLNIHPSLLPKYPG---LHTHRQALENGDEEHGTSVHFVTDELDGGPVILQAKVPVFAGDSEDDITARVQTQEHAIYPLVISWFADGRLKMHEN
     E_coli_CFT073 SHYAGRLLNIHPSLLPKYPG---LHTHRQALENGDEEHGTSVHFVTDELDGGPVILQAKVPVFAGDTEDDITARVQTQEHAIYPLVISWFADGRLKMHEN
  S_enterica_CT18 AHYYGRLLNIHPSLLPKYPG---LHTHRQALENGDEEHGTSVHFVTDELDGGPVILQAKVPVFANDSEDDITARVQTQEHAIYPLVIGWFAQGRLKMRDN
   S_enterica_Ty2 AHYYGRLLNIHPSLLPKYPG---LHTHRQALENGDEEHGTSVHFVTDELDGGPVILQAKVPVFANDSEDDITARVQTQEHAIYPLVIGWFAQGRLKMRDN
         S_boydii SHYAGRLLNIHPSLLPKYPG---LHTHRQALENGDEEHGTSVHFVTDELDGGPVILQAKVPVFAGDTEDDVTARVQTQEHAIYPLVISWFADGRLKIHEN
    S_dysenteriae SHYAGRLLNIHPSLLPKYPG---LHTHRQALENGDEEHGTSVHFVTDELDGGPVILQAKVPVFAGDTEDDVTARVQTQEHAIYPLVISWFADGRLKMHEN
    P_luminescens QHYLGRLLNIHPSLLPKYPG---LHTHRKAITNGDTEHGTSVHFVTEELDGGPVILQAKVPIFAGDQENEVVKRVQTQEHNIYPLVINWFIEGRLSMVNG
         Y_pestis KHYAGRMLNIHPSLLPKYPG---LHTHRQALENGDQEHGTSVHFVTEELDGGPVILQAKVPIFSDDSEEDVAERVQTQEHSIYPLVVSWFTDGRLAMRDN
   A_ferrooxidans QHYEGRMLNVHPSLLPAFPG---LHTHARALEQGVLWHGASVHFVTSALDAGPAIIQAAVAVLPEDDEQSLAARVLDAEHRIYPQALAWLLAGRVAYAAG
     L_pneumophila NKWNNQVINVHPSLLPAFAGKMDMDVHQAVLDSGLKETGCTIHFVTEEVDAGPVILQKKCPVLEGDTAQTLKARVQQLEGMALVAAINLIASKGL-----
            ruler .......110.......120.......130.......140.......150.......160.......170.......180.......190.......200

                  --------------------------
       E_coli_K12 AA-WLDGQRLPPQGYAADE-------
     E_coli_CFT073 AA-WLDGQRLPPQGYAADE-------
  S_enterica_CT18 AA-WLDGRRLPPQGYASDE-------
   S_enterica_Ty2 AA-WLDGRRLPPQGYASDE-------
         S_boydii AA-WLDGQRLPPQGYAADE-------
    S_dysenteriae AA-WLDGQRLPPQGYAADE-------
    P_luminescens KA-YLDGKILPPEGYAAE--------
         Y_pestis AA-WLDGVRLPLQGYAAE--------
   A_ferrooxidans RAQWRD----PPETAMREAIAPSLEM
     L_pneumophila --------------------------
            ruler .......210.......220......
```

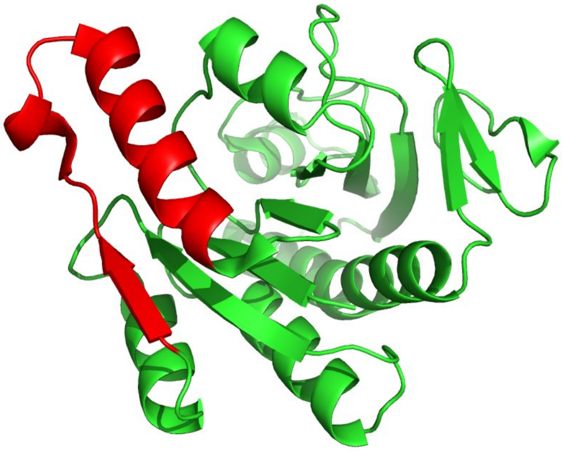

Figure S1.19: Sequence alignment and tertiary structure for gene *glyA*. The mutation cluster is highlighted in red.

E. coli K12 entry: b2551; PDB ID: 1eqb; chain ID: B

```
                ----------------------------------------------------------------------------------------------------
      E_coli_K12 --MLKREMNIADYDAELWQAMEQEKVRQEEHIELIASENYTSPRVMQAQGSQLTNKYAEGYPGKRYYGGCEYVDIVEQLAIDRAKELFGADYANVQPHSG
   E_coli_CFT073 MRMLKREMNIADYDAELWQAMEQEKVRQEEHIELIASENYTSPRVMQAQGSQLTNKYAEGYPGKRYYGGCEYVDIVEQLAIDRAKELFGADYANVQPHSG
S_enterica_CT18 --MLKREMNIADYDAELWQAMEQEKVRQEEHIELIASENYTSPRVMQAQGSQLTNKYAEGYPGKRYYGGCEYVDVVEQLAIDRAKELFGADYANVQPHSG
 S_enterica_Ty2 --MLKREMNIADYDAELWQAMEQEKVRQEEHIELIASENYTSPRVMQAQGSQLTNKYAEGYPGKRYYGGCEYVDVVEQLAIDRAKELFGADYANVQPHSG
        S_boydii --MLKREMNIADYDAELWQAMEQEKVRQEEHIELIASENYTSPRVMQAQGSQLTNKYAEGYPGKRYYGGCEYVDIVEQLAIDRAKELFGADYANVQPHSG
   S_dysenteriae --MLKREMNIADYDAELWQAMEQEKVRQEEHIELIASENYTSPRVMQAQGSQLTNKYAEGYPGKRYYGGCEYVDIVEQLAIDRAKELFGADYANVQPHSG
   P_luminescens --MLKREMNIASYDPELWQAMEQEVVRQEEHIELIASENYTSPRVMQAQGSQLTNKYAEGYPGKRYYGGCEYVDVVEQLAIDRAKALFGADYANVQPHSG
        Y_pestis --MLKREMNIADYDADLWRAMEQEVVRQEEHIELIASENYTSPRVMQAQGSQLTNKYAEGYPGKRYYGGCEYVDVVEQLAIDRAKALFGADYANVQPHSG
    B_aphidicola --MFIKNKTISNYDADVYRMMKQEYQRQENHIELIASENYVSSCVMEAQGSQLTNKYAEGYPGKRYYGGCDYVDAIEKIAIKRAKKLFNANYANVQPHSG
  A_ferrooxidans --MFSKTLTIADFDPVLWDAMRKEARRQEDHVELIASENYASPMVMAAQGSVLTNKYAEGYPGKRYYGGCEYVDIAEQLAMDRALELFGAEHANVQAHSG
   L_pneumophila --MFDESYTIKNFDDVLFKAISDEKRRQEEHIELIASENYVSPRVLEAQGSVLTNKYAEGYPGKRYYGGCEFVDVAEELAISRAKLLFGAHYVNVQPHSG
           ruler 1.......10........20........30........40........50........60........70........80........90.......100

                ---------------------------------------------------XXXXXXXXXXXXX------------------------------------
      E_coli_K12 SQANFAVYTALLEPGDTVLGMNLAHGGHLTHGSPVNFSGKLYNIVPYGID-ATGHIDYADLEKQAKEHKPKMIIGGFSAYSGVVDWAKMREIADSIGAYL
   E_coli_CFT073 SQANFAVYTALLEPGDTVLGMNLAHGGHLTHGSPVNFSGKLYNIVPYGID-ATGHIDYADLEKQAKEHKPKMIIGGFSAYSGVVDWAKMREIADSIGAYL
S_enterica_CT18 SQANFAVYTALLQPGDTVLGMNLAQGGHLTHGSPVNFSGKLYNIVPYGID-ESGKIDYDEMAKLAKEHKPKMIIGGFSAYSGVVDWAKMREIADSIGAYL
 S_enterica_Ty2 SQANFAVYTALLQPGDTVLGMNLAQGGHLTHGSPVNFSGKLYNIVPYGID-ESGKIDYDEMAKLAKEHKPKMIIGGFSAYSGVVDWAKMREIADSIGAYL
        S_boydii SQANFAVYTALLEPGDTVLGMNLAHGGHLTHGSPVNFSGKLYNIVPYGID-ATGHIDYADLEKQAKEHKPKMIIGGFSAYSGVVDWAKMREIADSIGAYL
   S_dysenteriae SQANFTVYTALLEPGDTVLGMNLAHGGHLTHGSPVNFSGKLYNIVPYGID-ATGHIDYADLEKQAKEHKPKMIIGGFSAYSGVVDWAKMREIADSIGAYL
   P_luminescens SQANAAVYMALLQPGDTILGMNLAHGGHLTHGSPVNFSGKLYNVVPYGID-ESGKIDYDDIAAQAEKHQPKMIIGGFSAYSGVVDWAKMREIADSIGAYL
        Y_pestis SQANVAVYSALLKPGDTVLGMNLAHGGHLTHGSPVNFSGKLYNIVPYGID-ESGQIDYEDLARQAEIHKPKMIIGGFSAYSGIVDWAKMREIADSIDAWF
    B_aphidicola SQANYAVYSALLKPNDIVLGMSLSHGGHLTHGSSVNFSGKLYKFISYGLD-ISGDIDYPQIRKLAHRYKPKMIVGGFSAYSGICNWKLLREIADEINSYL
  A_ferrooxidans SQANQAVYLSVLQPGDKIMGMSLAHGGHLTHGAKVNVSGKLFQVAAYGVRAEDGRIDYDAMAEQAERERPKMIVAGASAYSRVIDFARIGEIARSIGAYL
   L_pneumophila SQANAAVMMALLSPGDTFMGMALPHGGHLTHGSKVNFSGKLYHSVEYGVDNNTGLIDYDALEKLALQHKPKLIIAGFSAYSRILDWARFREIADKVGAYL
           ruler .......110.......120.......130.......140.......150.......160.......170.......180.......190.......200

                ----------------------------------------------------------------------------------------------------
      E_coli_K12 FVDMAHVAGLVAAGVYPNPVPHAHVVTTTTHKTLAGPRGGLILAKGGSEELYKKLNSAVFPGGQGGPLMHVIAGKAVALKEAMEPEFKTYQQQVAKNAKA
   E_coli_CFT073 FVDMAHVAGLVAAGVYPNPVPHAHVVTTTTHKTLAGPRGGLILAKGGSEELYKKLNSAVFPGGQGGPLMHVIAGKAVALKEAMEPEFKTYQQQVAKNAKA
S_enterica_CT18 FVDMAHVAGLIAAGVYPNPVPHAHVVTTTTHKTLAGPRGGLILAKGGDEELYKKLNSAVFPSAQGGPLMHVIAGKAVALKEAMEPEFKVYQQQVAKNAKA
 S_enterica_Ty2 FVDMAHVAGLIAAGVYPNPVPHAHVVTTTTHKTLAGPRGGLILAKGGDEELYKKLNSAVFPSAQGGPLMHVIAGKAVALKEAMEPEFKVYQQQVAKNAKA
        S_boydii FVDMAHVAGLVAAGVYPNPVPHAHVVTTTTHKTLAGPRGGLILAKGGSEELYKKLNSAVFPGGQGGPLMHVIAGKAVALKEAMEPEFKTYQQQVAKNAKA
   S_dysenteriae FVDMAHVAGLVAAGVYPNPVPHAHVVTTTTHKTLAGPRGGLILAKGGSEELYKKLNSAVFPGGQGGPLMHVIAGKAVALKEAMEPEFKTYQQQVAKNAKA
   P_luminescens FVDMAHVAGLIAAGVYPNPVPHAHIVTTTTHKTLAGPRGGLILAKGGDEELYKKLNSSVFPGCQGGPLMHVIAGKAVALKEAMEPEFKAYQHQVADNAKA
        Y_pestis FVDMAHVAGLVAAGVYPNPVPHAHIVTTTTHKTLAGPRGGLILAKGGDEDLYKKLNSSVFPGNQGGPLMHVIAGKAVALKEAMEPEFKIYQQQVAKNAKA
    B_aphidicola FVDMAHISGLVAAGLYPNPLKYAHVVTSTTHKTLSGPRGGLILAKGDNVSLFKKLNSSVFPGCQGGPLMHVIAAKAIAFKEAMEPEFKDYQYQVIKNAQE
  A_ferrooxidans LVDMAHIAGLVATGLHPSPVPHADFVTTTTHKTLRGPRGGLILCR---EQYAKKVNSLIFPGLQGGPLMHVIAAKAVAFREALQPEFKSYQQQVIHNAQT
   L_pneumophila MADIAHVAGLVAVGLYPSPVPYADVVTTTTHKTLRGPRGGLILCK-ENEEIEKKLNSAVFPGMQGGPLMHVIAAKAVAFAEALLPEFKTYQQQVLANART
           ruler .......210.......220.......230.......240.......250.......260.......270.......280.......290.......300
```

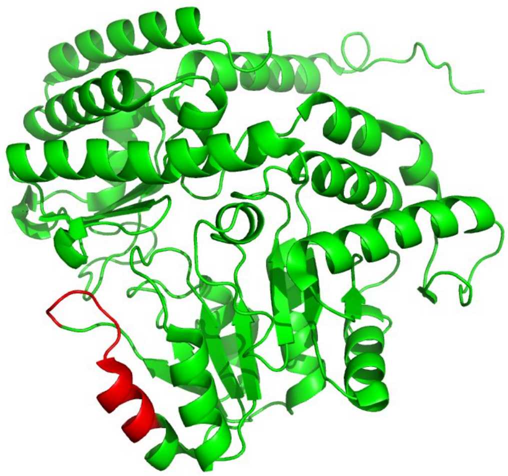

Figure S1.20: Sequence alignment and tertiary structure for gene *truD*. The mutation cluster is highlighted in red.

E. coli K12 entry: b2745; PDB ID: 1si7; chain ID: A

```
                  ----------------------------------------------------------------------------------------------------
      E_coli_K12 MIEFDNLTYLHGKPQGTGLLKAN----PEDFVVVEDLGFEPDGEGEHILVRILKNGCNTRFVADALAKFLKIHAREVSFAGQKDKHAVTEQWLCARVPGK
   E_coli_CFT073 MIEFDNLTYLHGKPQGTGLLKAN----PEDFVVVEDLGFEPDGEGEHILVRILKNGCNTRFVADALAKFLKIHAREVSFAGQKDKHAVTEQWLCARVPGK
 S_enterica_CT18 MTEFDNLTWLHGKPQGSGLLKAN----PEDFVVVEDLGFTPDGEGEHILLRILKNGCNTRFVADVLAKFLKIHAREVSFAGQKDKHAVTEQWLCARVPGK
  S_enterica_Ty2 MTEFDNLTWLHGKPQGSGLLKAN----PEDFVVVEDLGFTPDGEGEHILLRILKNGCNTRFVADVLAKFLKIHAREVSFAGQKDKHAVTEQWLCARVPGK
        S_boydii MIEFDNLTYLHGKPQGTGLLKAN----PEDFVVVEDLGFEPDGEGEHILVRILKNGCNTRFVADALAKFLKIHAREVSFAGQKDKHAVTEQWLCARVPGK
   S_dysenteriae MIEFDNLTYLHGKPQGTGLLKAN----PEDFVVVEDLGFEPDGEGEHILVRILKNGCNTRFVADALAKFLKIHAREVSFAGQKDKHAVTEQWLCARVPGK
   P_luminescens -MVLAELNWLYGQPEATGILKAS----PEDFIVCEDLGFSPDGEGEHLMVHIRKTGCNTQFVADSLARFAGIPSRSVSYAGLKDRHAVTEQWFCLHLPGK
        Y_pestis -MDMENLTWLHGKPTASGILKAN----PEDFVVVEDLGFEPDGEGEHLLVRIRKNGCNTQFVADYLARFAKLHPRLVSYAGLKDRHAVTEQWFCLHLPGK
  A_ferrooxidans --MSESLPRIY--PAESGLLGAEMRQSPEDFQVTELLPFAPSGLGAHHWLVVEKRGLNTQDVANRLARCAGVAVRDVGFAGLKDRQAVTRQSFTVPATAG
    L_pneumophila -MYSLNWPRAYGIPNSTATFKLC----PEDFQVNELFEGQFSGEGEHIVLKIEKKGLTTEQVIKSLARLINKPIKLISYAGLKDKQALTTQWLSIHAPGE
           ruler 1.......10........20........30........40........50........60........70........80........90.......100

                  -----------------------------------------XXXXXXX-XXXXXXXXXX-----------------------------------------
      E_coli_K12 EMPDLSAFQLEGCQVLEYARHKRKLRLGALKGNAFTLVLREVSNRDDV-EQRLIDICVKGVPNYFGAQRFGIGGSNLQGAQRWAQTNTPVRDRNKRSFWL
   E_coli_CFT073 EMPDLSAFQLEGCQVLEYARHKRKLRLGALKGNAFTLVLREVSNRDDV-EQRLIDICVKGVPNYFGAQRFGIGGSNLQGALRWAQTNTPVRDRNKRSFWL
 S_enterica_CT18 EMPDFSAFQLEGCKVLEYARHKRKLRLGALKGNAFTLVLREISDRRDV-ETRLQAIRDGGVPNYFGAQRFGIGGSNLQGALRWAQSNAPVRDRNKRSFWL
  S_enterica_Ty2 EMPDFSAFQLEGCKVLEYARHKRKLRLGALKGNAFTLVLREISDRRDV-ETRLQAIRDGGVPNYFGAQRFGIGGSNLQGALRWAQSNAPVRDRNKRSFWL
        S_boydii EMPDLSAFQLEGCQVLEYARHKRKLRLGALKGNAFTLVLREVSNRDDV-EQRLIDICVKGVPNYFGAQRFGIGGSNLQGALRWAQTNTPVRDRNKRSFWL
   S_dysenteriae EMPDLSAFQLEGCQVLEYARHKRKLRLGALKGNAFTLVLREVSNRDDV-EQRLIDICVKGVPNYFGAQRFGIGGSNLQGALRWAQTNTPVRDRNKRSFWL
   P_luminescens QEPDFASFQLEGCEILTTARQKRKLRIGALKGNSFTLILREISHHQLV-ENRLRLIQQGGVPNYFGEQRFGRDGQNLIQAQRWANNEIRVKERSKRSFYL
        Y_pestis EAPDLATFELEGCEVLEAVRHKRKLRIGSLKGNAFTLVLRHITDRQDV-EQRLQQIAAQGVPNYFGSQRFGRGGNNLVQARLWANNEIRVKERSKRSFYL
  A_ferrooxidans SNPDWGSLEDDSLHLLQVGRHDRKLRRGVLRGNHFQLTLRATTGAHSLWDARLQILRKRGFPNYFGAQRFGH--RNLQEAARLLSGQPMRVDRHRRGLLW
    L_pneumophila VIEGIETLEAPGWKILECTRHNKKLRPGFLSGNHFTITLRNVSDESDL-IHRIEQIKFKGVPNYFGEQRFGRDGGNLIKAEEILVQGRKVKDRFLKGMYF
           ruler .......110.......120.......130.......140.......150.......160.......170.......180.......190.......200

                  ----------------------------------------------------------------------------------------------------
      E_coli_K12 SAARSALFNQIVAERLKKADVNQVVDGDALQLAGRGSWFVATTEELAELQRRVNDKELMITAALPGSGEWGTQREALAFEQAAVAAETE----------L
   E_coli_CFT073 SAARSALFNQIVAERLKKADVNQVVDGDALQLAGRGSWFVATTEELAELQRRVNDKELMITAALPGSGEWGTQREALAFEQAAVAEETE----------L
 S_enterica_CT18 SAARSALFNQIVHQRLKKPDFNQVVDGDALQLAGRGSWFVATSEELPELQRRVDEKELMITASLPGSGEWGTQRAALAFEQDAIAQETV----------L
  S_enterica_Ty2 SAARSALFNQIVHQRLKKPDFNQVVDGDALQLAGRGSWFVATSEELPELQRRVDEKELMITASLPGSGEWGTQRAALAFEQDAIAQETV----------L
        S_boydii SAARSALFNQIVAERLKKADVNQVVDGDALQLAGRGSWFVATTEELAELQHRVNDKELMITAALPGSGEWGTQRKALAFEQAAVAAETE----------L
   S_dysenteriae SAARSALFNQIVAERLKKADVNQVVDGDALQLAGRGSWFVATTEELAELQRRVNDKELMITAALPGSGEWGTQREALAFEQAAVAAETE----------L
   P_luminescens SASRSAMFNAVASARIALKQQQQVIKGDALQLTGRGSWFVADETELPLLQQRVIDGELQITAPLPGDGELGTQHQAADFERQYLQPYES----------L
        Y_pestis SASRSAMFNLISSYRLAQQLSTTVLEGDALQLSGRGSWFVAQADELAALQQRVTAGELNITAPLPGDSELGTHGEALAFEQACLAEQTE----------L
  A_ferrooxidans SAARSALFNLVLAERVRQANWTEILPGEVVQLAGSHSIFLAEDDDLADLQARATDWDLHPTGPLPGRGGLAPTLVAAALETSALGAGPVIAGTTDDGLHW
    L_pneumophila SAARSWLYNLILSRRVKESSWNLPLLGDVIQLVGSNSIFVNDKSLDEQLLQRIGEKDVSPASPLPGRSKNLVKGTALQIINEVYAEWSA----------W
           ruler .......210.......220.......230.......240.......250.......260.......270.......280.......290.......300
```

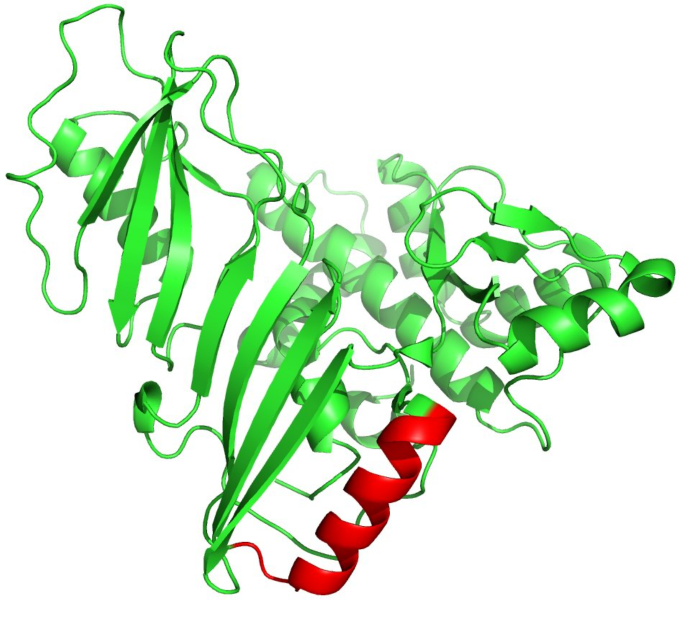

Figure S1.21: Sequence alignment and tertiary structure for gene *recC*. The mutation cluster is highlighted in red.

E. coli K12 entry: b2822; PDB ID: 1w36; chain ID: C

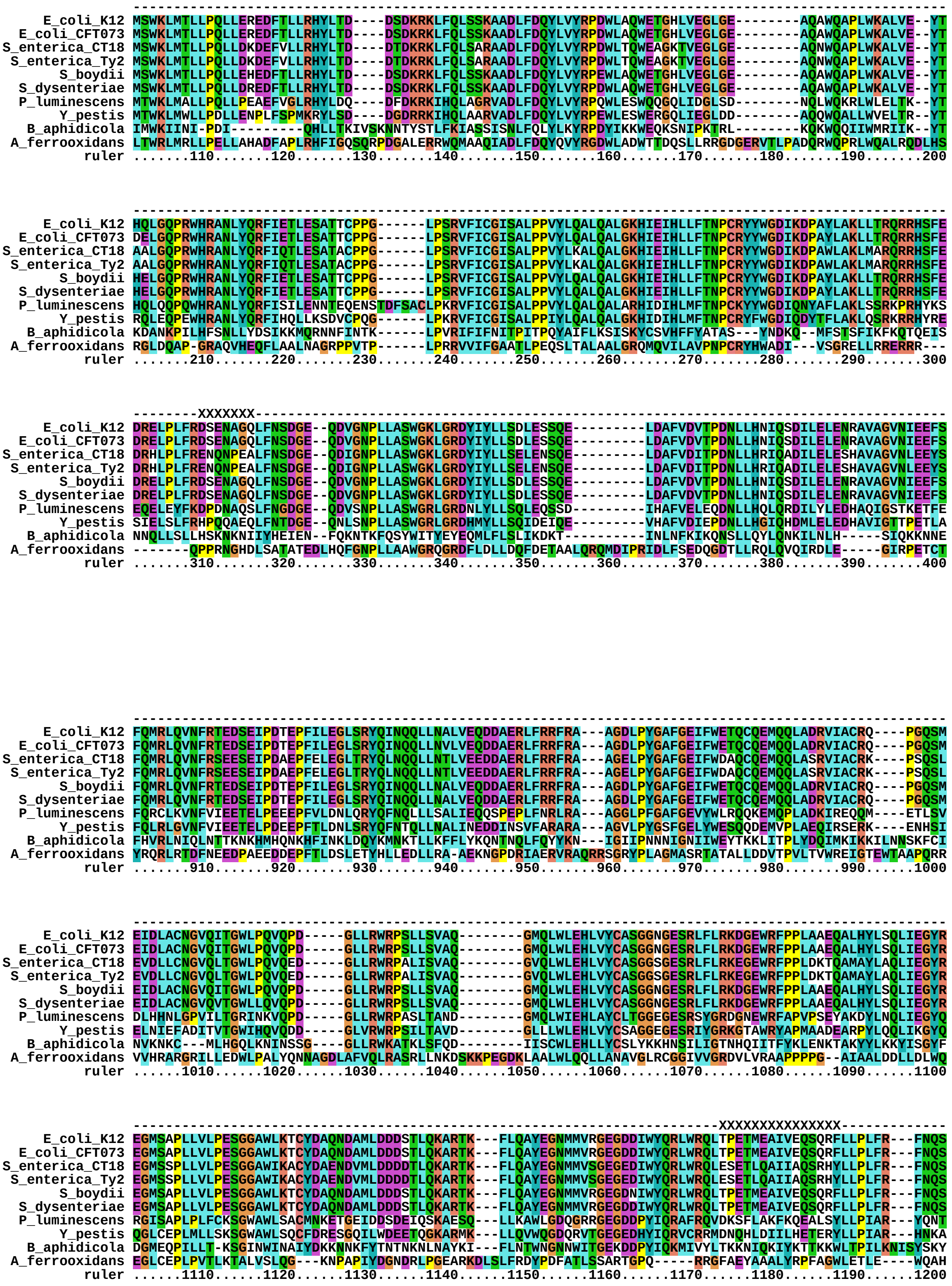

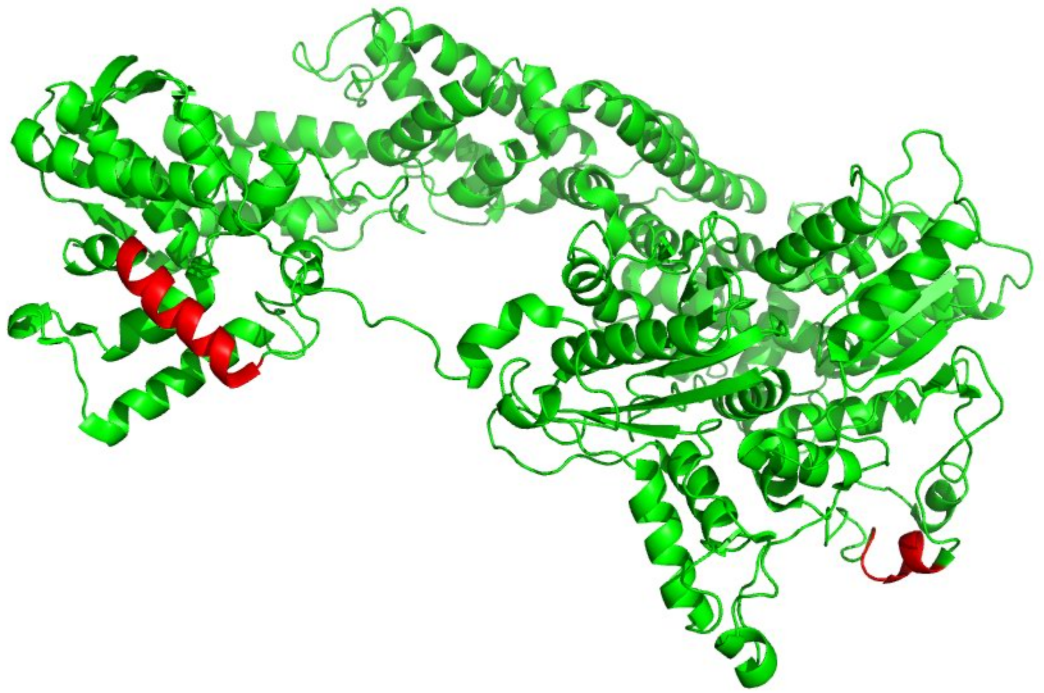

Figure S1.22: Sequence alignment and tertiary structure for gene *lysA*. The mutation cluster is highlighted in red.

E. coli K12 entry: b2838; PDB ID: 1knw; chain ID: A

```
                ----------XXXXXXXX--------------------------------------------------------------------------------
      E_coli_K12 ----------MPHSLFSTDTDLTAEN--LLRLPAEFGCPVWVYDAQIIRRQI----AALKQFDVVRFAQKACSNIHILRLMREQGVKVDSVSLGEIERAL
   E_coli_CFT073 ----------MPHSLFSTDTDLTAEN--LLRLPAEFGCPVWVYDAQIIRRQI----AALKQFDVVRFAQKACSNIHILRLMREQGVKVDSVSLGEIERAL
 S_enterica_CT18 ----------MSLPHYHAETDLNAEN--LLRLPAEFGCPVWVYDAHIIRRQI----AALQQFDVVRFAQKACSNIHILRLMRAQGVKVDSVSLGEIERAL
  S_enterica_Ty2 ----------MSLPHYHAETDLNAEN--LLRLPAEFGCPVWVYDAHIIRRQI----AALQQFDVVRFAQKACSNIHILRLMRAQGVKVDSVSLGEIERAL
        S_boydii ----------MPHSLFSTDTDLTAEN--LLRLPAEFGCPVWVYDAQIIRRQI----AALKQFDVVRFAQKACSNIHILRLMREQGVKVDSVSLGEIERAL
   S_dysenteriae ----------MPHSLFSTDTALTAEN--LLRLPAEFGCPVWVYDAQIIRRQI----AALKQFDVVRFAQKACSNIHILRLMREQGVKVDSVSLGEIERAL
   P_luminescens VRETLMSTTFTPNFTRNNCPNLIPES--LGRLPETYGTPLWVYDSETIIQKI----NLLCQFDVVRFAQKACSNTHILRLMREYGVKVDAVSLGEIERAL
        Y_pestis ----------MPRALNDRSSALTAQN--LIALPERFGCPVWAYDGDIIAERI----NQLRHFDVIRFAQKACSNIHILRLMREQGVKVDSVSLGEIERAL
    B_aphidicola -----------VSGLLKKKQRFSCND--ILKIVSKYGTPLWVYDSNIIVKKI----KELSQFDVIRFAQKSCSNIHILNLFYKYNVKIDAVSLGEIERAL
  A_ferrooxidans -----------MNTFHYRDHALYGEDLPLQEIAAHYGTPCYVYSEAALRERYHSFSAALGDNTRVCYAVKANSNLGILRIFAEMGAGFDIVSGGELERVL
           ruler 1.......10........20........30........40........50........60........70........80........90.......100

                ----------------------------------------------------------------------------------------------------
      E_coli_K12 AAGYNPQTHPDDIVFTADVIDQATL-ERVSELQIPVNAGSVDMLDQLGQVSPGHRVWLRVNPGFGHGHSQKTNTGGENSKHGI-WYTDLPAALDVIQRHH
   E_coli_CFT073 AAGYNPQTHPDDIVFTADVIDQATL-ERVSELQIPVNAGSVDMLDQLGQVSPGHRVWLRVNPGFGHGHSQKTNTGGENSKHGI-WYTDLPAALDVIQRHH
 S_enterica_CT18 AAGYDPQTCPDDIVFTADVIDAATL-ARVSELHIPVNAGSVDMLTQLGQVSPGHRVWLRVNPGFGHGHSQKTNTGGENSKHGI-WHSDLPAALAVMQKYR
  S_enterica_Ty2 AAGYDPQTCPDDIVFTADVIDAATL-ARVSELHIPVNAGSVDMLTQLGQVSPGHRVWLRVNPGFGHGHSQKTNTGGENSKHGI-WHSDLPAALAVMQKYR
        S_boydii AAGYNPQTHPDDIVFTADVIDQATL-ERVSELQIPVNAGSVDMLDQLGQVSPGHRVWLRVNPGFGHGHSQKTNTGGENSKHGI-WYTDLPAALDVIQRHH
   S_dysenteriae AAGYNPQTHPDDIVFTADVIDQATL-ERVSELQIPVNAGSVDMLDQLGQVSPGHRVWLRVNPGFGHGHSQKTNTGGENSKHGI-WYTDLPAALDVIQRHH
   P_luminescens RAGFCPGQEVSDIIFTADLIDVSTL-QRVTELDIPVNAGSIDMLEQIGQYKKGHPVWLRINPGFGHGHSQKTNTGGENSKHGI-WYQNLPQAIEKIRHYG
        Y_pestis HAGYQPGQEPAEIVFTADLLDQATL-LRVTELNIPVNAGSIDMLDQLGQQAPGHPVWLRVNPGFGHGHSQKTNTGGENSKHGI-WHEELPRALKKIEHYG
    B_aphidicola ISKYRF-TMDQDVVFTSDIIERETL-NKVVQYKIPINIGSIDMLEQVGKISPGHNIWLRINPKFGHGHSKKTNTGGENSKHGI-WDINL--AFPYITKYN
  A_ferrooxidans RAGGDAGTIVFSGVGKSSVEIRAALGAGIFCLNVESAAELWRIADIAADMGKRAPVALRVNPDVDPGTHRYIATGLKESKFGIPMDQARHLYLQAAQHPA
           ruler .......110.......120.......130.......140.......150.......160.......170.......180.......190.......200

                ----------------------------------------------------------------------------------------------------
      E_coli_K12 LQLVGIHMHIGSGV-DYAHLEQVCG---AMVRQVIEFGQDLQAISAGGGLSVPYQQGEEAVDTEHYYGLWNAAREQIARHLGHPVKLEIEPGRFLVAQSG
   E_coli_CFT073 LQLVGIHMHIGSGV-DYAHLEQVCG---AMVRQVLEFGQDLQAISAGGGLSIPYQQGEEAVDTEHYYGLWNAAREQIARHLGHPVKLEIEPGRFLVAQAG
 S_enterica_CT18 LKLVGIHMHIGSGV-DYGHLEQVCG---AMVRQVLECGQDVEAISAGGGLSIPYHEGEEPVDTRHYYGLWNAAREQIARHLGHAVKLEIEPGRYLVAQSG
  S_enterica_Ty2 LKLVGIHMHIGSGV-DYGHLEQVCG---AMVRQVLECGQDVEAISAGGGLSIPYHEGEEPVDTRHYYGLWNAAREQIARHLGHAVKLEIEPGRYLVAQSG
        S_boydii LQLVGIHMHIGSGV-DYAHLEQVCG---AMVRQVIEFGQDLQAISAGGGLSVPYQQGEEAVDTEHYYGLWNAAREQIARHLGHPVKLEIEPGRFLVAQSG
   S_dysenteriae LQLVGIHMHIGSGV-DYAHLEQVCG---AMVRQVIEFGQDLQAISAGGGLSIPYQQGEEAVDTEHYYGLWNAARQQIARHLGHPVKLEIEPGRFLVAQSG
   P_luminescens LKLLGLHMHIGSGV-DYHHLASVCN---AMVEQVILCGEDIQAISAGGGLSTPYHQGDERINIAHYFGLWDDARKRIEQHLGHKIELEIEPGRFLVAESG
        Y_pestis LTLVGIHMHIGSGV-DYQHLEQVCD---AMVQQVITLGHDISAISAGGGLSIPYQFGDDVIDTEHYYGLWNSARERIAAHLGHPVSLEIEPGRFLVAESG
    B_aphidicola FKLIGLHMHIGSGV-DYCHLKRVCK---AMIDQVLQCKFKIHVISAGGGLTVPYHCNDEPVNVKNYFFLWNKARKIISNYLNRPIRLEIEPGRFLVAESG
  A_ferrooxidans LELKGIACHIGSQLLDLTPLGEAAGRVRALYDELAAHGIALQHLDLGGGVGIRYHDEIPPSPADYAWTLDQALQ-------GVAVRRVVELGRALAGNAG
           ruler .......210.......220.......230.......240.......250.......260.......270.......280.......290.......300
```

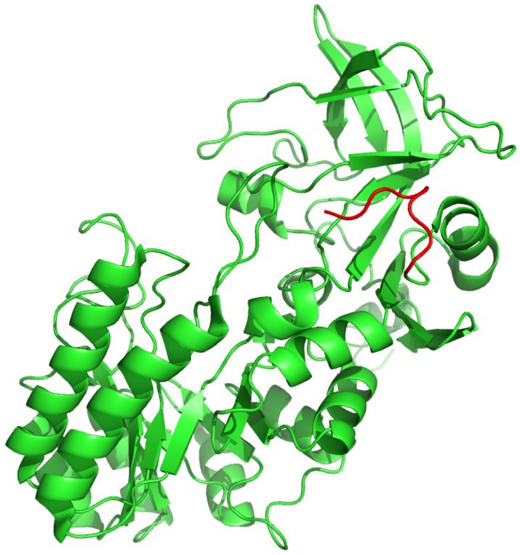

Figure S1.23: Sequence alignment and tertiary structure for gene *malP*. The mutation cluster is highlighted in red.

E. coli K12 entry: b3417; PDB ID: 1ahp; chain ID: A

```
                  ----XXXXXX------------XXXXXXXXXXXXXXXXXXXXXXXXXXX-----XXXXXXXXXXXXXXXXXXXXXX------------------------
       E_coli_K12 MSQPIFNDKQ------------FQEALSRQWQRYGLNSAAEMTPRQWWL-----AVSEALAEMLRAQPFAKPVANQRHVNYISMEFLIGRLTGNNLLNLG
   E_coli_CFT073 MSQPIFNDKQ------------FQEALSRQWQRYGLNSAAEMTPRQWWL-----AVSEALAEMLRAQPFAKPVANQRHVNYISMEFLIGRLTGNNLLNLG
S_enterica_CT18 MSQPTFNKDQ------------FQAALTRQWQRFGLLSASDMTPRQWWQ-----AVSGALAELLSAQPVAQPTKGQRHVNYISMEFLIGRLTGNNLLNLG
 S_enterica_Ty2 MSQPTFNKDQ------------FQAALTRQWQRFGLLSASDMTPRQWWQ-----AVSGALAELLSAQPVAQPTKGQRHVNYISMEFLIGRLTGNNLLNLG
        S_boydii MSQPIFNDKQ------------FQEALSRQWQRYGLNSAAEMTPRQWWL-----AVSEALAEMLRAQPFAKPVANQRHVNYISMEFLIGRLTGNNLLNLG
   S_dysenteriae MSQPIFNDKQ------------FQEALSRQWQRYGLNSAAEITPRQWWL-----AVSEALAEMLRAQPFAKPVANQRHVNYISMEFLIGRLTGNNLLNLG
   P_luminescens MQQSRLDKAQ------------FQAALTRQWQRFGLGSAQEMTQHQWWQ-----AVSAALSELVPACPVINHSEQQRHVNYISMEFLIGRLTANNLLNLG
        Y_pestis -MTSPFSYTS---PVVSVDA--LKHSIAYKLMFI-IGKDPTIATQHDWLNATLFAVRDRMVERWLRSNRAQLSQDVRQVYYLSMEFLLGRTLSNALLSMG
 A_ferrooxidans VSSQPLSKTACPLPPLGMDALSLRMDTCHYLLRT-LGAEETAEPANSVYTALAMSLRDRLVERWKNTQKKVAAESGRRTFYLSLEFLLGRAMGNALLNLG
           ruler 1.......10........20........30........40........50........60........70........80........90.......100

                  ----------------------------------------------------------------------------------------------------
       E_coli_K12 WYQDVQDSLKAYDINLTDLLEEEIDPALGNGGLGRLAACFLDSMATVGQSATGYGLNYQYGLFRQSFVDGKQVEAPDDWHRSNYPWFRHNEALDVQVGIG
   E_coli_CFT073 WYQDVQDSLKAYDINLTDLLEEEIDPALGNGGLGRLAACFLDSMATVGQSATGYGLNYQYGLFRQSFVDGKQVEAPDDWHRGNYPWFRHNEALDVQVGIG
S_enterica_CT18 WYQDVSDVLKAHDINLTDLLEEEVDPALGNGGLGRLAACFLDSMATVGQSATGYGLNYQYGLFRQSFVEGKQMEAPDDWHRGSYPWFRHNEALDVQVGIG
 S_enterica_Ty2 WYQDVSDVLKAHDINLTDLLEEEVDPALGNGGLGRLAACFLDSMATVGQSATGYGLNYQYGLFRQSFVEGKQMEAPDDWHRGSYPWFRHNEALDVQVGIG
        S_boydii WYQEVQDSLKAYDINLTDLLEEEIDPALGNGGLGRLAACFLDSMATIGQSATGYGLNYQYGLFRQSFVDGKQVEAPDDWHRSNYPWFRHNEALDVQVGIG
   S_dysenteriae WYQDVQDSLKAYDINLTDLLEEEIDPALGNGGLGRLAACFLDSMATVGQSATGYGLNYQYGLFRQSFVDGKQVEAPDDWHRSNYPWFRHNEALDVQVGIG
   P_luminescens WYEDVQAYLAEENIILSDLLEQETDPALGNGGLGRLAACFLDSMATVGQSAIGYGLNYQYGLFRQSFEQGQQIEEPDNWGRESYPWFRHNTQLDVEVNFG
        Y_pestis IYDEIEQALDEMGLSLSELLKEENDPGLGNGGLGRLAACFLDSLATLALPGRGYGIRYEYGMFSQKIVNGQQMESPDNWLEYGNAWEFPRHNTRYKVRFG
 A_ferrooxidans LEDAAREALTREHRELVDIMELEPDAGLGNGGLGRLAACFLDSCASLGLPVTGYGIRYSYGMFRQEIRGGEQVEEPDHWLRNGCPWELKRPERVRRVHFF
           ruler .......110.......120.......130.......140.......150.......160.......170.......180.......190.......200

                  ----------------------------------------------------------------------------------------------------
       E_coli_K12 GK--VTKDG----------RWEPEFTITGQAWDLPVVGYRNGVAQPLRLWQATHAHPFDLTKFNDGDFLRAEQQGINAEKLTKVLYPNDNHTAGKKLRLM
   E_coli_CFT073 GK--VTKDG----------RWEPEFTITGQAWDLPVVGYRNGVAQPLRLWQATHAHPFDLTKFNDGDFLRAEQQGINAEKLTKVLYPNDNHTAGKKLRLM
S_enterica_CT18 GK--VTKEG----------RWKPGFVITGQSWDLPVLGYRNGVAQPLRLWQATHAHPFDLTKFNDGAFLRAEQQGIDAEKLTKVLYPNDNHTAGKKLRLM
 S_enterica_Ty2 GK--VTKEG----------RWKPGFVITGQSWDLPVLGYRNGVAQPLRLWQATHAHPFDLTKFNDGAFLRAEQQGIDAEKLTKVLYPNDNHTAGKKLRLM
        S_boydii GK--VTKDG----------RWEPEFTITGQAWDLPVVGYRNGVAQPLRLWQATHAHPFDLTKFNDGDFLRAEQQGINAEKLTKVLYPNDNHTAGKKLRLM
   S_dysenteriae GK--VTKDG----------RWEPEFTITGQAWDLPVVGYRNGVAQPLRLWQATHAHPFDLTKFNDGDFLHAEQQGINAEKLTKVLYPNDNHTAGKKLRLM
   P_luminescens GKVVITDDGHE--------TWQPEFTLIGEAWDLPVIGYRNGVTQPLRLWQATHASPFDLDKFNAGQFLTAEQQGVEAAKLTKVLYPNDNHQEGKRLRLM
        Y_pestis GR--IQQEGSK-------IRWLETEEILACAYDQIIPGFDTDATNTLRLWSAQASNEINLGKFNQGDYFAAVEDKNHSENVSRVLYPDDSTYSGRELRLR
 A_ferrooxidans GR----SDGYHDAQGRFHQRWVDTHDVLAVPYDIPIPGYRNEVVNTLRLWRATATDIFDLGEFNAGAYPEAVAAKNNAEHISMVLYPNDSSENGKELRLR
           ruler .......210.......220.......230.......240.......250.......260.......270.......280.......290.......300
```

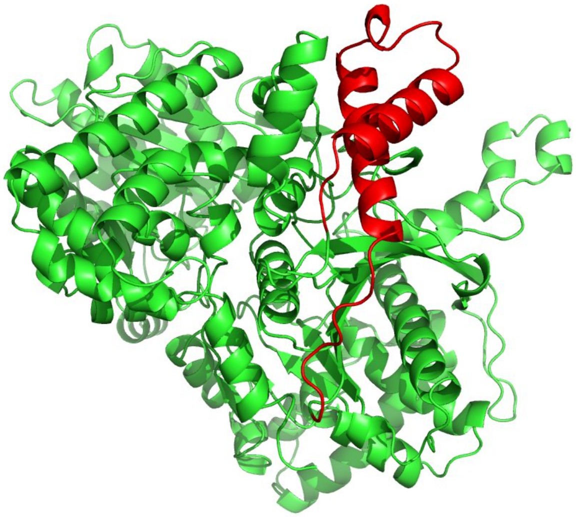

Figure S1.24: Sequence alignment and tertiary structure for gene *coaD*. The mutation cluster is highlighted in red.

E. coli K12 entry: b3634; PDB ID: 1qjc; chain ID: A

```
                  --------------------------------------------------------------------------------------XXXXXX--------
E_coli_K12        -------MQKRAIYPGTFDPITNGHIDIVTRATQMFDHVILAIAASPSKKPMFTLEERVALAQQATAHLGNVEVVGFSDLMANFARNQHATVLIRGLRAV
E_coli_CFT073     -------MQKRAIYPGTFDPITNGHIDIVTRATQMFDHVILAIAASPSKKPMFTLEERVELAQQATAHLGNVEVVGFSDLMANFARNQHATVLIRGLRAV
S_enterica_CT18   -------MQKRAIYPGTFDPITNGHLDIVTRATQMFDHVILAIAASPGKKPMFTLNERVALAQKATAHLGNVEVVGFSDLMANFARDRQANILIRGLRAV
S_enterica_Ty2    -------MQKRAIYPGTFDPITNGHLDIVTRATQMFDHVILAIAASPGKKPMFTLNERVALAQKATAHLGNVEVVGFSDLMANFARDRQANILIRGLRAV
S_boydii          -------MQKRAIYPGTFDPITNGHIDIVTRATQMFDHVILAIAASPSKKPMFTLEERVALAQQATAHLGNVEVVGFSDLMANFARNQHATVLIRGLRAV
S_dysenteriae     -------MQKRAIYPGTFDPITNGHIDIVTRATQMFDHVILAIAASPSKKTMFTLEERVELAQQATAHLGNVEVVGFSDLMANFARNQHATVLIRGLRAV
P_luminescens     ------LMKTKAIYPGTFDPVTYGHIDIVTRAADMFDHVLFAIANSARKNPMFTLDERITLAKEVTSHLDNVEVIGFCELMANFAKKQQANILIRGLRSV
Y_pestis          -------MITKAIYPGTFDPITNGHLDLVTRASAMFSHVILAIADSSSKKPMFTLDERVALAKKVTAPLKNVEVLGFSELMAEFAKKHNANILVRGLRSV
A_ferrooxidans    --MTKSDTERRVIYPGTFDPITNGHEDLVRRAAALFDEVVVAVAAQTAKTTIFPLAERVALAEATLGAIPGVRVRPFPGLLIHLLQEERAHLILRGLRAI
L_pneumophila     MQMVINEMKQKAIYPGTFDPVTNGHIDIITRASTIFPELIVAVASNKNKRPYLSWESRISLLEESVGHLTGVRVVGFDNLLIDFVLEQNAGIILRGLRAV
ruler             1.......10........20........30........40........50........60........70........80........90.......100

                  ----------------------------------------------------------------------------
E_coli_K12        ADFEYEMQLAHMNRHLMPELESVFLMPSKEWSFISSSLVKEVARHQGDVTHFLPENVHQALMAKLA----------
E_coli_CFT073     ADFEYEMQLAHMNRHLMPELESVFLMPSKEWSFISSSLVKEVARHQGDVTHFLPENVHQALMAKLA----------
S_enterica_CT18   ADFEYEMQLAHMNRHLMPQLESVFLMPSKEWSFISSSLVKEVARHQGDVTHFLPDNVHQALMDKLK----------
S_enterica_Ty2    ADFEYEMQLAHMNRHLMPQLESVFLMPSKEWSFISSSLVKEVARHQGDVTHFLPDNVHQALMDKLK----------
S_boydii          ADFEYEMQLAHMNRHLMPELESVFLMPSKEWSFISSSLVKEVARHQGDVTHFLPENVHQALMAKLA----------
S_dysenteriae     ADFEYEMQLAHMNRHLMPELESVFLMPSKEWSFISSSLVKEVARHQGDVTHFLPENVYQALMAKLA----------
P_luminescens     SDFEYEWQLANMNRHFMPELESVFLLPSQNLSFLSSSLIKDVALHDGDISSFLPEPIAQAMLKKLGK---------
Y_pestis          SDFEYEWQLANMNRHLMPKLESVFLIPSEKWSFISSSLVKEVARHGGDITPFLPKPVTKALLAKLA----------
A_ferrooxidans    SDFEHEFQLASINRRMDARIETLFLMTSDQHTFLSSSLVREISRLGGDVDAFVQPVVAAALKRHFCIGMDPGAAET
L_pneumophila     SDFEYEFQLAGMNRKLSKKVETLFLTPAEHLMYISSTLVREIAALNGDISQFVPPNVVRELKKRQNERSL------
ruler             .......110.......120.......130.......140.......150.......160.......170......
```

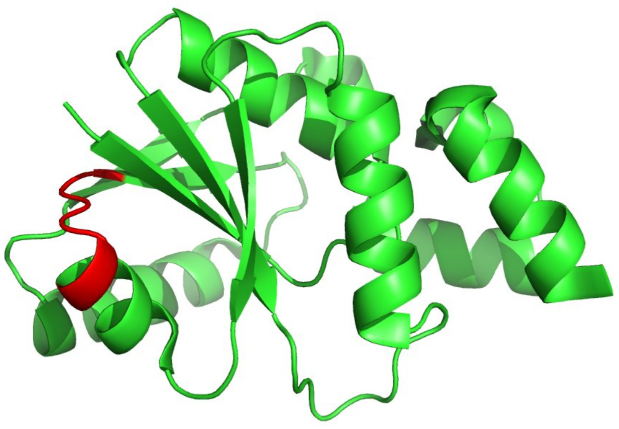

Figure S1.25: Sequence alignment and tertiary structure for gene *yicI*. The mutation cluster is highlighted in red.

E. coli K12 entry: b3656; PDB ID: 2f2h; chain ID: A

```
                -------------------------------------------------------------------------------------------------XXX
     E_coli_K12 MKISDGNWL--------IQPGLNLIHPLQVFEVEQQDNEMVVYAAPRDVRERTWQLDTPLFTLRFFSPQEGIVGVRIEHFQGALNNGPHYPLNILQDVKV
  E_coli_CFT073 MKISDGNWL--------IQPDLNLIHPLQVFEVEQQGNEMVVYAAPRDVRERTWQLDTPLFTLRFFSPQEGIVGVRIEHFQGALNNGPHYPLNILQDVKV
S_enterica_CT18 MKISDGNWL--------IQPGLNLIHPVQVFDVEQHGNEMVVYAAPRDVRERTWQLDTPLFTLRFFSPQEGVIGVRMEHFQGALDNGPHYPLNVLQDINV
 S_enterica_Ty2 MKISDGNWL--------IQPGLNLIHPVQVFDVEQHGNEMVVYAAPRDVRERTWQLDTPLFTLRFFSPQEGVIGVRMEHFQGALDNGPHYPLNVLQDINV
       S_boydii MKISDGNWL--------IQPGLNLIHPLQVFEVEQQDNEMVVYAAPRDVRERTWQLDTPLFTLRFFSPQEGIVGVRIEHFQGALNNGPHYPLNILQDVKV
       Y_pestis VMKTLKNWLLINEYPDHLELRVDDRHIFCLYVLEPNLCRVLIKREGELALSRTWSI-APQGDV----PWSGRDRLSLEGF-----SLPGYQLEKHEQQLV
          ruler 1.......10........20........30........40........50........60........70........80........90.......100

                XXXXXXXXXXXXXXXXX--XXXX-----------------------------------------------------------------------------
     E_coli_K12 TIENTERYAEFKSGNLS--ARVSKGEFWSLDFLRNGERITGSQVKNNGYVQDTNNQRNYMFERLDLGVGETVYGLGERFTALVRNGQTVETWNRDG----
  E_coli_CFT073 TIENTERYAEFKSGNLS--ARVSKGEFWSLDFLRNGERITGSQVKNNGYVQDTNNQRNYMFERLDLGVGETVYGLGERFTALVRNGQTVETWNRDG----
S_enterica_CT18 EMQNNAEFAELKSGSLS--VRVTKGELWSLDFLRNGVRITGSQLKNNGYVQDTNSGRNYMFERLDLGVGETVYGLGERFTALVRNGQTVETWNRDG----
 S_enterica_Ty2 EMQNNAEFAELKSGSLS--VRVTKGELWSLDFLRNGVRITGSQLKNNGYVQDTNSGRNYMFERLDLGVGETVYGLGERFTALVRNGQTVETWNRDG----
       S_boydii TIENTERYAEFKSGNLS--ARVSKGEFWSLDFLRNGERITG---------QDTNNQRNYMFERLDLGVGETVYGLGERFTALVRNGQTVETWNRDG----
       Y_pestis VTTECLRVTIHQPLHLTWEYKNPHGEWLPL----AADRPTSAYLLSPQGEAIAHYQRRYPNEQY--------YGLGEKAGDLNRAGRRFEMRNLDAMGYN
          ruler .......110.......120.......130.......140.......150.......160.......170.......180.......190.......200

                ----------------------------------------------------------------------------------------------------
     E_coli_K12 GTSTEQAYKNIPFYMTNR---GYGVLVNHPQCVSFEVGSE----KVSKVQFSVESEYLEYFVIDGPTPKAVLDRYTRFTGRPALPPAWSFGLWLTTSFTT
  E_coli_CFT073 GTSTEQAYKNIPFYMTNR---GYGVLVNHPQCVSFEVGSE----KVSKVQFSVESEYLEYFVIDGPTPKAVLDRYTRFTGRPALPPAWSFGLWLTTSFTT
S_enterica_CT18 GTSTEQSYKNIPFYITNR---GYGVLVNHPQCVSFEIGSE----KVSKVQFSVESEYLEYFVIDGPTPKDVLNRYTQFTGRPALPPAWSFGLWLTTSFTT
 S_enterica_Ty2 GTSTEQSYKNIPFYITNR---GYGVLVNHPQCVSFEIGSE----KVSKVQFSVESEYLEYFVIDGPTPKDVLNRYTQFTGRPALPPAWSFGLWLTTSFTT
       S_boydii GTSTEQAYKNIPFYMTNR---GYGVLVNHPQSVSFEVGSE----KVSKVQFSVESEYLEYFVIDGPTPKAVLDRYTRFTGRPALPPAWSFGLWLTTSFTT
       Y_pestis AASTDPLYKHIPFTITRRDDVSFGLFYDNLSSCWLDLGNEIDNYHLAYRRYQAEAGDLDYYLFLGPKVLDVTKAFVRLTGKTQFGPKWSLGYSGSTMHYT
          ruler .......210.......220.......230.......240.......250.......260.......270.......280.......290.......300

                ----------------------------------------------------------------------------------------------------
     E_coli_K12 AESLRGGLSIGLSGFGFWSHDIGGFENTAP-AHVYKRWCAFGLLSSHSRLHG---SKSYRVPWAYDDESCDVVRFFTQLKCRMMPYLYREAARANARGTP
  E_coli_CFT073 AESLRGGLSIGLSGFGFWSHDIGGFENTAP-AHVYKRWCAFGLLSSHSRLHG---SKSYRVPWAYDDESCDVVRFFTQLKCRMMPYLYREAARANARGTP
S_enterica_CT18 AESLRGGLSIGLSGFGFWSHDIGGFENTAP-AHVYKRWCAFGLLSSHSRLHG---SKSYRVPWAYDDESCDVVRFFTEQKCRMMPYLYREAARANEAGTP
 S_enterica_Ty2 AESLRGGLSIGLSGFGFWSHDIGGFENTAP-AHVYKRWCAFGLLSSHSRLHG---SKSYRVPWAYDDESCDVVRFFTEQKCRMMPYLYREAARANEAGTP
       S_boydii AESLRGGLSIGLSGFGFWSHYIGGFENTAP-AHVYKCWCAFGLLSSHSRLHG---SKSYRVPWAYDDESCDVVRFFTQLKCRMMPYLYREAARANARGTP
       Y_pestis RYNIRMGLGMSLSGLYNLGHDVGGFSGDKPEPELFVRWVQNGVMHPRFTIHSWNDDNTVNEPWMY-PAATPMIRDAMALRYRLLPYFYTLQWQASHDDEP
          ruler .......540.......550.......560.......570.......580.......590.......600.......610.......620.......630

                ----------------------------------------------------------------------------------------------------
     E_coli_K12 MMRAMMMEFPDDPACDYLDRQYMLGDNVMVAPVFTEAG--DVQFYLPEGR--WTHLWHNDELDGSRWHKQQHGFLSLPVYVRDNTLLALGNNDQRPDYVW
  E_coli_CFT073 MMRAMMMEFPDDPACDYLDRQYMLGDNVMVAPVFTEAG--DVQFYLPEGR--WTHLWHNDELDGSRWHKQQHSFLSLPVYVRDSTLLALGNNDQRPDYAW
S_enterica_CT18 MMRAMMLEFPDDPACDYLDRQYMLGDAVMVAPVFSEAG--DVEFYLPEGR--WTHLWRNDEVQGSRWHKQQHDFLSLPVYVRDNTLLALGNNSQKPDYAW
 S_enterica_Ty2 MMRAMMLEFPDDPACDYLDRQYMLGDAVMVAPVFSEAG--DVEFYLPEGR--WTHLWRNDEVQGSRWHKQQHDFLSLPVYVRDNTLLALGNNSQKPDYAW
       S_boydii MMRAMMMEFPDDPACDYLDRQYMLGDNVMVAPVFTEAG--DVQFYLPEGR--WTHLWHNDELDGRCWHKQQHSFLSLPIYVRDNTLLALGNNDQRPDYAW
       Y_pestis MLRPTFLDHEHDSLTFKENDDFMLGRDLLVASVV-DAGQRQRQIYLPDNQVGWYCFHSGQWYSGGQTITLDAPLERLPLLVRAGAALPL---SRRIAFVN
          ruler .......640.......650.......660.......670.......680.......690.......700.......710.......720.......730

                ----------------------------------------------------------------------XXXXXXX-----------------------
     E_coli_K12 HEGTA---FHLFNLQDGHEAVCEVPAADG---------SVIFTLKAARTGNTITVTGAGEAKNWTLCLRNVVKVNGLQDGSQAESEQGLVVKPQGNALTI
  E_coli_CFT073 HEGTA---FHLFNLQDGHEAVCEVPDADG---------SVIFTLKAARTGNTITVTGTGEAKNWTLCLRNIVKVNGLQGGSQAESEQGLVVTPQGNALTI
S_enterica_CT18 HEGTA---FQLFHLDDGCEAVCEVPATDG---------STIFTLQAKRTGNTITVSGEGEARNWTLCLRNITQISGTKCGSYAGSELGVVVTPQGNEVVI
 S_enterica_Ty2 HEGTA---FQLFHLDDGCEAVCEVPATDG---------STIFTLQAKRTGNTITVSGEGEARNWTLCLRNITQISGTKCGSYAGSELGVVVTPQGNEVVI
       S_boydii HEGTA---FHLFNLQDGHEAVCEVPAADG---------SVIFTLKAARTGNTITVTGAGEAKNWTLCLRNVVKVNGLQDGSQAESEQGLVVKPQGNALTI
       Y_pestis PEADCQRELALYPTQGGGQSSGMLFEDDGESHRWQQGHALWLNWQVTTDNHRIDIT-FSRTGNYQPAWRELT-INLPTNESRTLSINGVA----GNTLDL
          ruler .......740.......750.......760.......770.......780.......790.......800.......810.......820.......830
```

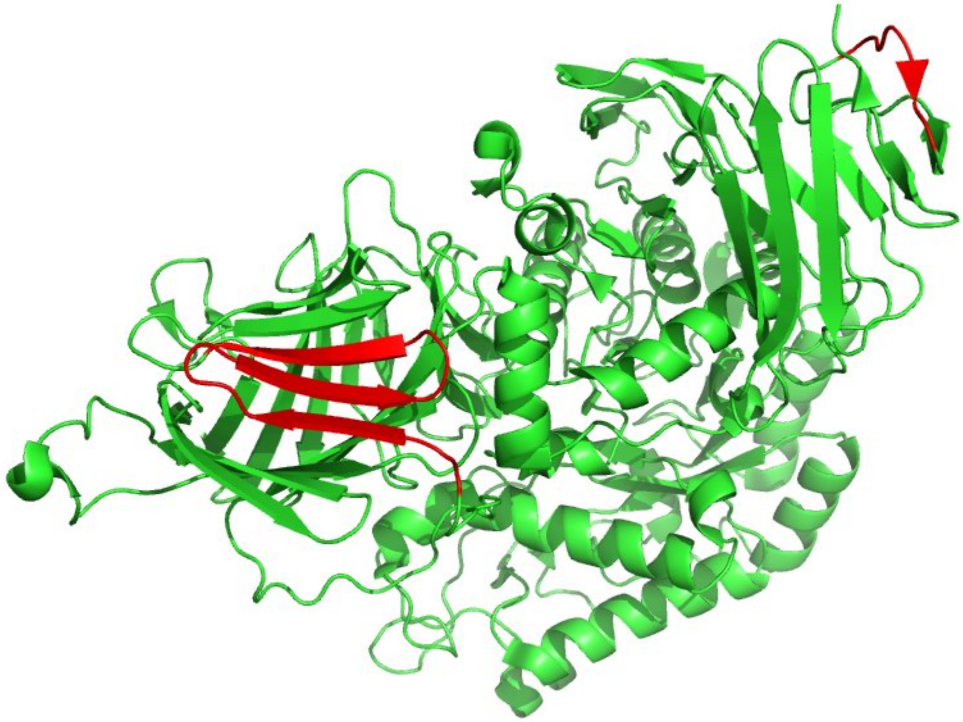

Figure S1.26: Sequence alignment and tertiary structure for gene *ubiC*. The mutation cluster is highlighted in red.

E. coli K12 entry: b4039; PDB ID: 1tt8; chain ID: A

```
                -------------------------------------------------------------------------------------------XXXXXXXXX
      E_coli_K12 MRLLRFCCVLDHLICFTSPVNTFLRYNAFTLCNGEFGMSHPALTQLRALRYCKEI-PALDPQLLDWLLLEDSMTKRFEQQGKTVSVTMIREGFVEQNEIP
   E_coli_CFT073 -------------------------------------MSHPALTQLRALRYFTEI-PALEPQLLDWLLLEDSMTKRFEQQGKTVSVTMIREGFVEQNEIP
S_enterica_CT18 -------------------------------------MSHPALTQLRALRYFDAI-PALEPHLLDWLLLEDSVTKRFEQQGKRVSVTLIREAFVGQSEVE
 S_enterica_Ty2 -------------------------------------MSHPALTQLRALRYFDAI-PALEPHLLDWLLLEDSVTKRFEQQGKRVSVTLIREAFVGQSEVE
        S_boydii MRLLRFCCALDHLICFTSPVNTFLRYNAFTLCNGEFGMSHPALTQLRALRYFKEI-PALDPQLLDWLLLEDSMTKRFEQQGKTVSVTMIREGFVEQNEIP
   S_dysenteriae MRLLRFCCALDHLICFTSPVNTFLRYNAFTLCNGEFGMSHPALTQLRALRYFKEI-PALEPQLLDWLLLEDSMTKRFEQQGKTVSVTMIREGFVEQNEIP
   P_luminescens ---------------------------------MEKRMSTDSILTTVPIQWLSVDSPVLPDEVLDWLMELGSMTRRFEQYCNSVRIIPFRECFITEEQLS
        Y_pestis -------------------------------------MFIGDASILKPIQWCATEHPELPADIADWLMELGSMTRRFEQHCQRVHVEPQRECFITRDALG
           ruler 1.......10........20........30........40........50........60........70........80........90.......100

                XXXX------------------------------------------------------------------------------------------------
      E_coli_K12 EELPLLPKESRYWLREILLCADGEPWLAGRTVVPVSTLSGPELALQKLGKTPLGRYLFTSSTLTRDFIEIGRDAGLWGRRSRLRLSGKPLLLTELFLPAS
   E_coli_CFT073 EELPLLPKESRYWLREILLCADGEPWLAGRTVVPVSTLSGPELALQKLGKTPLGRYLFTSSTLTRDFIEIGRDAGLWGRRSRLRLSGKPLLLTELFLPAS
S_enterica_CT18 EASGLLPSESRYWLREILLCADGEPWLAGRTVVPESTLCGPEQVLQHLGKTPLGRYLFTSSTLTRDFIEIGRDATLWGRRSRLRLSGKPLLLTELFLPAS
 S_enterica_Ty2 EASGLLPSESRYWLREILLCADGEPWLAGRTVVPESTLCGPEQVLQHLGKTPLGRYLFTSSTLTRDFIEIGRDATLWGRRSRLRLSGKPLLLTELFLPAS
        S_boydii EELPLLPKESRYWLREILLCADGEPWLAGRTVVPVSTLSGPELALQKLGKTPLGRYLFTSSTLTRDFIEIGRDAGLWGRRSRLRLSGKPLLLTELFLPAS
   S_dysenteriae EELPLLPKESRYWLREILLCADGEPWLAGRTVVPVSTLSGPELALQKLGKTPLGRYLFTSSTLTRDFIEIGRDAGLWGRRSRLRLSGKPLLLTELFLPAS
   P_luminescens DENERLLTGQRYWLREIVLCGDNIPWLLGRTLIPETTLTGPDESLVDLGTVPLGRYLFSGNKLTRDYIHVGQQGNRWARRSLLRLSGKPLLLTEVFLPES
        Y_pestis EEAEHLPVSQRYWLREIVLFGDNVPWLLGRTVIPEETLSGPDRALVDLGTLPLGRYLFSGDALTRDYIHVGRQDNLWARRSLLRLSGNPLLLTEVFLPAS
           ruler .......110.......120.......130.......140.......150.......160.......170.......180.......190.......200

                -----------
      E_coli_K12 PLY--------
   E_coli_CFT073 PLY--------
S_enterica_CT18 PLY--------
 S_enterica_Ty2 PLY--------
        S_boydii PLY--------
   S_dysenteriae PLY--------
   P_luminescens PVYKR------
        Y_pestis PLYTHCDSIPK
           ruler .......210.
```

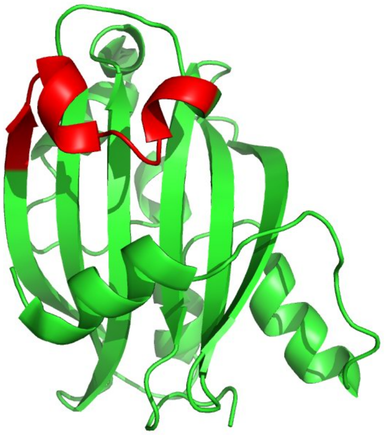

Figure S1.27: Sequence alignment and tertiary structure for gene *frdA*. The mutation cluster is highlighted in red.

E. coli K12 entry: b4154; PDB ID: 1kf6; chain ID: A

```
                  ----------------------------------------------------------------------------------------------------
      E_coli_K12  ELGPRDKVSQAFWHEWRKGNTISTPRGDVVYLDLRHLGEKKLHERLPFICELAKAYVGVDPVKEPIPVRPTAHYTMGGIETDQNC---------ETRIKG
   E_coli_CFT073  ELGPRDKVSQAFWHEWRKGNTISTPRGDVVYLDLRHLGEKKLHERLPFICELAKAYVGVDPVKEPIPVRPTAHYTMGGIETDQNC---------ETRIKG
S_enterica_CT18   ELGPRDKVSQAFWHEWRKGNTISTPRGDVVHLDLRHLGEKKLHERLPFICELAKAYVGVDPVKEPIPVRPTAHYTMGGIETDQNC---------ESRVKG
 S_enterica_Ty2   ELGPRDKVSQAFWHEWRKGNTISTPRGDVVHLDLRHLGEKKLHERLPFICELAKAYVGVDPVKEPIPVRPTAHYTMGGIETDQNC---------ESRVKG
        S_boydii  ELGPRDKVSQAFWHEWRKGNTISTPRGDVVYLDLRHLGEKKLHERLPFICELAKAYVGVDPVKEPIPVRPTAHYTMGGIETDQNC---------ETRIKG
   S_dysenteriae  ELGPRDKVSQAFWHEWRKGNTISTPRGDVVYLDLRHLGEKKLHERLPFICELAKAYVGVDPVKEPIPVRPTAHYTMGGIETDQNC---------ETRIKG
   P_luminescens  ELGPRDKVSQAFWHEWRAGRTISTPRGNVVYLDLRHLGEKKLLERLPFICELAKAYVGVDPVKEPIPVRPTAHYTMGGIETDQKC---------ETRIKG
        Y_pestis  ELGPRDKVSQAFWHEWRAGRTVATPRGDVVYLDLRHLGEKKLLERLPFICELAKAYVGVDPVKEPIPVRPTAHYTMGGIETNPQC---------ETRIKG
  A_ferrooxidans  ELAPRDIVARAIDLEMKQ------HQLDYVTLDISAQPATLVRRHFPAIFAHCRA-QGYDLTRESIPVVPAAHYSCGGVVVDEAG---------RSTIPG
   L_pneumophila  DLASRDVVARAMALEIRAGKGFDPKGVDHVKLKLDHLGADLIMSRLPGIRELSMKFAGVDPIVEPIPVVPTCHYSMGGIPTNMHGQVITKTDGKEHVVEG
           ruler  .......310.......320.......330.......340.......350.......360.......370.......380.......390.......400

                  ----------------------------------------------XXXXXXXXXXXXXXXXXXXXXXXXXXXXXXX-----------------------
      E_coli_K12  LFAVGECSSVGLHGANRLGSNSLAELVVFGRLAGEQATERAATAG-NGNEAAIEAQAAGVEQRLKDLVNQDGGENWAKIRDEMGLAMEEGCGIYRTPELM
   E_coli_CFT073  LFAVGECSSVGLHGANRLGSNSLAELVVFGRLAGEQATERAATAG-NGNEAAIEAQAAGVEQRLKDLVNQDGGENWAKIRDEMGMAMEEGCGIYRTPELM
S_enterica_CT18   LFAVGECSSVGLHGANRLGSNSLAELVVFGRLAGEQAMERAATAG-AANSAALDAQVADIEQRLKNLVNQEGNENWSKIRDEMGLSMEEGCGIYRTPELM
 S_enterica_Ty2   LFAVGECSSVGLHGANRLGSNSLAELVVFGRLAGEQAMERAATAG-AANSAALDAQVADIEQRLKNLVNQEGNENWSKIRDEMGLSMEEGCGIYRTPELM
        S_boydii  LFAVGECSSVGLHGANRLGSNSLAELVVFGRMAGEQATERAATAG-NGNEEAIEAQAAGVEQRLKDLVNQDGGENWAKIRDEMGLAMEEGCGIYRTPELM
   S_dysenteriae  LFAVGECSSVGLHGANRLGSNSLAELVVFGRLAGEQATERAATAG-NGNEAAIEAQAAGVEQRLKDLVNQDGGENWAKIRDEMGLAMEEGCGIYRTPELM
   P_luminescens  LFAVGECSSIGLHGANRLGSNSLAELVVFGRLAGEEAVKHVQQTK-PINDRTLDAQACDVVARLNNLLKQKGTESWSKIRDEMGISMEEGCGIYRTPELM
        Y_pestis  LFAVGECSSVGLHGANRLGSNSLAELVVFGRVAGEQAALRAMESG-PANGSALDAQTRDVETRLSNLMKQEGTENWSKIRDEMGLSMEEGCGIYRTPELM
  A_ferrooxidans  LYAAGEVSSTGLHGANRLASNSLLECVV---GAAAIVADLEGQARLPDRPRQTPGRSPGQPTTAARRTDINA------LRQNLQVNMWQAAGIVRNDEGL
   L_pneumophila  LYAVGECACVSVHGANRLGGNSLLDLVVFGRAAGIHVEELWQSNQLPDMSYVSEDDVVASLVRYERWENSKDGESHAVIRDEMQRVMQEDFGVFRTGEVM
           ruler  .......410.......420.......430.......440.......450.......460.......470.......480.......490.......500

                  ----------------------------------------------------------------------------------------------------
      E_coli_K12  QKTIDKLAELQERFKRVRITDTSSVFNTDLLYTIELGHGLNVAECMAHSAMARKESRGAHQRLDEGCTERDDVNFLKHTLAFRDADGTTRLEYSDVK---
   E_coli_CFT073  QKTIDKLAELQERFKRVRITDTSSVFNTDLLYTIELGHGLNVAECMAHSAMARKESRGAHQRLDEGCTERDDVNFLKHTLAFRDADGTTRLEYSDVK---
S_enterica_CT18   QKTVDKLAELQERFKRVRISDTSSVFNTDLLYTIELGHGLNVAECMAHSALARKESRGAHQRLDEGCTERDDVNFLKHTLAFRDADGTTRLEYSDVK---
 S_enterica_Ty2   QKTVDKLAELQERFKRVRISDTSSVFNTDLLYTIELGHGLNVAECMAHSALARKESRGAHQRLDEGCTERDDVNFLKHTLAFRDADGTTRLEYSDVK---
        S_boydii  QKTIDKLAELQERFKRVRITDTSSVFNTDLLYTIELGHGLNVAECMAHSAMARKESRGAHQRLDEGCTERDDVNFLKHTLAFRDADGTTRLEYSDVK---
   S_dysenteriae  QKTIDKLAELQERFKRVRITDTSSVFNTDLLYTIELGHGLNVAECMAHSAMARKESRGAHQRLDEGCTERDDVNFLKHTLAFRDADGTTRLEYSDVK---
   P_luminescens  LKTIDKIAELKDRFKRVKITDNSNVFNTDLLYTIELGFSLDVAECMIHSAINRKESRGAHQRLDEGCTERDDEKCLKHTLAFYDPESVPHLEYSDVK---
        Y_pestis  QKTVDKLAELKERFKRVKITDHSSVFNTDLLYTIELGYGLDVAECMAHSALNRKESRGAHQRLDEGCTERDDVNFLKHTLAFHTPGGAPRIEYSDVK---
  A_ferrooxidans  RAACAYWDE--------RGTHISDSLCGPQPYQ-ELRNLCQCAKILTCAALLREESRGCHFN--SDHPQRRAV--AADSISQRDQQ-------SPF----
   L_pneumophila  ASGLKRLQALRDRLAHAKLDDKSQIFNTERVEAMELDNLMATAYATALSAIARTESRGAHSR--EDYPKRDDANWIKHTLYFEEGE---VIDYRPVNTSP
           ruler  .......510.......520.......530.......540.......550.......560.......570.......580.......590.......600
```

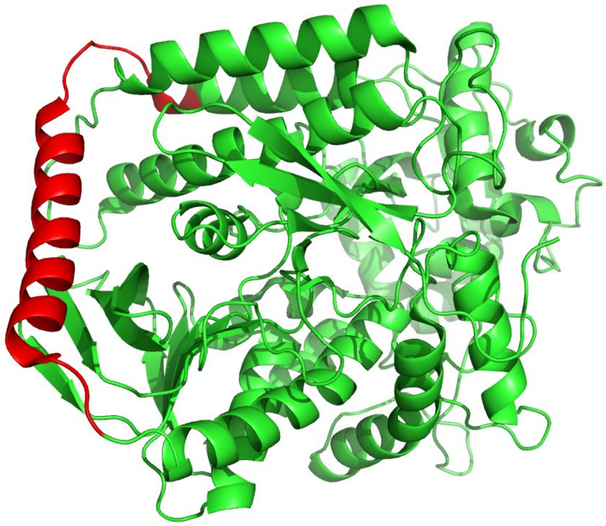

Figure S1.28: Sequence alignment and tertiary structure for gene *treC*. The mutation cluster is highlighted in red.

E. coli K12 entry: b4239; PDB ID: 1uok; chain ID: -

```
                ----------------------------------------------------------------------------------------------------
      E_coli_K12 SSTSLEHCQRYAALTGSELSMTFNFHHLKVDYPGGEKWTLAKPDFVALKTLFRHWQQGMHNVAWNALFWCNHDQPRIVSRFGDEGEYRVPAAKMLAMVLH
   E_coli_CFT073 SSTSLEHCQRYAALTGSELSMTFNFHHLKVDYPGGEKWTLAKPDFVALKTLFRHWQQGMHNVAWNALFWCNHDQPRIVSRFGDEGEYRVPAAKMLAMVLH
 S_enterica_CT18 SSTTLENCQQYAALSGDELSMTFNFHHLKVDYPNGEKWTLAKPDYVALKALFRQWQQGMHNVAWNALFWCNHDQPRIVSRFGDEGEYRVPAAKMLAMALH
  S_enterica_Ty2 SSTTLENCQQYAALSGDELSMTFNFHHLKVDYPNGEKWTLAKPDYVALKALFRQWQQGMHNVAWNALFWCNHDQPRIVSRFGDEGEYRVPAAKMLAMALH
        S_boydii SSTSLEHCQRYAALTGSELSMTFNFHHLKVDYPGGEKWTLAKPDFVALKTLFRHWQQGMHNVAWNALFWCNHDQPRIVSRFGDEGEYRVPAAKMLAMVLH
   S_dysenteriae SSTSLEHCQRYAALTGSELSMTFNFHHLKVDYPGGEKWTLAKPDFVALKTLFRHWQQGMHNVAWNALFWCNHDQPRIVSRFGDEGEYRVPAAKMLAMVLH
   P_luminescens SSTSLEHCHRYSSLDGEELSMVFAFHHLKVDYPNGEKWVAAPPDFIELKKIFAKWQQGMYERAWNALFWCNHDQPRIVSRFGGEGVWRVPSAKMLAMVLH
        Y_pestis SSTRLEHCQRYAALGGDELSMTFNFHHLKVDYLNGEKWSLMPPNRVELKHIFNQWQQGMHNRAWNALFWCNHDQPRIVSRFGHEGALRLPAAKMLAMVLH
           ruler .......270.......280.......290.......300.......310.......320.......330.......340.......350.......360

                ----------------------------------------------------------------------------------------------------
      E_coli_K12 GMQGTPYIYQGEEIGMTNPHFTRITDYRDVESLNMFAE-LRNDGRDADELLAILASKSRDNSRTPMQWSNGDNAGFTAGEPWIGLGDNYQQINVEAALAD
   E_coli_CFT073 GMQGTPYIYQGEEIGMTNPHFTRITDYRDVESLNMFAE-LRNDGRDADELLAILASKSRDNSRTPMQWTNGDNAGFTAGEPWIGLGDNYQEINVEAALAD
 S_enterica_CT18 GMQGTPYIYQGEEIGMTNPHFTRITDYRDVESHNMFAA-LRAAGRDPDELLAILASKSRDNSRTPMQWDNGKNAGFTQGEPWISLCDNYTEVNVAAALRD
  S_enterica_Ty2 GMQGTPYIYQGEEIGMTNPHFTRITDYRDVESHNMFAA-LRAAGRDPDELLAILASKSRDNSRTPMQWDNGKNAGFTQGEPWISLCDNYTEVNVAAALRD
        S_boydii GMQGTPYIYQGEEIGMTNPHFTRITDYRDVESLNMFAE-LRNDGRDADELLAILASKSRDNSRTPMQWSNGDNAGFTAGEPWIGLGDNYQQINVEAALAD
   S_dysenteriae GMQGTPYIYQGEEIGMTNPHFTRITDYRDVESLNMFAE-LRNDGRDADELLAILASKSRDNSRTPMQWSNGDNAGFTAGEPWISLGDNYQEINVEAALTD
   P_luminescens GMQGTPYIYQGEELGMTNPHFSHIEEYRDIESLNIYQE-KIEQGNDKQQILSVLAKKSRDNGRTPMQWDTTANAGFTQGEPWIALNSDYQDVNAENAMQD
        Y_pestis GMQGTPYIYQGEEIGMTNPNFTDITQYRDVESLNMFAE-LSVAGREPDELLAILAAKSRDNSRTPMQWDSSDNAGFSQGTPWIAPCSNYHEINVNAALAD
           ruler .......370.......380.......390.......400.......410.......420.......430.......440.......450.......460

                ----------------XXXXXXXXXXXXXXXXXXXXXXXXXXXXXXXXXXXXXXXXXXXXXXXXXXX--XXXXXXXXXXXXXXXXXX-------------
      E_coli_K12 DSSVFYTYQKLIALRKQEAILTWGNYQDLLPNSPVLWCYRREWKGQTLLVIANLSREIQPWQAGQMR--GNWQLVMHNYEEASPQPCAMNLRPFEAVWWL
   E_coli_CFT073 ESSVFYTYQKLIALRKQEAVLTWGDYQDLLPNSPVLWCYRREWKGQTLLVIANLSRETQPWQPGKML--GNWQLVMHNYEEASPQPCTMTLRPFEAVWWL
 S_enterica_CT18 ENSVFYTYQKLIALRKTQPVMIWGDYQDLLPGSPSVWCYRRQWQGQTLLVVANLSDQCQEWHPPHIK--GQWQALLHNYGEVASQPAAMTLRPFEAIWWL
  S_enterica_Ty2 ENSVFYTYQKLIALRKTQPVMIWGDYQDLLPGSPSVWCYRRQWQGQTLLVVANLSDQCQEWHPPHIK--GQWQALLHNYGEVASQPAAMTLRPFEAIWWL
        S_boydii DSSVFYTYQKLIALRKQEAILTWGNYQDLLPNSPVLWCYRREWKGQTLLVIANLSREIQPWQPGQMR--GNWQLVMHNYEEASPQPCAMNLRPFEAVWWL
   S_dysenteriae ESSVFYTYQKLIALRKQEAVLTWGNYQDLLPNSPVLWCYRREWKGQTLLVIANLSREIQPWQPEQMR--GNWQLVMHNYEEASPQPCAMTLQPFEAVWWL
   P_luminescens DDSVFHCYARLIKLRKEYPVLTYGSYEDLLPSHPSLWCYVRRWENQTLLVIANLSVEVQKWSLDASLSDCSWVKLMGNYPENHVPAKDMQLQPYEAVYWL
        Y_pestis ADSVFYAYQYLIALRKQYDIFTFGDYQDLCPQHPDLWCYLRSWQGKQLLVVANLSEEPQRWQPEGLTLDGNWQLLMSSYDQSAFQPQDMILRGYEGIYWV
           ruler .......470.......480.......490.......500.......510.......520.......530.......540.......550.......560
```

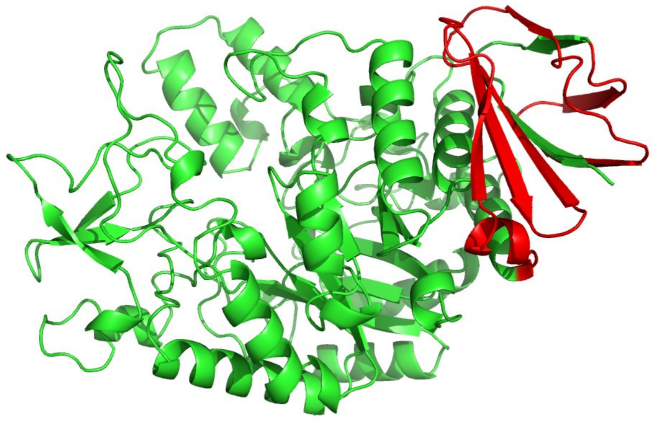

Figure S1.29: Sequence alignment and tertiary structure for gene *yjjV*. The mutation cluster is highlighted in red.

E. coli K12 entry: b4378; PDB ID: 1zzm; chain ID: A

```
                 -------------------------------------------------------XXXXXXXXXXXXXXXXXXXXXXXXXXXXXXXXXXXXXXXXXXXXX
       E_coli_K12 ---------------------------------------------------VLALAENYQPLYAALGLHPGMLEKHSDVSLEQLQQALERRPAKVVAVGE
   E_coli_CFT073 LICRFIDTHCHFDF---PPFSGDEEASLQRAAQAGVGKIIVPATEAENFARVQALAEKYQPLYAALGLHPGMLEKHSDVSLDQLQQALERHPAKVVAVGE
S_enterica_CT18 VSWRFIDTHCHFDF---PPFTGDERASIQRACEAGVEKIIVPATEAAHFPRVLALAARFPSLYAALGLHPIVIERHADDDPDKLQQALAQQQN-VVAVGE
 S_enterica_Ty2 VSWRFIDTHCHFDF---PPFTGDERASIQRACEAGVEKIIVPATEAAHFPRVLALAARFPSLYAALGLHPIVIERHADDDPDKLQQALAQQQN-VVAVGE
       S_boydii LICRFIDTHCHFDF---LPFSGDEEASLQRAAQAGVGKIIVPATEAENFARVQALAEKYQPLYAALGLHPGMLEKHSDVSLEQLQQALERRPAKVVAVGE
  S_dysenteriae LICRFIDTHCHFDF---LPFSGDEEASLQRAAQAGVGKIIVPATEAENFARVQALAEKYQPLYAALGLHPGMLEKHSDVSLDQLQQALERRPAKVVAVGE
  P_luminescens ---MFIDTHCHFDF---PPFRDDEQQSLQRAAQVGVDRIIVPAISSANLQQVSELACSYPAIYAALGLHPLYIDQHQEAHLADLESFLKQKHEKLVAVGE
       Y_pestis -MPYFIDTHCHFDF---PPFTGDEAASLACAAEANVRQLIVPSVKAAYFSRILALADRYPPLFAALGLHPLYIAEHEDADLAALASHLADKPPKLVAIGE
 A_ferrooxidans -MHGLIDSHCHLDD---AAFDDDRDAVLCRAGMAGVRKMVLPAYTPKYWGRLRETCRRWPGLYPAYGVHPLYLEDRDGQWEATLASFLAD----AVALGE
  L_pneumophila ---VLVDSHCHLNFIDLTHFHHDLAQVINQARENEVEHFLSVCVELSDYPQLERLAKDYPDISISVGVHPN--SEMNYPVTAQMLCELAQNPA-CIAIGE
          ruler 1.......10........20........30........40........50........60........70........80........90.......100

                XXXXXXXXXXXXXX--XXXXXXXXXX--------------------------------------------------------------------------
       E_coli_K12 IGLDLFGDDPQFER--QQWLLDEQLKLAKRYDLPVILHSRRTHDKLAMHLKRHDLPR-TGVVHGFSGSLQQAERFVQLGYKIGVGGTITYPRASKTRDVI
   E_coli_CFT073 IGLDLFGDDPQFER--QQWLLDEQLKLAKRYDLPVILHSRRTHDKLAMHLKRHDLPC-TGVVHGFSGSLQQAERFVQLGYKIGVGGTITYPRASKTRDVI
S_enterica_CT18 IGLDLYRDDPQFAR--QERFLDAQLQLAKRYDLPVILHSRRTHDKLAMRLKRQSLPQ-TGVVHGFSGSLQQAERFVQLGYKIGVGGTITYPRASKTRDVM
 S_enterica_Ty2 IGLDLYRDDPQFAR--QERFLDAQLQLAKRYDLPVILHSRRTHDKLAMRLKRQSLPQ-TGVVHGFSGSLQQAERFVQLGYKIGVGGTITYPRASKTRDVM
       S_boydii VGLDLFGDDPQLER--QQWLLDEQLKLAKRYDLPVILHSRRTHDKLAMHLKRHDLSR-TGVVHGFSGSLQQAERFVQLGYKIGVGGTITYPRASKTRDVI
  S_dysenteriae SGLDLFGDDPQFER--QQWLLDEQLKLAKRYDLPVILHSRRAHDKLAMHLKRHDLSR-TGVVHGFSGSLQQAERFVQLGYKIGVGGTITYPRASKTRDVI
  P_luminescens IGLDLYMTEPQFEK--QKALLCDQLRLAKQYELPVILHSRKSHDQLAAILRRINLHR-TGVVHGFAGSLSQAEAFIGLGYYLGVGGTITYERAQKTRRVM
       Y_pestis IGLDLYMDEPQFPH--QLVILNMQLELAKQHDLPVILHSRRSHDPLAAALRKAALPR-AGVIHGFAGSLAQAQAFIRLGYYIGVGGTITYKRAQKTRHVM
 A_ferrooxidans IGLDASAGTPDAAC--QRAGFTTQLAMAQQLGLPVILHARQNLEEMILILRR--FPRLRGVLHSFSGSPVQARRLTDMGFLLGLGGALTHPRARRLRAIA
  L_pneumophila TGLDYYRNPTEEAQSLQRDRFREHIKAALITSKPLIIHTRQAAEDTLLMMAKEGASQIGGVMHCFAEDLDIAQRAIDLNFYISFSGIVTFKNAVSLQDVA
          ruler .......110.......120.......130.......140.......150.......160.......170.......180.......190.......200

                ---------------------------------------------------------------------
       E_coli_K12 AKLPLASLLLETDAPDMPLNGFQGQPNRPEQAARVFAVLCELRREPADEIAQALLNNTYTLFNVP----
   E_coli_CFT073 AKLPLASLLLETDAPDMPLNGFQGQPNRPEQAARVFAVLCELRPEPADEIAEVLLNNTYTLFNVP----
S_enterica_CT18 ARLPLDALLLETDAPDMPLKGFQGQPNRPEQAARVFDALCELRPEPAEVIADTLYRNTVTLFRL-----
 S_enterica_Ty2 ARLPLDALLLETDAPDMPLKGFQGQPNRPEQAARVFDALCELRPEPAEVIADTLYRNTVTLFRL-----
       S_boydii AKLPLASLLLETDAPDMPLNGFQGQPNRPEQAVRVFDVLCELRPEPEDEIAEVLLNNTYALFSVSG---
  S_dysenteriae AKLPLASLLLETDAPDMPLNGFQGQPNRPEQAVRVFDVLCELRPEPEDEIAEVLLNNTYALFSVSG---
  P_luminescens AELPLTSLVLETDAPDMPLFGFQGEPNRPEKIAGVFASLCELRREAPDEIARQLYHNSVELFGLII---
       Y_pestis ASLPLSSLLLETDAPDMPLASFQGQANRPERAANVFAALCELRPEPADEIAAELMCNSQRLFSLPPLRP
 A_ferrooxidans ATLPAEFLLAETDAPWQPPHAHPGARNEPAYLREIIADIAQLRHTSVQEIARITAQNALSLFHLQDVS-
  L_pneumophila RQIPLERILIETDSPYLAPVPHRGKQNHPALVKHVAEAIAQLRGMDFQEIAEITTNNFYNCFNIKRQG-
          ruler .......210.......220.......230.......240.......250.......260.........
```

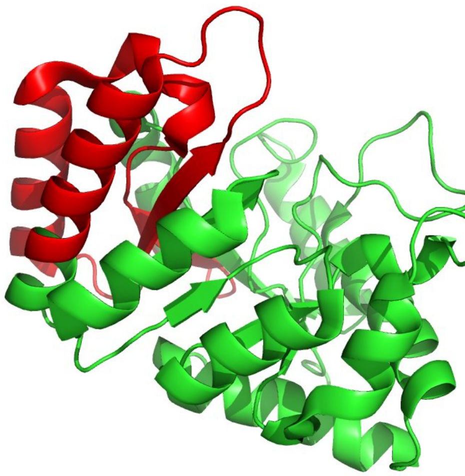

Figure S1.30: Sequence alignment and tertiary structure for gene *wbl*. The mutation cluster is highlighted in red.

D. melanogaster FlyBase ID: FBgn0004003; PDB ID: 1ovn; chain ID: B

```
                ----------------------------------------------------------------------------------------------------
 D_melanogaster MMHILVTL--LLVAI--HSIPTTW-AVTCTGCVDLDELSFEKTVERFPYSVVKFDIAYPYGEKHEAFTAFSKSAHKATKDLLIATVGVKDYGELENKALG
    D_sechellia MMHILVTL--LLVAC--HSIPTTW-AVTCTGCVDLDELSFQKTVERFPYSIVKFDIAYPYGEKHEAFTAFSKSAHKATKDLLIATVGVKDYGELENKALG
     D_simulans MMHILVTL--LLVAC--HSIPTTW-AVTCTGCVDLDELSFQKTVERFPYSIVKFDIAYPYGEKHEAFTAFSKSAHKATKDLLIATVGVKDYGELENKALG
       D_yakuba MMHILVTL--LLVAF--HAIPSTW-AVTCTGCVDLDELSFQQTVERFPYSIVKFDIAYPYGEKHEAFTAFSKSAHKATKDLLIATVGVKDYGELENKALG
       D_erecta MMHILVTL--LLVAF--HSLPSTW-AVTCPGCVDLDELSFQQAVERFPYSIVKFDIAYPYGEKHEAFTAFSKSAHKATQDLLIATVGVKDYGELENKALG
    D_ananassae MMRILVTL--LLTAL--HLIQSSV-AISCGGCVDLDEINFQKTVERFPYAMVKFDIAFPYGEKHEAFGAFSKAAHKVTKDLLVATVGIKDYGELENKALG
   D_persimilis MMRILVTL--LLAAI--LGRHATW-AISCAGCVDLDEINFQNTVERFPYAVVKFDIAFPYGEKHEAFAAFSKAAHKVTGELLVATVGIKDYGELENKALG
D_pseudoobscura MMRILVTL--LLAAI--LGRHATW-AISCAGCVDLDEINFQNTVERFPYAVVKFDIAFPYGEKHEAFAAFSKAAHKVTGELLVATVGIKDYGELENKALG
   D_willistoni MLRLLLLF--LLTGLNHLGYHATL-AISCPGCVDLDELSFQKTIERFNYTLCKFDIAFPYGDKHEAFAAFSRAAHKVTNDLLIATVGIKDYGEMENKELG
   D_mojavensis MLKLLLTM--LLAAL--LTRRTL--AIACGGCVDLDEINFDKTIVRFPYALVKFDIAFPYGEKHEAFAEFSKAAHKVTAELLVATVGIKDYGEQENKALG
      D_virilis MIKLLLTM--LLAAL--LARHTR--AISCGGCVDLDEINFDKTIVRFPYALVKLDIAFPYGEKHEAFAEFSKAAHKVTDELLVATVGIKDYGELENKALG
    D_grimshawi MIKLMLTLPLLLATL--LAQSPLSDAISCAGCVDLDEINFDKTIARFPYALIKFDIAFPYGEKHEAFAAFSMAAHKATAELLVATVGIKDYGELENKALG
          ruler 1.......10........20........30........40........50........60........70........80........90.......100

                --------------------------------------------------------------------------------------------XXXXXXXX
 D_melanogaster DRYKVDDKNFPSIFLFKGNADEYVQLPSHVDVTLDNLKAFVSANTPLYIGRDGCIKEFNEVLKNYANIPDAEQLKLIEKLQAKQEQLTDP-EQQQNARAY
    D_sechellia DRYKVDDKNFPSIFLFKGNADEYVQLPSHVDVTLDNLKAFVSANTPLYIGRDGCIKEFNEVLKNYANIPDAEQLKLIEELKAKQEQLTDP-EQQQNARAY
     D_simulans DRYKVDDKNFPSIFLFKGNADEYVQLPSHVDVTLDNLKAFVSANTPLYIGRDGCIKEFNEVLKNYANIPDAEQLKLIEELQAKQEQLTDP-EQQQNARAY
       D_yakuba DRYKVDDKNFPSIFLFKGNADEYVQLPSHLDVTVDNLKAFVGANTPLYIGRDGCIKEFNEVLKNYANLPDAEQLTLIKELQTKQEQLTNP-EQQQNAKAY
       D_erecta DRYKVDDKNFPSIFLFKGNAEEYVQLPSHLDVTLDNLKAFVSANTPLYIGRDGCIKEFNEVLKNYANIPDAEQLKLIKELQAKQELLTNP-EQQQNAKAY
    D_ananassae ERYEVDDKNFPAILLFKGKADEFVRFPSHLDVTLDNLKAFVSSNTPLYIGRDGCIKEFNDALKNYANLKDEAQLKLIKQFEAKQEQFTNP-EEQQNAKAY
   D_persimilis ERFEIDDKKFPAILLFKGSVEQYIRFPSHLDVTLDNLKAFVSSNTPLYIGREGCLKEFNDAIKNYANLPDDAQLSLIEELDTQQEKLSKP-EEKLSGKYY
D_pseudoobscura ERFEIDDKKFPAILLFKGSVEQYIRFPSHLDVTLDNLKAFVSSNTPLYIGREGCLKEFNDAIKNYANLPDDAQLSLIEQLETQQEKLSKP-EEKLSGKYY
   D_willistoni DRFGVDQKKFPAIHLFKGNMDEFVKFPDHLDITLDNLKAFVSSNTELYIGRDGCLKQFNDALRNYANKEDSEQLALIKELQAEQEKLTKP-DEQANAKVY
   D_mojavensis ERFKLNEKQFPGIFLFKGNVDEFVKFPAHLDVTLDNLKTFVSSNTPLYIGCDGCLKEFNEALKNYANRPHDEQLALIKELQAKAQELSNKFNEQQNAKVY
      D_virilis ERYKVDEKKFPGIFLFKGSLDDYIQFPAHLDVTLDNLKSFVSSNTALYIGRDGCLKQFNDALKNYANKDDAEQLALIQEMQPQIQQLSKP-DDQQHAKVY
    D_grimshawi ERFKLDEKQLPGIFLFTGSSENYLQFPAHLDVTLDNLKSFVSSNTPLYIGRDGCLKEFNDAIRNYANKPDNEQLAVISEMQLKAAELSLP-QEQQNAKVY
          ruler .......110.......120.......130.......140.......150.......160.......170.......180.......190.......200

                XX-----------------------------------------------------------------
 D_melanogaster LIYMRKIHEVGYDFLEEETKRLLRLKAGKVTEAKKEELLRKLNILEVFRVHKVTKTAPE----KEEL
    D_sechellia LIYMRKIHEVGYDFLEEETKRLLRLKAGKVTEAKKEELLRKLNILEVFRVHKVTKTAPE----KEEL
     D_simulans LIYMRKIHEVGYDFLEEETKRLLRLKAGKVTEAKKEELLRKLNILEVFRVHKVTKTAPE----KEEL
       D_yakuba LIYMRKINELGYDFLEEETKRLLRLKAGKVTEAKKEELLRKLNILEVFRVHKVTKTEQE----KEEL
       D_erecta LIYMRKINEMGYDFLEEETKRLLRLKAGKVTEAKKEELLRKLNILEVFRVHKVTKTASE----KEEL
    D_ananassae LVFMRKIQDQGYGFVEEETKRLLRLKAGKVTETKKLELLRKLNILETFRVHKVTKAPTD----KEEL
   D_persimilis VLYMRKINENGYDFLEEETKRLLRLKAGKVTAAKKLELQQKLNILEAFRVHNLTKAAPKKESSKEDL
D_pseudoobscura VLYMRKINENGYDFLEEETKRLLRLKAGKVTAAKKLELQQKLNILEAFRVHNLTKAAPKKESSKEDL
   D_willistoni LMYMKKLNELGYEFLEEETKRLLRLKAGKVTAAKKVELHHKLNILEAFRVHKLTKAKSQKPEPKKEL
   D_mojavensis LVYMQKINELGYSFVEEETKRLQRLKAGKVTAAKKLELQHRLNILDAFRVHKLTKAAPEKA--KAEL
      D_virilis LLYMQKINEHGYGFVEEETKRLLRLKAGKVTATKKVELQHKLNILEAFRVHKLTKTRPEKA--KAEL
    D_grimshawi LVYMRRINEHGYNFVEQETKRLLRLRAGKVTTAKKLELQQKLNILEAFRVHKLTKSP------KVEL
          ruler .......210.......220.......230.......240.......250.......260.......
```

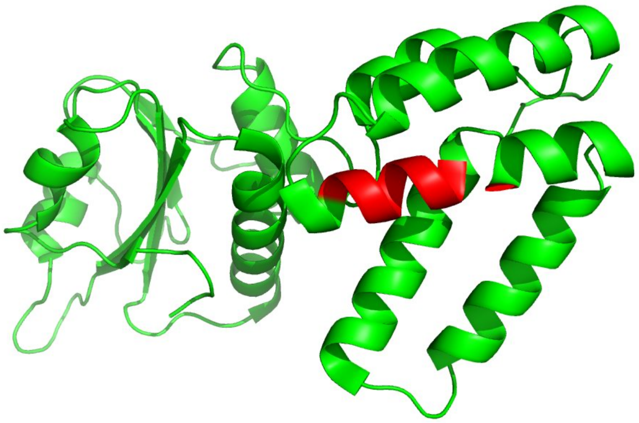

Figure S1.31: Sequence alignment and tertiary structure for gene *l(2)05070*. The mutation cluster is highlighted in red.

D. melanogaster FlyBase ID: FBgn0010590; PDB ID: 1iru; chain ID: H

```
                ----------------------------------------------------------------------------------------------------
D_melanogaster  MQPDFDFTDTPVSTGTTIMAVEFDGGVVIGADSRTSSGAYVANRVTDKLTRITDKVYCCRSGSAADTQAIADIVAYSLNYHENQTNKDALVFEAASEFRN
D_sechellia     MQPDFDFTDTPVSTGTTIMAVEFDGGVVIGADSRTSSGAYVANRVTDKLTRITDKVYCCRSGSAADTQAIADIVAYSLNYHENQTNKDALVFEAASEFRN
D_simulans      -----------------MAVEFDGGVVIGADSRTSSGAYVANRVTDKLTRITDKVYCCRSGSAADTQAIADIVAYSLNYHENQTNKDALVFEAASEFRN
D_yakuba        MLPDFDFTDTPVSTGTTIMAVEFDGGVVIGADSRTSSGAYVANRVTDKLTRITDKVYCCRSGSAADTQAIADIVAYSLNYHENQTNKEALVFEAASEFRN
D_erecta        MLPDFDLTDTPVSTGTTIMAVEFDGGVVIGADSRTSSGAYVANRVTDKLTRITDKVYCCRSGSAADTQAIADIVAYSLNYHENQTNKEALVFEAASEFRN
D_ananassae     MDSGLDFTDTPVSTGTTIMAVEFDGGVVIGADSRTSSGAYVANRVTDKLTRITDKIYCCRSGSAADTQAIADIVAYSLNYYENQTNKDALVHEAASEFRN
D_persimilis    MVYEMDFTDTPVSTGTTIMAVEFDGGVVIGADSRTSSGPYVANRVTDKLTRITDKIYCCRSGSAADTQAIADIVAYSLNYHENQTSKDALVFEAASEFRN
D_pseudoobscura MVYEMDFTDTPVSTGTTIMAVEFDGGVVIGADSRTSSGPYVANRVTDKLTRITDKIYCCRSGSAADTQAIADIVAYSLNYHENQTSKDALVFEAASEFRN
D_willistoni    -MDDFNVTDKPVSTGTTIMAVEFDGGVVVGADSRTSSGAYVANRVTDKLTRITDKIYCCRSGSAADTQAIADIVAYSLNYHEIQTNRDAEVWEAASEFRN
D_mojavensis    MGPDFDFTDTPVSTGTTIMAVEFDGGVVIGADSRTSSGAYVANRVTDKLTQISDKIYCCRSGSAADTQAIADIVAYSLNYHRNQTNKEPLVWEAASEFRN
D_virilis       MGPDIDFTDTPVSTGTTIMAVEFDGGVVIGADSRTSSGAYVANRVTDKLTQISENIYCCRSGSAADTQAIADIVAYSLNYHRNQTNKEPLVCEAASEFRN
D_grimshawi     MGPGFDFTDTPVSTGTTIMAVEFDGGVVIGADSRTSSGAYVANRVTDKLTQISDKIYCCRSGSAADTQAIADIVAYSLNYHRNQTSKEPLVWEAASEFRN
ruler           1.......10........20........30........40........50........60........70........80........90.......100
```

```
                ---------------------------------------------------------XXXXXXXXXXXXXXXX---------------------------
D_melanogaster  YCYSYRESLLAGIIVAGWDEQRGGQVYSIPLGGMLTRESCTIGGSGSSFIYGFVREHYRPNMALEDCVTFVKKAVQHAIYHDGSSGGVVRIGIITKDGIE
D_sechellia     YCYSYRESLLAGIIVAGWDEQRGGQVYSIPLGGMLTREACTIGGSGSSFIYGFVREHYRPNMALEDCVTFVKKAVQHAIYHDGSSGGVVRIGIITKDGIE
D_simulans      YCYSYRESLLAGIIVAGWDEQRGGQVYSIPLGGMLTREACTIGGSGSSFIYGFVREHYRPNMALEDCVTFVKKAVQHAIYHDGSSGGVVRVGIITKDGIE
D_yakuba        YCYSYRESLLAGIIVAGWDAQRGGQVYSIPLGGMLTRESCTIGGSGSSFIYGFVREHYRPNMQLEECVSFVKKAVQHAIYHDGSSGGVVRIGIITKDGIE
D_erecta        YCYSYRESLLAGIIVAGWDEQRGGQVYSIPLGGMLTRESCTIGGSGSSFIYGFVREHYRPNMQLEECVSFVKKAVQHAIYHDGSSGGVVRIGIITKDGIE
D_ananassae     FCYNYRDSLLAGIIVAGYDEERGGQVYSIPLGGMLTREACTIGGSGSSFIYGFVREHYRQNMSKEECVDFVKKAVQHAIYHDGSSGGVVRIGIITKDGIE
D_persimilis    YCYNYRDSLLAGIIVAGWDEQRGGQVFSIPLGGMLTREPCTIGGSGSSFIYGYVREHFHEGMSKEECVKFVKTAVQHAIYHDGSSGGVVRVGIITKEGVE
D_pseudoobscura YCYNYRDSLLAGIIVAGWDEQRGGQVFSIPLGGMLTREPCTIGGSGSSFIYGYVREHFHEGMSKEECVKFVKTAVQHAIYHDGSSGGVVRVGIITKEGVE
D_willistoni    FCYNYRDSLMAGIIVAGWDAQRGGQVYSIPLGGMLTREACTIGGSGSSFIYGFVREHYREGMQKEECVEFVKKAVQHAIYHDGSSGGVVRIGIITEDGIE
D_mojavensis    FCYNYRDSLMAGIIVAGWDEERGGQVYSVPLGGMLTHESCTIGGSGSSYIYGFVKEHYRLGMQMEECVDFVKKAVQHAIYHDGSSGGVVRIGIITKDGIE
D_virilis       YCYNYRDSLLAGIIVAGWDEERGGQVYSVPLGGMLTREGCTIGGSGSSYIYGFVKEHYRPGMPMDECIDFVKKAVQHAIYHDGSSGGVVRIGIITKNGME
D_grimshawi     FCYNYRDSLLAGIIVAGWDEERGGQVYSVPLGGMLTRESCTIGGSGSSFIYGFVREHYRPGMPKEECIEFVKKAVQHAIFHDGSSGGVVRIGIIDKDGIE
ruler           .......110.......120.......130.......140.......150.......160.......170.......180.......190.......200
```

```
                -------------------------------
D_melanogaster  RRIFYNTESGASAVSST--PSFISSE-----
D_sechellia     RRIFYNTESGSSTVSST--PSFISSE-----
D_simulans      RRIFYNTESGASAVSST--PSFISSE-----
D_yakuba        RRIFYNTESGASAVSST--PSFVSAT-----
D_erecta        RRIFYNTESGASAVSST--PSFISSE-----
D_ananassae     RRLFYNTESGDPQVAGT--ASFIAAEQKMEA
D_persimilis    RRLFYNTESGESEISGTGSSAILAQ------
D_pseudoobscura RRLFYNTESGESEISGTGSNAILAQ------
D_willistoni    RRLFFNTETGLPEVSERSESSFIVAA-----
D_mojavensis    RRLFFNTESGQSEIEGS--KSFLV-------
D_virilis       RRLFYNTVTGESTIEGS--QSFIN-------
D_grimshawi     RRLFYNTESGESKIAGS--QSFIN-------
ruler           .......210.......220.......230.
```

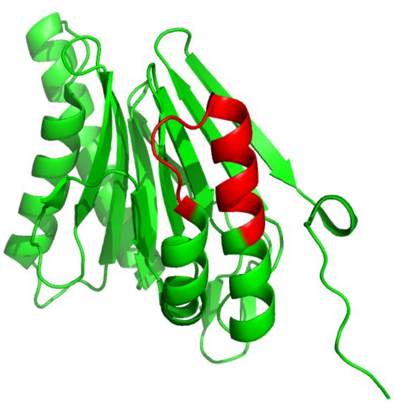

Figure S1.32: Sequence alignment and tertiary structure for gene *α-Man-II*. The mutation cluster is highlighted in red.

D. melanogaster FlyBase ID: FBgn0011740; PDB ID: 1qx1; chain ID: A

```
                ----XXXXXXXX--XXXXXXX---------------------------------------------------------------------------------
D_melanogaster  LSAWHSWDGMAR--IEERLEQARRELSLFQHHDGITGTAKTHVVVDYEQRMQEALKACQMVMQQSVYRLLTKPSIYSPDFSFSYFTLDDSRWPGSGVEDSRT
D_sechellia     LSAWHSWDGMAR--IEERLEQARRELSLFQHHDGITGTAKTHVVVDYEQRMQDALKACQMVMQQSVYRLLTKPSIYSPDFSFSYFTLDDSRWPGSGVEDSRT
D_simulans      LSAWHSWDGMAR--IEERLEQARRELSLFQHHDGITGTAKTHVVVDYEQRMQDALKACQMVMQQSVYRLLTKPSIYSPDFSFSYFTLDDSRWPGSGVEDSRT
D_yakuba        LSAWHSWDGMAR--IEERLEQARRELSLFQHHDGITGTAKTHVVVDYEQRMQEALKACQIVMQQSVYRLLTKPSIYSPDFSFSYFTLDDSRWPGSGVEDSRT
D_erecta        LSAWHSWDGMAR--IEERLEQARRELSLFQHHDGITGTAKTHVVLDYEQRMQEALKACQMVMQQSVYRLLTKPSIYSPDFSFSYFTLDDSRWPGSGVEDSRT
D_ananassae     LSAWHSWDGMAR--IEERLELARRELSLFQHHDGITGTAKTHVVKDYEGRMQEALKACQAVMQQSVYRLLTKPSIYSPEFSFSYFTLDDSRWPGSGVEDSRT
D_persimilis    LSAWNVWPNSTD--FERQLSNARRQLSLFQHHDGITGTAKNHVVLDYEKRMVKALKGCQSVMQQSIYRLLTKPSIYSPDYNFVYFMLDDSRLPGSGVEDLRT
D_pseudoobscura LSAWNVWPNSTD--FERQLSNARRQLSLFQHHDGITGTAKNHVVLDYEKRMVKALKGCQSVMQQSIYRLLTKPSIYSPDYNFVYFMLDDSRLPGSGVEDLRT
D_willistoni    LHSWHHWDEIASGVFDQKLQQARRDLSLFQHHDGITGTAKSHVVIDYEQRLIEALRGCQFVMQQAVYRLLTKPSIYSPDYNFAYFSIDDSRWPGSGVEDSRT
D_mojavensis    LHAWHNWDSAAG--FDRKLEMARREMSLFQHHDGITGTAKTHVMQDYEKRMVEALWACQFVMQQAVYRLLTKPSIYSADFNFYYFTLDDSRWPGVGVEESRT
D_virilis       LHAWQNWDSQAG--FSQKLELARRELSLFQHHDGITGTAKTHVMQDYEKRMIDALKACQFVMQQAVYRLLTKPSIYSADFNFHYFTLDDSRWPGVGVEESRT
D_grimshawi     LHAWQPWDSMAG--FNAKLEQARRELSLFQHHDGITGTAKTHVVQDYEKRLVEALKSCQFVMQQAVYRLLTKPSIYSPDFNFHYFTLDDSRWPGVGVEESRT
ruler           .......520.......530.......540.......550.......560.......570.......580.......590.......600.......610..

                ------------------------------------------------------------------------------------------------------
D_melanogaster  TIILGEDILPSKHVVMHNTLPHWREQLVDFYVSSPFVSVTDLANNPVEAQVSPVWSWHHDTLTKTIHPQGSTTKYRIIFKARVPPMGLATYVLTISDSKPEH
D_sechellia     TIILGEDILPSKHVVMHNTLPHWREQLVDFYVSSPFVSVTDLANNPVEAQVSPVWSWHHDTLTKTIHPQGSTTKYRIIFKARVPPMGLATYVLTISDSKPEH
D_simulans      TIILGEDILPSKHVVMHNTLPHWREQLVDFYVSSPFVSVTDLANNPVEAQVSPVWSWHHDTLTKTIHPQGSTTKYRIIFKARVPPMGLATYVLTISDSKPEH
D_yakuba        TIILGEDILPSKHVVMHNTLPHWREQLVDFYVSSPFVSVTDLTNNPVEAQVSPVWSWHHDTLTKTIHPQGSTTKYRIIFKARVPPMGLATYVLTISDSKPEH
D_erecta        TIILGEDILPSKHVVMHNTLPHWREQLVDFYVSSPFVSVTDLANNPVEAQVSPVWSWHHDTLTKTIHPQGSTTKYRIIFKARVPPMGLATYVLTISDSKPEH
D_ananassae     TIILGEEL-PTKHVVLHNTLPHWREQLVDFYVSSPFISVSDLTNNPVEAQVSPVWSWHHDLLTKTIHPQGSTTKFRLIFKARVPPMGLSTYILTISESKPQH
D_persimilis    TIILGDQL-PTKHVVMHNTLPHWREQLVDFFVSSPFVSVTDSTNNPVEAQVTPVWTWHRDTVTKTTHPQASTTKYRIVFKARVPPMGLSTYLLTVSDSKPEH
D_pseudoobscura TIILGDQL-PTKHVVMHNTLPHWREQLVDFFVSSPFVSVTDSTNNPVEAQVTPVWTWHRDTVTKTTHPQASTTKYRIVFKARVPPMGLSTYLLTVSDSKPEH
D_willistoni    TIILGEEL-PTKHVVIHNTLPRWREQMVDFYVSSPFVSVSDLSGNPIEAQVSPVWSWHHDTLTKTIHPQGSTTKYRLIFQARVPPMGLSTYLLTMADSKPEH
D_mojavensis    TIILGEEL-PTKHVVLHNTVPHWREQLVDFYVSSPFVGVSDLAGHAVEAQISPVWSWHHDTIGKTFNPQGSTTKYRIIFKARVPPMGLTTYILTISSSKPAH
D_virilis       TIILGEEL-PTKHVVLHNTVPHWREQLVDFYVSSPFVSVSDLAGNSVEAQVSPVWSWHHDTISKTVNPQGSTTKYRILFKARVPPMGLTTYLLTVSTSKPAH
D_grimshawi     TIILGDEL-PTKHVVLHNTVPHWREQLVDFYVSSPFVSVSDLAGNAVEAQLSPVWSWHHDTISKTVNPQGSTTKYRIIFKARVPPMGLATYVLTVARSKPAH
ruler           .....620.......630.......640.......650.......660.......670.......680.......690.......700.......710....

                -----------------------------------------------------------------------------------------------XXXXXX-
D_melanogaster  TSYASNLLLRKNPTSLPLGQYPEDVKFGDPREISLRVGN-GPTLAFSEQGLLKSIQLTQDSPHVPVHFKFLKYGVRSHGDRSGAYLFLPNGPASPVELGQPV
D_sechellia     TSYASNLLLRKNPTSLPLGQYPEDVKFGDPREISLRVGN-GPTLAFSEQGLLKSIQLTQDSPHVPVHFKFLKYGVRSHGDRSGAYLFLPNGPASPVELGQPV
D_simulans      TSYASNLLFRKNPTSLPLGQYPEDVKFGDPREISLRVGN-GPTLAFSEQGLLKSIQLTQDSPHVPVHFKFLKYGVRSHGDRSGAYLFLPNGPASPVELGQPV
D_yakuba        TSYASNLLLRKNPTSVPLGLYPEDVKFGDPREISLRVGN-GPTLAFSEQGLLKSIQLTQDSPHVPVHFKFLKYGVRSHGDRSGAYLFLPNGPALPVELGQPV
D_erecta        TSYASNLLLRKNPTSVPLGLYPEDIKFSDPREISLRVGN-GPTLAFSEHGLLKSIQLTQDSPHVPVHFKFLKYGVRSHGDRSGAYLFLPNGPASPVELGQPV
D_ananassae     TSYASNLLMRSTSFAVPLGQYPEDVKFSEHREMSLRVGD-GPTLAFSEDGLLKSMQLSQDSPHVPVHLKFLKYGTRQHGDRSGAYLFLPNGPATPFRVSTPV
D_persimilis    TSYASNLLLRTNPQTVRLGLYPEDVKFGEHRDISLRVGN-GPTLAFSEYGLLKSMQLTPDSEHVPVHLKFLTYGTREHGDRSGAYLFLPNGPATPYPELHDV
D_pseudoobscura TSYASNLLLRTNPQTVRLGLYPEDVKFGEHRDISLRVGN-GPTLAFSEYGLLKSMQLTPDSEHVPVHLKFLTYGTREHGDRSGAYLFLPNGPATPYPELHDV
D_willistoni    TSYASNLLLRTSPISIRLGQYPDDVKFADHREISLRMGNGGPTLAFSEMGVLKSMQLTPDSPHVPVHLKFLKYGTRQHGDHSGAYLFLPNGPASPLLTFGPV
D_mojavensis    TSYATHLLLNNNPLSVNLGQYPEDAKFGEHREFSLRVGT-GPTLAFSEFGMLKSMQLAPGSAPVPVHLKFLKYGTRPHGDKSGAYLFLPNGPATPIVGNTPV
D_virilis       TSYASHLLFNGSPVSVSLGQYPEDTKFSEHSEFSLRVGA-GPILAFSEYGLLKSMQLTPDSSPVPVHLKFLKYGTRTHGDKSGAYLFLPNGPAVPITGNPPV
D_grimshawi     TSYATHLLLNGSPVSVNLGQYPEDAKFGEHREFSLRVGT-GPTLAFSEHGLLKSMQLSADTAPVPAHLKFLKYGTRVHGDKSGAYLFLPNGPATAIVGNPPV
ruler           ...720.......730.......740.......750.......760.......770.......780.......790.......800.......810......
```

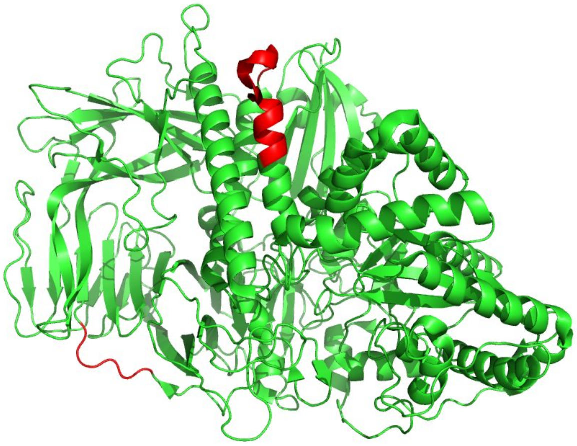

Figure S1.33: Sequence alignment and tertiary structure for gene *Prosα7*. The mutation cluster is highlighted in red.

D. melanogaster FlyBase ID: FBgn0023175; PDB ID: 1iru; chain ID: G

```
                ----------------------------------------------------------------------------------------------------
D_melanogaster  MSTIGTGYDLSASQFSPDGRVFQIDYASKAVEKSGTVIGIRGKDAVVLAVEKIITSKLYEPDAGGRIFTIEKNIGMAVAGLVADGNFVADIARQEAANYR
D_sechellia     MSTIGTGYDLSASQFSPDGRVFQIDYASKAVEKSGTVIGIRGKDAVVLAVEKIITSKLYEPDAGGRIFTIEKNIGMAVAGLVADGNYVADIARQEAANYR
D_simulans      MSTIGTGYDLSASQFSPDGRVFQIDYASKAVEKSGTVIGIRGKDAVVLAVEKIITSKLYEPDAGGRIFTIEKNIGMAVAGLVADGNYVADIARQEAANYR
D_yakuba        MSTIGTGYDLSASQFSPDGRVFQIDYASKAVEKSGTVIGIRGKDAVVLAVEKIISSKLYEPDAGGRIFTIEKNIGMAVAGLVADGNYVADIARQEAANYR
D_erecta        MSTIGTGYDLSASQFSPDGRVFQIDYASKAVEKSGTVIGIRGKDAVVLAVEKIITSQLYEPDAGGRIFTIEKNIGMAVAGLVADGNYVADIARQEAANYR
D_ananassae     MSTIGTGYDLSASQFSPDGRVFQIDYASKAVEKSGTVIGLRGKDCVILAVEKIISSKLHEEDAAGRIFTIEKNIGMAIAGLVADGNYVADIARQEAANFR
D_persimilis    MSTIGTGYDLSASQFSPDGRVFQIDYASKAVEKSGTVIGIRGKDCVILAVEKIITSKLYEPDAAGRIFIVEKNIGMAVAGLVADGNYVADIARQEAANYR
D_pseudoobscura MSTIGTGYDLSASQFSPDGRVFQIDYASKAVEKSGTVIGIRGKDCVILAVEKIITSKLYEPDAAGRIFIVEKNIGMAVAGLVADGNYVADIARQEAANYR
D_willistoni    MSTIGTGYDLSASQFSPDGRVFQIDYASKAVEKWGTVIGLRGKNGVVLAVEKIITSSLHEPDAGGRIFTIERNIGMAVAGLMADANYIADIARQEAANFR
D_mojavensis    MSTIGTGYDLSASQFSPDGRVFQIDYASKAVERSGTVIGIRGKDCVVLAVEKIVADQLYEPDAASRIFTIEKNIGMAIAGLMADGNFVADIARQEAANFR
D_virilis       MSTIGTGYDLSASQFSPDGRVFQIDYASKAVEKSGTVIGIRGKDCVVLAVEEIVVDKLYEVDAARRIFTIEKNIGMAIAGLMADGNFVADIARQEAANFR
D_grimshawi     MSTIGTGYDLSASQFSPDGRVFQIDYASKAVERSGTVIGIRGKDCVVLAVEKIIADQLYEPDAASRIFTIEKNIGMAIAGLMADGNYVADIARQEAANFR
ruler           1.......10........20........30........40........50........60........70........80........90.......100

                -------------------------------------------------------------------------------XXX-XXXXXXXXXXXXXXXXX
D_melanogaster  QQFEQAIPLKHLCHRVAGYVHAYTLYSAVRPFGLSIILASWDEVEGPQLYKIEPSGSSFGYFACASGKAKQLAKTEMEKLKM-DMRTDELVESAGEIIYK
D_sechellia     QQFEQAIPLKHLCDRVAGYVHAYTLYSAVRPFGLSIILASWDEVEGPQLYKIEPSGSSFGYFACASGKAKQLAKTEMEKLKT-DMRTDELVESAGEIIYK
D_simulans      QQFEQAIPLKHLCDRVAGYVHAYTLYSAVRPFGLSIILASWDEVEGPQLYKIEPSGSSFGYFACASGKAKQLAKTEMEKLKT-DMRTDELVESAGEIIYK
D_yakuba        QQFEHAIPLKHLCDRVAGYVHAYTLYSAVRPFGLSIILASWDEVEGPQLYKIEPSGSSFGYFACASGKAKQLAKTEMEKLKT-DMKIDDLVKSAGEIIYK
D_erecta        QQFEHAIPLKHLCDRVAGYVHAYTLYSAVRPFGLSIILASWDEVEGPQLYKIEPSGSSFGYFACASGKAKQLAKTEMEKLKT-DMKIDDLVKSAGEIIYK
D_ananassae     QQFEHAIPLKHLCERVAGYIHAYTLYSAVRPFGLSVIFASWDEVEGPQLYKIEPSGSSFGYYACASGKAKQQAKTEMEKLKP-NMETEELVKSAGEIIYK
D_persimilis    QQFEQVIPLKHLCDRVSAYVHAYTLYSAVRPFGLSIILASWDENDGPQLYKIEPSGSYFGYFACASGKAKQQAKTEMEKSRFVDLNTEELVKKAGEIIYR
D_pseudoobscura QQFEQVIPLKHLCDRVSAYVHAYTLYSAVRPFGLSIILASWDENDGPQLYKIEPSGSYFGYFACASGKAKQQAKTEMEKSRFVDLNTEELVKKAGEIIYR
D_willistoni    QQFEKPIPLKHLCDRVSGYIHAYTLYSAVRPFGISVLLASWDETDGPQLYKIEPSGSSFGYYACASGKAKQQAKTEMEKFKFAEMDTETLVKRAGEIIYR
D_mojavensis    QQFERTIPLKHLCDRVSGYLHAYTLYSAVRPFGLSVILASWDEVDGPQLYKIEPSGNSFGYYACASGKAKQQAKTEMEKFKFSDMTTEELVKSGGKIIYQ
D_virilis       QQFERTIPLKHLCDRVAGYIHAYTLYSAVRPFGLSVIFASWDETDGPQLYKIEPSGTSFGYYACASGKAKQQAKTEMEKYKFADMSTDELVQSGGKIIYQ
D_grimshawi     QQFERTIPLKYLCDRVSGYLHAYTLYSAVRPFGLSVLLASWDEADGAQLYKIEPSGTSFGYYACASGKAKQQAKTEMEKFKFADMNTDELVRSGGKIIYQ
ruler           .......110.......120.......130.......140.......150.......160.......170.......180.......190.......200

                XXXXXXXXXXXXXXXXXXXXXXXXXXXXXXXXXXXXXXXXXX--------------
D_melanogaster  VHDELKDKDFRFEMGLVGRVTGGLHLINPSELTEKARKAGDAANKDEDSDNETH--
D_sechellia     VHDELKDKDFRFEMGLVGRVTGGLHLINPSELTEKARKAGDAANKEEDSDNETH--
D_simulans      VHDELKDKDFRFEMGLVGRVTGGLHLINPSELTEKARKAGDAANKEEDSDNETH--
D_yakuba        VHDDLKDADFRFEMGLVGRVTEGLHLINPPELTEKARNAGDAANKEEDSDNETR--
D_erecta        VHDGLKDKDFRFEMGLVGRVTEGLHLINPPELTEKARNAGDAASTEDDSDNETR--
D_ananassae     VHDDLKDKDFRFEMGLVGRDTNGLHRINPAEYTEIARNAGDAARKDDDSDNETH--
D_persimilis    VHDELKDKEFRFEMGIVGSETQGIYQINPAVLTELARKAGENANKEDDSGDEAN--
D_pseudoobscura VHDELKDKEFRFEMGIVGSETQGIYQINPAVLTELARKAGENANKEDDSGDEAN--
D_willistoni    VHDELKDKNFRFEMGLVAQETQGTHCINHELYTLSARNAGESAKDDDDGSDNEGPQ
D_mojavensis    VHDELKDKDFRFEMGIVGKETKGVHLINSSEWIQKAIEAGEAAKNADDSENEI---
D_virilis       VNDEVNNKDFRFEMGIVGRETNGVHFINHASWSQLAIEAGKAAKTA-DGDNEI---
D_grimshawi     VHDELKDKDFRFEMGIVGKETNGVHLINDASWSQLAIEAGEAAKNADDSENEI---
ruler           .......210.......220.......230.......240.......250......
```

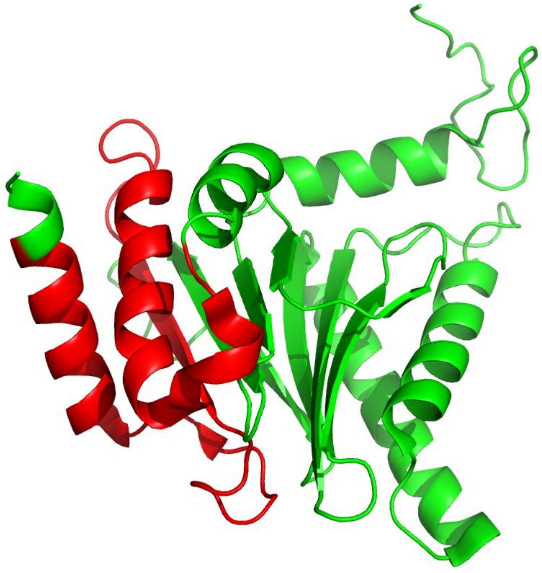

Figure S1.34: Sequence alignment and tertiary structure for gene *CkIIβ2*. The mutation cluster is highlighted in red.

D. melanogaster FlyBase ID: FBgn0026136; PDB ID: 1jwh; chain ID: D

```
                --------------------------------------------------------------XXXXXXXX------------------------------
  D_melanogaster MTDSDESSWIHWFCKQRGNEFFCEVDEEYIQDKFNLNFLDSNVKNYKCALEVILDLNPGSASEDPAEPELEASAEKLYGLIHARFILTNRGIELMLDKYN
     D_sechellia MTDSDESSWIHWFCKQRGNEFFCEVDEEYIQDKFNLNFLDSNVKNYKCALEVILDLNPGSASEDPAEPELEASAEKLYGLIHARFILTNRGIELMLEKYN
     D_simulans MTDSDESSWIHWFCKQRGNEFFCEVDEEYIQDKFNLNFLDSNVKNYKCALEVILDLNPGSASEDPAEPELEASAEKLYGLIHARFILTNRGIELMLEKYN
       D_yakuba MTDSDETSWIHWFCKQRGNEFFCEVDEDYIHDKFNLNFLDSNVTNYRCALEVILDLNSGSASDAPAEPELEASAEKLYGLIHARFILTNRGIELMLEKYN
       D_erecta MTDSDETSWIRWFCKQRGNEFFCEVDEDYIHDKFNLNFLDSNVKNYRCALEVIMDMNSGSASDAPAEPELEASAEKLYGLIHARFILTNQGIELMLEKYN
     D_ananassae MTDSDDTAWIHWFCKQRGNEFFCEVEEDYIHDKFNLNFLDSNVTNYRCALEVILDLNSGSASDQPPEPELEASAEKLYGLIHARFILTNRGIEMMLDKYY
    D_persimilis MTDSDETAWIHWFCRQRGNEFFAMVDEDYIHDKFNLNFLDTDVNNYRCALEVILDLNNGSGSDNVTDADVEASAEKLYGLIHARFILTNRGIDLMLEKFQ
 D_pseudoobscura MTDSDETAWIHWFCRQRGNEFFAMVDEDYIHDKFNLNFLDTDVNNYRCALEVILDLNNGSGSDNVTDADVEASAEKLYGLIHARFILTNRGIDLMLEKFQ
    D_willistoni MTDSEEGAWIHWFCKQRGNEFFCEVDEDYIHDKFNLNFLDSNVNNYRCALEVILDLNSGSGSDIPPEAELEASAEKLYGLIHARFILTNRGIELMLDKFN
    D_mojavensis MSDSEEGAWIPWFCKQRGNEFFCEVDEDYIHDKFNLNFLDSNVSNYRRSLEVILDLEPESGSDPPPEAELEQCAQKLYGLIHARFILTNRGIDLMLEKYQ
      D_virilis MTDSDEGAWIPWFCKQRGNEFFCAIDEDYIHDKFNLNFLDSNVSNYRRALEVILDLDTGSGSDAPPEIELEQCAEKLYGLIHARFILTNRGIELMLEKYQ
     D_grimshawi MTDSEQTAWIHWYCKQRGHEFFCEVDEDYIQDKFNLSFLDSNITNYRRALEVILDLDTGSGSDAPPEAELEQSAEKLFGLIHARFILTNRGIDMMLEKYQ
          ruler 1.......10........20........30........40........50........60........70........80........90.......100

                ----------------------------------------------------------------------------------------------------
  D_melanogaster KGEFGTCPRAFCHSQPVLPIGLSDNPGEDMVRIYCPKCNDVYIPKASRHSNLDGAFFGTGFPHMFFMEKPDARPKRAKQKFVPRLYGFKIHPTAYRTAAE
     D_sechellia KGEFGTCPRIFCHQQPVLPIGLSDNPGEEMVAIYCPKCNDVYIPKASRHSNLDGAFFGTGFPHMLFMEKPDARPKRGKQKFVPRLFGFKIHHTAYRTAAE
     D_simulans KGEFGTCPRVFCHQQPVLPIGLSDNPGEEMVAIYCPKCNDVFIPKASRHSNLDGAFFGTGFPHMLFMEKPDARPKRGKQKFVPRLFGFKIHHTAYRTAAE
       D_yakuba KGEFGTCPRAFCQGQPVLPIGLSDNPGEDMVRIYCPKCNDVYIPKAARHSNLDGAFFGSGFPHMLFMVNPDARPRRTKQKFVPRLYGFKIHQMAYRTLAE
       D_erecta KGEFGTCPRAFCQSQPVLPIGLSDNPGEEMVRIYCPKCNDVYFPKAARHSNLDGAFFGSGFPHMLFMVYPDARPKRTKQKFVPRLYGFKIHQNAYRTTAE
     D_ananassae KGAFGTCPRVFCQRQPVLPIGLTDNPGEEMVRIYCPKCNDVYLPKAARHSNLDGAFFGTGFPHMLFMVNPEARPKRAKQKFVPRLYGFKIHQMAYRSQQD
    D_persimilis KGVFGTCPRVFCQRQHVLPIGLSDNPGDEMVRLYCPKCSDVYLPRSARHANLDGAFFGTGFPHMLFMVNPELRPKRAKAKFVPRLYGFKIHQLAYRSESQ
 D_pseudoobscura KGVFGTCPRVFCQRQHVLPIGLSDNPGDEMVRLYCPKCSDVYLPRSARHANLDGAFFGTGFPHMLFMVNPELRPKRAKAKFVPRLYGFKIHQLAYRSESQ
    D_willistoni KGVFGTCPRVFCQRQPVLPIGLSDNPGEEMVRLYCPKCNDVYVPKSARHANLDGAFFGTGFPHMMFMVNPDARPKRAKQKFVPRLYGFKIHQLAYRPTAD
    D_mojavensis KSEFGICPRVFCQRQPVLPIGLSDNPGEDLVRIFCPKCNDVYAPRSTRHAAIDGAFFGTGFPQMLFMVKPEMRPKRTKQKFVPRLYGFKIHQMALRSLSE
      D_virilis KSAFGICPRVFCQRQPVLPIGLSDNPGEDMVRIFCPKCNDVYVPKSARHAAIDGAFFGTGFPHMLFMVKPEVRPKRTKQRFVPRLYGFKIHQMAYRSVDA
     D_grimshawi KSVFGICPRVYCQRQPVLPVGLSDNPGEDKVRIFCPKCNDVYVPKSTRHSTIDGAFFGTGFPHMLLMVKPEVRPKRSKQKFVPRLYGFKIHPMAYKPLLE
          ruler .......110.......120.......130.......140.......150.......160.......170.......180.......190.......200

                --------------------
  D_melanogaster IQKDV-TMTPVGEIDSPSHI
     D_sechellia NQKDA-TVSPVGEIDSPSHI
     D_simulans NQKDV-TTSPVGEIDSPSHI
       D_yakuba IQK---DMTPIGEIDTPSHN
       D_erecta IQKDI-MMAPIGEIDTPTHN
     D_ananassae PFS---EQPQNREVETPVG-
    D_persimilis GNQ-----KDLQEVET----
 D_pseudoobscura GNQ-----KDLQEVET----
    D_willistoni GTI-----FKEQQQDSH---
    D_mojavensis NIAVS-KDQQPGEGDSN---
      D_virilis NIK-----MPQGDVDSN---
     D_grimshawi PYSASYKDQQHADAESN---
          ruler .......210.......220
```

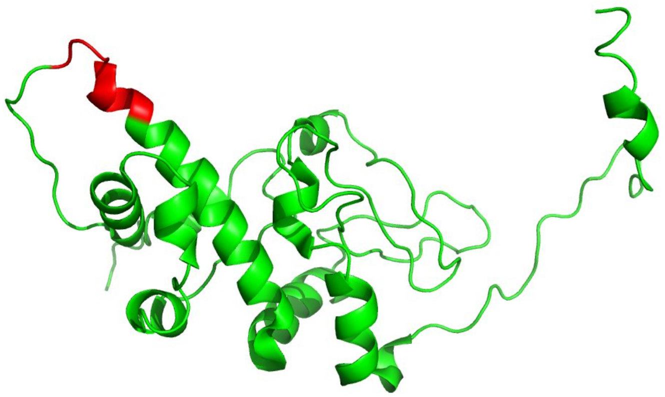

Figure S1.35: Sequence alignment and tertiary structure for gene *GPI*. The mutation cluster is highlighted in red.

Human Ensembl Gene ID: ENSG00000105220; PDB ID: 2cxn; chain ID: A

```
            -------------XXXXXXXXXXXXXXXXXXXXXX-----------------------------------------------------------------
      Human MAALTRDPQFQKLQQWYREHRSELNLRRLFDANKDRFNHFSLTLNTNHGHILVDYSKNLVTEDVMRMLVDLAKSRGVEAARERMFNGEKINYTEGRAVLH
      Chimp MAALTRDPQFQKLQQWYREHGSQLNLRRLFDANKDRFNHFSLTLNTNHGHILVDYSKNLVTEDVMRMLMDLAKSRGVEAARERMFNGEKINYTEGRAVLH
    Macaque MAALTRDPQFQKLQQWYREHGSELNLRRLFDADKDRFNHFSLTLNTNHGHILLDYSKNLVTEDVMRMLVDLAKSRGVEAARERMFNGEKINYTEGRAVLH
      Mouse MAALTRNPQFQKLLEWHRANSANLKLRELFEADPERFNNFSLNLNTNHGHILVDYSKNLVNKEVMQMLVELAKSRGVEAARDNMFSGSKINYTENRAVLH
        Rat MAALTRNPEFQKLLEWHRANSANLKLRELFEADPERFNHFSLNLNTNHGHILLDYSKNLVNKEVLHMLVDLAKSRGVEAARDNMFSGLKINSTEDRAVLH
        Cow MAALTQNPQFKKLKTWYEQHGSDLNLRRLFEGDRDRFNRFSLNLNTNHGHILVDYSKNLVTETVMQMLVDVAKSRGVEAARERMFTGEKINFTEDRAVLH
        Cat MAALTQNPQFLKLQQWYREHGSDLNLRRLF-GDKERFTHF-------------------------------AKSRGVEAARERMFSGEKINFTEDRAVLH
      Chick -MPLSADPHFKKLLEWHKANASKLVLRQLFEADKDRFHKFSLTLNTDHGDILLDYSKNLVTEEVMKMLIELAKSRGVESARERMFSGEKINFTENRAVLH
  Zebrafish -MGLTSDPNFQNLEKWYKSNAANLNMRQMFEEDKTRFQKFSLTLKTDEGDILLDYSKNLINEEVKKMLVELAKSRGVEASRDKMFSGEKINFTEGRAVLH
X_tropicalis -MALSCDPVYQKLSQWYEAHHAGLNMRQMFEADKGRFSKFSKTLVTDHGDILLDYSKNLINEDVMKLLIELAHSRGVESARQKMFSAEKINFTENRAVLH
      ruler 1.......10........20........30........40........50........60........70........80........90.......100

            ----------------------------------------------------------------------------------------------------
      Human VALRNRSNTPILVDGKDVMPEVNKVLDKMKSFCQRVRSGDWKGYTGKTITDVINIGIGGSDLGPLMVTEALKPYSSGGPRVWYVSNIDGTHIAKTLAQLN
      Chimp VALRNRSNTPILVDGKDVMPEVNKVLDKMKSFCQRVRSGDWKGYTGKTITDVINIGIGGSDLGPLMVTEALKPYSSGGPRVWYVSNIDGTHIAKTLAQLN
    Macaque VALRNRSNTPILVDGKDVMPEVNKVLDKMKSFCQRVRSGDWKGYTGKTITDVINIGIGGSDLGPLMVTEALKPYSSEGPRVWYVSNIDGTHIAKTLTQLN
      Mouse VALRNRSNTPIKVDGKDVMPEVNRVLDKMKSFCQRVRSGDWKGYTGKSITDIINIGIGGSDLGPLMVTEALKPYSKGGPRVWFVSNIDGTHIAKTLASLS
        Rat VALRNRSNRSIMMDGKDVMPEVNKVLDKMKSFCQRVRSGDWKGYTGKAITDIINIGIGGSDLGPLMVTEALKPYSKGGPRVWFVSNIDGTHIAKTLANLN
        Cow VALRNRSNAPILVDGKDVMPEVNRVLEKMKSFCQRVRSGEWKGYSGKAITDVINIGIGGSDLGPLMVTEALKPYSSEGPRVWFVSNIDGTHIAKTLATLN
        Cat VALRNRSNTPILVDGKDVMPEVNRVLEKMKSFCQRVRSGDWKGYSGKPITDVINIGIGGSDLGPLMVTEALKPYSSGGPRVWFVSNIDGTHIAKTLATLN
      Chick IALRNRSNTPILVDGKDVVPEVNKVLDKMKHFCQKVRSGEWKGYTGKAITDVVNIGIGGSDLGPLMVTEALKPYSKGGPRVWFVSNIDGTHIAKTLAELH
  Zebrafish VALRNRSNTPIHVDGKDVMPEVNKVLEKMKGFCHKVRSGEWKGFTGKSITDVVNVGIGGSDLGPLMVTEALKPYSKGGPRVWFVSNIDGTHIAKTLAELN
X_tropicalis IALRNRSNTPTLLEGKDVMPEVNAVLEKMKAFCQKVRSGDWKGYTGKAITDVVNIGIGGSDLGPLMVTEALKPYSKGGPRVWFVSNIDGTHIAKTLAELN
      ruler .......110.......120.......130.......140.......150.......160.......170.......180.......190.......200

            ----------------------------------------------------------------------------------------------------
      Human PESSLFIIASKTFTTQETITNAETAKEWFLQAAKDPSAVAKHFVALSTNTTKVKEFGIDPQNMFEFWDWVGGRYSLWSAIGLSIALHVGFDNFEQLLSGA
      Chimp PESSLFIIASKTFTTQETITNAETAKEWFLQVAKDPSAVAKHFVALSTNTTKVKEFGIDPQNMFEFWDWVGGRYSLWSAIGLSIALHVGFDNFEQLLSGA
    Macaque PESSLFIIASKTFTTQETITNAETAKEWFLQAAKDPSAVAKHFVALSTNTTKVKEFGIDPQNMFEFWDWVGGRYSLWSAIGLSIALHVGFDNFEQLLSGA
      Mouse PETSLFIIASKTFTTQETITNAETAKEWFLEAAKDPSAVAKHFVALSTNTAKVKEFGIDPQNMFEFWDWVGGRYSLWSAIGLSIALHVGFDHFEQLLSGA
        Rat PESSLFIIASKTFTTQETITNAETAKEWFLQAAKDPSAVAKHFVALSTNTDKVKEFGIDPKNMFEFWDWVGGRYSLWSAIGLSIALHVGFDHFEQLLSGA
        Cow PESSLFIIASKTFTTQETITNAETAKEWFLLSAKDPSAVAKHFVALSTNTAKVKEFGIDPQNMFEFWDWVGGRYSLWSAIGLSIALHVGFDNFEQLLSGA
        Cat PESSLFIIASKTFTTQETITNAETAKEWFLLSAKDPSAVAKHFVALSTNTTKVKEFGIDPQNMFEFWDWVGGRYSLWSAIGLSIALHVGFDNFEQLLSGA
      Chick PETTLFIIASKTFTTQETITNAVTAKEWFLHAAKDPSAVAKHFVALSTNGPKVKEFGIDTENMFEFWDWVGGRYSLWSAIGLSIALHIGFDNFESLLAGG
  Zebrafish AETTLFIIASKTFTTQETITNAESAKEWFLQAAKDASAVAKHFVALSTNGPKVKDFGIDPENMFEFWDWVGGRYSLWSAIGLSIALHIGYENFEKLLAGA
X_tropicalis PETSLFIIASKTFTTQETITNAETAKEWFLNSAKDASAVAKHFVALSTNAPKVKDFGIDTANMFEFWDWVGGRYSLWSAIGLSIALHIGYDNFEKLLAGA
      ruler .......210.......220.......230.......240.......250.......260.......270.......280.......290.......300
```

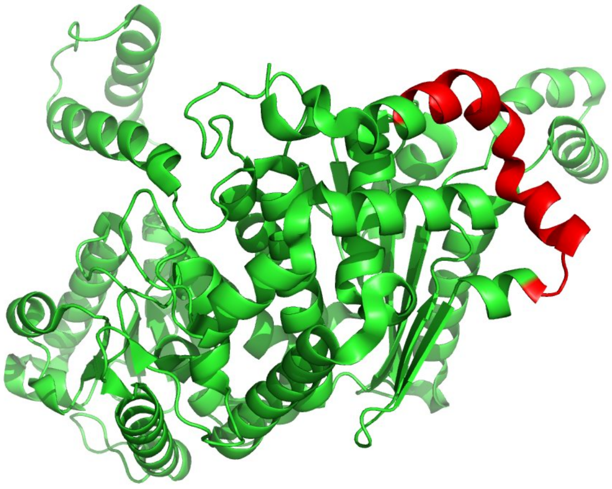

Figure S1.36: Sequence alignment and tertiary structure for gene *NEDD8*. The mutation cluster is highlighted in red.

Human Ensembl Gene ID: ENSG00000129559; PDB ID: 1bt0; chain ID: A

```
             -XXXX------------------------------------------------------------------------------------
       Human MLIKVKTLTGKEIEIDIEPTDKVERIKERVEEKEGIPPQQQRLIYSGKQMNDEKTAADYKILGGSVLHLVLALRGGGGLRQ--------
       Chimp MLIKVKTLTGKEIEIDIEPTDKVERIKERVEEKEGIPPQQQRLIYSGKQMNDEKTAADYKILGGSVLHLVLALRGGGGLRQ--------
     Macaque MLIKVKTLTGKEIEIDIEPTDKVERIKERVEEKEGIPPQQQRLIYSGKQMNDEKTAADYKILGGSVLHLVLALRGGGGLRQ--------
       Mouse MVLEQKTLTGKEIEIDIEPTDKVERIKERVEEKEGIPPQQQRLIYSGKQMNDEKTAADYKILGGSVLHLVLALRGGGGLGQ--------
         Rat MVLEQKTLTGKEIEIDIEPTDKVERIKERVEEKEGIPPQQQRLIYSGKQMNDEKTAADYKILGGSVLHLVLALRGGGGLGQ--------
         Cow MLIKVKTLTRKEIEIDTEPTDKVEQIKERVKEKEGIPPEQQRLTYSGRQINVKKTSTDYRLLSGSVLHLVLALRGGGGLRQ--------
         Cat MLIKVKTLTGKEIEIDIEPTDKVERIKERVEEKEGIPPQQQRLIYSGKQMNDEKTAADYKILGGSVLHLVLALRGGGGLRQ--------
  Zebra_fish MLIKVKTLTGKEIEIDIEPTDKVERIKERVEEKEGIPPQQQRLIYSGKQMNDEKTAADYKIQGGSVLHLVLALRGGQIHQKPDRLLLNM
X_tropicalis MLIKVKTLTGKEIEIDIEPTDKVERIKERVEEKEGIPPQQQRLIYSGKQMNDEKTAADYKIQGGSVLHLVLALRGGC------------
       ruler 1.......10........20........30........40........50........60........70........80.........
```

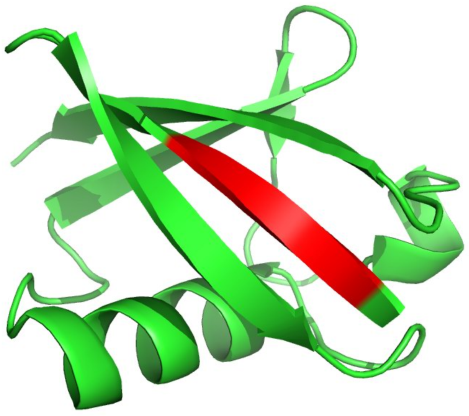

Figure S1.37: Sequence alignment and tertiary structure for gene *CBR3*. The mutation cluster is highlighted in red.

Human Ensembl Gene ID: ENSG00000159231; PDB ID: 2hrb; chain ID: A

```
            ----------------------------------------------------------------------------------------------------
      Human MSSCSRVALVTGANRGIGLAIARELCRQFSGDVVLTARDVARGQAAVQQLQA-EGLSPRFHQLDIDDLQSIRALRDFLRKEYGGLNVLVNNAAVAFKSDD
      Chimp MSSCSRVALVTGANRGIGLAIARELCRQFSGDVVLTARDVARGQAAVQQLQA-EGLSPRFHQLDIDDLQSIRALRDFLRKEYGGLNVLVNNAAVAFKSDD
    Macaque MSSCSRVALVTGANRGIGLAIARELCRQFSGDVVLTARDVARGQAAVQQLQA-EGLSPRFHQLDIDDLQSIRALRDFLRKEYGGLNVLVNNAAVAFKSDD
      Mouse MSSCSRVALVTGANKGIGFAITRDLCRKFSGDVVLTARDEARGRAAVQQLQA-EGLSPRFHQLDIDDPQSIRALRDFLRKEYGGLNVLVNNAGIAFRMDD
        Rat MSSCSRVALVTGANKGIGFAITRDLCRKFSGDVVLTARDEARGRAAVKQLQA-EGLSPRFHQLDIDNPQSIRALRDFLRKEYGGLNVLVNNAGIAFRMDD
        Cow MSSYTRVALVTGANKGIGFAIARDLCREFPGDVVLTARDKARGRAAVQQLQA-EGLSPRFHQLDIDDLQSIRALRDFLRKEYGGLNVLVNNAGIAFKTDD
        Cat MSSCSRVALVTGANKGIGFAIARDLCRQFSGDVVLTARDAARGRAAVQQLQA-EGLSPRFHLLDIDDLQSIRALRDFLRKEYGGLNVLVNNAGIAFQPDD
      Chick IMSNVPVAVVTGSNKGIGLAIVRDLCKQFKGDVYLTARDPARGQEAVAKLQE-EGLHPLFHQLDIDDLQSIKVLRDFLKEKYGGLNVLVNNAGIAFKVSD
  Zebrafish -MSQCKVALVTGANKGIGFAIVRALCKEYTGDVYLSSRDVGRGTAAVDSLKK-EGLHPLFHQLDINDPNSVRTARDFFQEKYGGLDVLINNAGIAFKMAD
X_tropicalis -MASAKVAVVTGGNKGIGLAIVRALCKQFKGDVYLTARDPKLGEEAVRALKEQEGLSPHFHQLDINDLQSIRALGGFLKEKYGGIDVLINNAGIAFKVAD
      ruler 1.......10........20........30........40........50........60........70........80........90.......100

            ----------------------------------------------------------------------------------------------------
      Human PMPFDIKAEMTLKTNFFATRNMCNELLPIMKPHGRVVNISSLQCLRAFEN-CSEDLQERFHSETLTEGDLVDLMKKF-VEDTKNEVHEREGWPNSPYGVS
      Chimp PMPFDIKAEMTLKTNFFATRNMCNELLPIMKPHGRVVNISSLQCLRAFEN-CSEDLQERFHSETLTEGDLVDLMKKF-VEDTKNEVHEREGWPNSPYGVS
    Macaque PMPFDIKAEMTLKTNFFATRNMCNELLPIMKPHGRVVNISSLQCLRAFEN-CSEDLQEKFRSDTLTEGDLVDLMKKF-VEDIKNEVHEREGWPNSPYGVS
      Mouse PTPFDIQAEVTLKTNFFATRNVCTELLPIMKPHGRVVNISSLQGLKALEN-CREDLQEKFRCDTLTEVDLVDLMKKF-VEDTKNEVHEREGWPDSAYGVS
        Rat PTPFDVQAEVTLKTNFFATRNVCTELLPIMKPHGRVVNVSSLQGLKALEN-CSEDLQERFRCDTLTEGDLVDLMKKF-VEDTKNEVHEREGWPDSAYGVS
        Cow PTPFDIQAEMTLKTNFFATRNVCTELLPIVKPHGRVVNVSSSQGSQALEN-CSEDLQEKFRCETLTEEDLVDLMKKF-VEDTKNEVHEREGWPNSAYGVS
        Cat PTPFDIRAEITLKTNFFATRNVCIELLPIIKPHGRVVNISSLEGSKALEN-CSPDLQKKFRCETLTEGDLVDLMKKF-VEDANNEVHEREGWPNSAYGVS
      Chick RTPFAVQAEVTLKTNFFGTRNICTELLPLIKPYGRVVNVSSMVSISALGG-CSQELQKKFRSDTITEDELVELMTKF-VEDTKKSVHEKEGWPNTAYGVS
  Zebrafish TTPFGTQADVTLKTNFFATRDMCNVFLPIIKPGGRLVNVSSGMGSMALGR-CSPELQARFRSDDITEEELNGLMERF-VREAQEGVHSERGWPSTAYGIS
X_tropicalis TTPFGTQAEVTLKTNFFATRDACHELLPLIKPRGRVVNVSSMASYMALGRCCSPELQKVFRSDTITEEELVTLMEKF-VEDAKKGAHQKEGWPNTAYGVS
      ruler .......110.......120.......130.......140.......150.......160.......170.......180.......190.......200

            -------------------------------------XXXXXX-------------------------------------
      Human KLGVTVLSRILARRLDEKRKADRILVNACCPGPVKTDMDGKDSIRTVEEGAETPVYLALLPPDATEPQGQLVHDKVVQNW
      Chimp KLGVTVLSRILARHLDEKRKADRILVNACCPGPVKTDMDGKDSIRTVEEGAETPVYLALLPPDATEPQGQLVHDKVVQNW
    Macaque KLGVTVLSRILARRLDEKRKADRILVNACCPGPVQTDMDGKYSIRTVEEGAESPVYLALLPPDATEPQGQLVHDKVVQNW
      Mouse KLGVTVLTRILARQLDEKRKADRILLNACCPGWVKTDMARDQGSRTVEEGAETPVYLALLPPDATEPHGQLVRDKVVQTW
        Rat KLGVTVLTRILARQLDEKRKADRILLNACCPGWVKTDMARDQGSRTVEEGAETPVYLALLPPDATEPHGQLVRDKVVQTW
        Cow KLGVTVLSRILARRLEEKRKADRILLNACCPGWVKTDLGGAHASRTVEEGAETPVYLALLPPDATEPHGQLVRDKVVQNW
        Cat KLGVTVLSRILARRLDEKRKADRILLNACCPGWVKTDLGGPCGPRTVEEGAETPVYLALLPPDATEPHGQLVHDKVVQNW
      Chick KIGVTVLSRIQARMLNEKRKGDHILLNACCPGWVRTDMAGPKAPKSPEEGAETPVYLALLPSDADGPHGQFVSEKTVRTW
  Zebrafish KTGLTTLTRIQARNLTKERPGDGILCNACCPGWVRTDMAGPNATKSPDEGAITPVYLALLPAGAKEPHGQFVSEMKVQPW
X_tropicalis KVGVTVLSRIQARELNEKRKDDGILLNACCPGWVRTDMAGPKAPKSPDEGAETPVYLALLPNNAHSPHGELVSEKKVVPW
      ruler .......210.......220.......230.......240.......250.......260.......270.......280
```

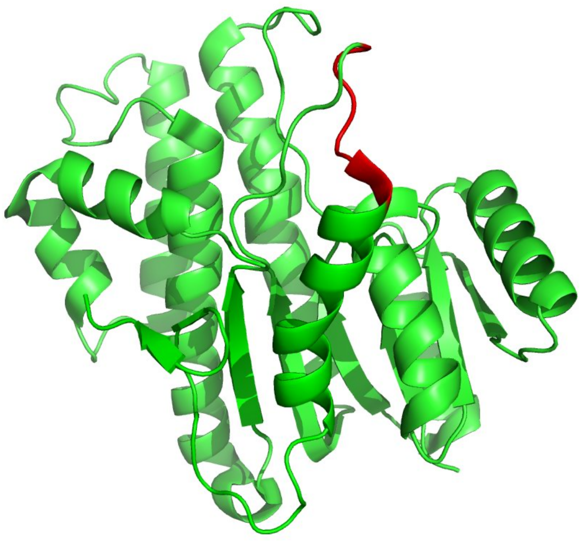

Figure S1.38: Sequence alignment and tertiary structure for gene *LPO*. The mutation cluster is highlighted in red.

Human Ensembl Gene ID: ENSG00000167419; PDB ID: 2gjm; chain ID: A

```
             ----------------------------------------------------------------------------------------------------
       Human PYRTITGDCNNRRKPALGAANRALARWLPAEYEDGLSLPFGWTPGKTRNGFPLPLAREVSNKIVGYLNEEGVLDQNRSLLFMQWGQIVDHDLDFAPDTEL
       Chimp PYRTITGDCNNRRKPALGAANRALARWLPAEYEDGLSLPFGWTPGKTRNGFPLPLAREVSNKIVGYLNEEGVLDQNRSLLFMQWGQIVDHDLDFAPDTEL
     Macaque PYRTITGDCNNRRKPALGAANRALARWLPAEYEDGLSLPFGWTPGKTRNGFPLPLAREVSNKIVGYLNEEGVLDQNRSLLFMQWGQIVDHDLDFAPDTEL
       Mouse PYRTITGYCNNRKNPALGSANRALARWLPAEYEDGLSLPYGWTPGKMRNGFPLPQPREVSNQIAAYLNEEDVLDQKRSMLFMQWGQIVDHDMDFAPETEM
         Rat PYRTITGDCNNRKNPALGSANRALARWLPAEYEDGLSLPFGWTPGKTRNGYPLPQPREVSNQIAGYLNEEDVLDQKRSVLFMQWGQIVDHDLDFAPDTEM
         Cow PYRTITGDCNNRRSPALGAANRALARWLPAEYEDGLALPFGWTQRKTRNGFRVPLAREVSNKIVGYLDEEGVLDQNRSLLFMQWGQIVDHDLDFAPETEL
         Cat -------------------------------------------------------AREVSNKIAGYLSEEGVLDQNTSLLFMQWGQIVDHDLDFAPDTEL
       Chick TYRTITGECNNRKEPHLGVSNHAFARWLPAAYEDGVSVPRGASEGKLYNAFPLPLVRKVSNEIAHTANENITQDQELSLFFMQWGQWVNHDIDLAPSSGM
   Zebrafish KYRTASGVCNNRKNPLLGASNTPFARWLPAQYEDAVSQPKGWDPNKLYNGAALPMVRLVSNRILATADADIESDHDFTFMLTIFGQWVDHDLTFTPFSPS
X_tropicalis TYRTITGECNNRKNPILGASNTGFTRLLPAVYEDGVSLPRGWTENLPINGFPLPLARAVSNEIVRFPNENLTLDQGRALIFMQWGQWTDHDLDLSPETPA
       ruler .......160.......170.......180.......190.......200.......210.......220.......230.......240.......250

             ----------------------------------------------------------------------------------------------------
       Human GSSEYSKAQCDEYCIQGDNCFPIMFPPNDPKAGTQGKCMPFFRAGFVCPTPPYKSL------AREQINALTSFLDASFVYSSEPSLASRLRNLSSPLGLM
       Chimp GSSEYSKAQCDEYCIQGDNCFPIMFPPNDPKAGTQGKCMPFFRAGFVCPTPPYKSL------AREQINALTSFLDASFVYSSEPSLASRLRNLSSPLGLM
     Macaque GSSEYSKAQCDEYCIQGDNCFPIMFPPNDPKARTQGKCMPFFRAGFVCPTPPYKSL------AREQINALTSFLDASFVYSSEPSLASRLRNLSSPLGLM
       Mouse GSDTYTKAQCDEHCIQGDNCFPIMFPPGDPKLKTQGKCMPFFRAGFVCPTPPYKSL------AREQINALTSFLDASLVYSPEPSLANRLRNLSSPLGLM
         Rat GTDNYTKAQCDEHCIQGDNCFPIMFPTGDPKLKTQGKCMSFFRAGFVCPTPPYQSL------TREQINALTSFLDASLVYSPEPSMANRLRNLSSPLGLM
         Cow GSNEHSKTQCEEYCIQGDNCFPIMFPKNDPKLKTQGKCMPFFRAGFVCPTPPYQSL------AREQINAVTSFLDASLVYGSEPSLASRLRNLSSPLGLM
         Cat GSSEYSKAQCDKYCIQGDNCFPIMFPPNDPKVKTQGKCMPFFRAGFVCPTPPYQSL------ARDQINALTSFLDASLVYGPEPSLASRLRNLSSPLGLM
       Chick GAN--PELHCDADCTFRSPCFPIKFPPDDPRMLRSNSCMPFIQSASVCNP---RTF------TREQINAVTSFIDASMVYGSEESVAKSLRNQTNQLGLM
   Zebrafish IRSFSNGIDCENSCERSEPCFPISAPPGDQRLR-PNTCLPVFRSAPTCGSGHTAYMFGEVPNVREQINTLTAYLDAGQVYGSEDGLAKELRDLTNDGGLL
X_tropicalis RSTFLEGIDCDTSCAKQPPCFPLKIPPNDPRIRNQSDCIPLFRSSPVCTP---GSP------VREQINVLTSFLDGSQVYGSDWPLAVKLRNNTNQLGLM
       ruler .......260.......270.......280.......290.......300.......310.......320.......330.......340.......350

             ----------XXXXXXXXX---------------------------------------------------------------------------------
       Human AVNQEVSDHGLPYLPYDSKKPSPCE----FINTTA--RVPCFLAGDSRASEHILLATSHTLFLREHNRLARELKRLNPQWDGEKLYQEARKILGAFVQII
       Chimp AVNQEVSDHGLPYLPYDSKKPSPCE----FINTTA--RVPCFLAGDSRASEHILLATSHTLFLREHNRLARELKRLNPQWDGEKLYQEARKILGAFVQII
     Macaque AVNQEVSDHGLPYLPYDSKKPSPCE----FINTTA--GVPCFLAGDSRASEHILLATSHTLFLREHNRLARELKRLNPQWDGEKLYQEARKILGAFVQII
       Mouse AVNEEVSDNGRPFPPFVKMKPSPCE----VINATA--GVPCFLAGDSRASEQILLATSHTLFIREHNRLATELSRLNPHWDRETLYQEARKIMGAFIQIT
         Rat AVNEEVSDHGRPFPPFVKMKPSPCE----VINATA--GVPCFLAGDSRASEQILLATSHTLFIREHNRLARELSTLNPHWDGETLYQETRKIMGAFIQII
         Cow AVNQEAWDHGLAYLPFNNKKPSPCE----FINTTA--RVPCFLAGDFRASEQILLATAHTLLLREHNRLARELKKLNPHWNGEKLYQEARKILGAFIQII
         Cat AVNQEIWDHGLAYLPFDIKKPSPCE----FINTTA--QVPCFLA-DFRASEHTLLAASHTLLLREHNRLAKELKRLNPHWDGEKLYQEARKILGAFMQII
       Chick AVNQNFTDAGLELLPFENKTKSVCV----LTNKST--NIPCFRAGDKRVTENLGLSALHTVFLREHNRLVTKLGKLNPHWDGEKLYQESRNIIAAMTQII
   Zebrafish RVNNRFKDNGRELLPFTSVNTNLCATRQKILNDSTLTEVPCFIAGDARVNENPALNSLHTLFVREHNRLARALHVLNPTWSSETLYQEARKIVGAFNQIL
X_tropicalis AINQKFTDNGLPFLPFETAEEDFCV----LTNRSS--GIPCFLGGDPRVSEQPGLTAFHTLFVRAHNNIATRLRELNPRWSGETLYQEARKIVGGILQKI
       ruler .......360.......370.......380.......390.......400.......410.......420.......430.......440.......450
```

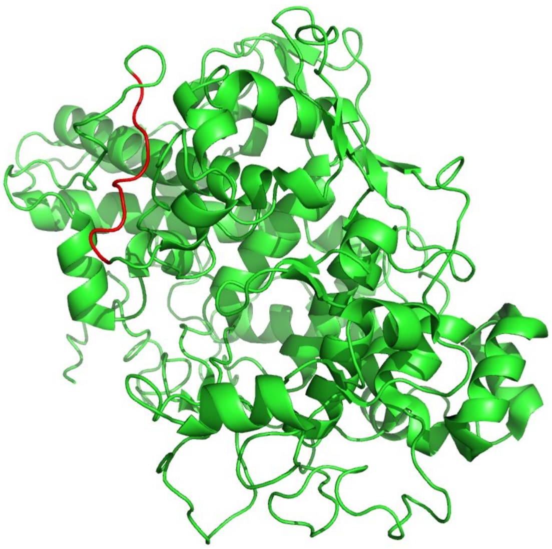

Figure S1.39: Sequence alignment and tertiary structure for gene *NAGA*. The mutation cluster is highlighted in red.

Human Ensembl Gene ID: ENSG00000198951; PDB ID: 1ktb; chain ID: A

```
             ----------------------------------------------------------------------------------------------------
       Human FPHGIPFLADYVHSLGLKLGIYADMGNFTCMGYPGTTLDKVVQDAQTFAEWKVDMLKLDGCFSTPEERAQGYPKMAAALNATGRPIAFSCSWPAYEGGLP
       Chimp FPHGIPFLADYVHSLGLKLGIYADMGNFTCMGYPGTTLDKVVQDAQTFAEWKVDMLKLDGCFSTPEERAQGYPKMAAALNATGRPIAFSCSWPAYEGGLP
     Macaque FPHGIPFLADYVHSLGLKLGIYADMGNFTCMGYPGTTLDKVVQDAQTFAEWKVDMLKLDGCFSTAEERAQGYPKMAAALNATGRPIAFSCSWPAYEGGLP
       Mouse FPHGIAFLADYAHSLGLKLGIYEDMGKMTCMGYPGTTLDKVELDAETFAEWKVDMLKLDGCFSSSRERAEGYPKMAAALNATGRPIAFSCSWPAYEGGLP
         Rat FPHGIAFLADYAHSLGLKLGIYEDMGKMTCMGYPGTTLDKVELDAATFAEWKVDMLKLDGCYSTPKERAEGYPKMAAALNATGRPIAFSCSWPAYEGGLP
         Cow FPHGIAFLADYAHSLGLKLGIYEDLGNFTCMGYPGTTLDKVVQDAQTFAEWKVDMLKLDGCYSTPQERAEGYPKMAAALNATGRPIAFSCSWPAYEGGLP
         Cat FPHGIAFLADYAHSLGLKLGIYEDMGNFTCMGYPGITLDKVTQDAQTFAEWKVDMLKLDGCFSTPEERAMGYPKMAAALNATGRPIAFSCSWPAYEGGLP
       Chick FPRGIKALADYVHARGLKLGIYGDLGRLTCGGYPGTTLDRVEQDAQTFAEWGVDMLKLDGCYSSGKEQAQGYPQMARALNATGRPIVYSCSWPAYQGGLP
   Zebrafish FPRGIPHLAQYVHDRGLKLGIYGDMGTLTCGGYPGTTLDKIETDAQTFADWGIDMLKLDGCYSNSSYQEQGYPMMSKALNATGRPIGYSCSWPAYQGGLP
X_tropicalis FPSGMKALADYVHAKGLKLGIYSDMGNYTCGGYPGTTLDTIKIDADTFASWEVDMLKFDGCYSNSTEKALGYPKMSEALNATGRPILYSCSWPAYEGGLP
       ruler .......110.......120.......130.......140.......150.......160.......170.......180.......190.......200
```

```
             ----------------------------------------------------------------------------------------------------
       Human PRVNYSLLADICNLWRNYDDIQDSWWSVLSILNWFVEHQDILQPVAGPGHWNDPDMLLIGNFGLSLEQSRAQMALWTVLAAPLLMSTDLRTISAQNMDIL
       Chimp PRVNYSLLADICNLWRNYDDIQDSWWSVLSILNWFVEHQDILQPVAGPGHWNDPDMLLIGNFGLSLEQSRAQMALWTVLAAPLLMSTDLRTISAQNMDIL
     Macaque PRVNYSLLADICNLWRNYADIQDSWQSVLSILNWFVKHQDILQPVAGPGHWNDPDMLLIGNFGLSLEQSRAQMALWTVLAAPLLMSTDLRTISAQNMDIL
       Mouse PKVNYTEVSRVCNLWRNYKDIQDSWKSVLSILDWFVRHQDVLQPVAGPGHWNDPDMLLIGNFGLSFDESRAQMALWTVLAAPLLMSTDLRTISPQNMDIL
         Rat PKVNYTEVAGTCNLWRNYKDIQDSWKSVLSILDWFVKHQDILQPVSGPGHWNDPDMLLIGNFGLSFDESRAQMALWTVLAAPLFMSTDLRTISPQNIDIL
         Cow PKVNYTLLADICNLWRNFDDIQDSWRSVLSVLDWFVTHQDVLQPIAGPGHWNDPDMLLIGNFGLSFEQARAQMALWTVLAAPLFMSTDLRTISAQNMDIL
         Cat PKVNYSLLAEICNVWRNYDDIQDSWSSVLSILDWFVDHQDILQPVAGPGHWNDPDMLLIGNFGLSFEQARAQMALWTVLAAPLFMSTDLRTISAQNMDIL
       Chick PKVNYTLLGEICNLWRNYDDIQDSWDSVLSIVDWFFTNQDVLQPFAGPGHWNDPDMLIIGNFGLSYEQSRSQMALWTIMAAPLLMSTDLRTISPSAKKIL
   Zebrafish PKVNYTLLGQICNLWRNYDDIQDSWDSVMGIVDWFFDNQDALQPAAAPGQWNDPDMLIIGDFSLSLDQSRAQMALWSIMAAPLFMSNDLRTISSAARSVL
X_tropicalis PKVNYTQLGSICNMWRNYGDIQDSWDSVLDIIEWYAKNQDVLQPAAGPGRWNDPDMLITGDFGLSYEQSKSQLAIWAILAAPFIMSNDLRTISQDAKDLL
       ruler .......210.......220.......230.......240.......250.......260.......270.......280.......290.......300
```

```
             --------------------------------------------------------------XXXXXXXXXXXXXX-XXXXXXXXX---------------------
       Human QNPLMIKINQDPLGIQGRRIHKEKSLIEVYMRPLSNKASALVFFSCRTD-MPYRYHSSLGQLNFTGSV-IYEAQDVYSGDIISGLRDETNFTVIINPSGV
       Chimp QNPLMIKINQDPLGIQGRRIHKEKSLIEVYMRPLSNKASALVFFSRRTD-MPYRYHSSLGQLNFTGSV-IYEAQDVYSGDIISGLRDETNFTVIINPSGV
     Macaque QNPLMIKINQDPLGIQGRRIHKEKSLIEVYMRPLSNKASALVFFSCRTD-MPYHYHSSLGQLNFTGSV-TYEAQDVYSGDIISGLRVETNFTVIINPSGV
       Mouse QNPLMIKINQDPLGIQGRRILKSKSHIEVFKRYLSNQASALVFFSRRTD-MPFRFHCSLLELNYPKGR-VYEGQNVFTGDIFSGLQTEVNFTVIINPSGV
         Rat QNPLLIKINQDPLGIQGRLIFKSKSHIEVFKRNLSDDASALVFFSRRTD-MPYHFHCSLLELNYPKGS-VYEGQNVFTGDIISGLHPETNFTVIINPSGV
         Cow QNPLMIKINQDPLGIQGRRILKEKSHIEVYLRPLASEASAIVFFSRRMD-MPYHYHSSLARLNFSSSV-VYEAQDVYTGDIISGLQDKTNFTVIINPSGV
         Cat QNPLMIKINQDPLGIQGRRILKEKSHIEVYVRPLADEASALVFFSRRAD-MPYRYHSSLARLNFNSSN-TYEAQNVYTGDVISGLHPKTNFTVVINPSGV
       Chick QNRLMIQINQDPLGIQGRRIIKEGSHIEVFLRPLSQAASALVFFSRRTD-MPFRYTTSLAKLGFPMGA-AYEVQDVYSGKIISGLKTGDNFTVIINPSGV
   Zebrafish QNKAVISINQDPLGIQGRRLLKEKSGIQVFQRPLSKGASALVFXSRRSD-MPYRYTTSLKTLGYQPG--VFEVFDVFSEQRLPELKDSTQFTVSINPSGV
X_tropicalis QNRLLIYINQDSMGKQGNLILRFGS-LEVWKRELMNGQYAVAVLNKGTDGLPKPYSTSLGLLNVTQCTDGYKLYNVFEKEYLGIFKSSTGIDMRVNPTGV
       ruler .......310.......320.......330.......340.......350.......360.......370.......380.......390.......400
```

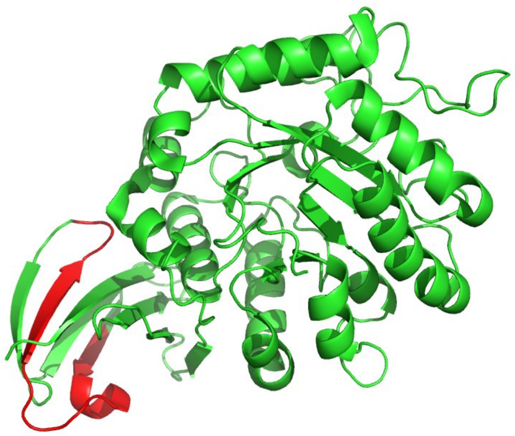

Supplement: Figures S1 — Supporting figures S1.1–S1.39. (5.48 MB PDF) [file pone.0003765.s005.pdf]
